# The Effects of Quinone Imine, a New Potent Nitrification Inhibitor, Dicyandiamide, and Nitrapyrin on Target and Off-Target Soil Microbiota

**DOI:** 10.1128/spectrum.02403-21

**Published:** 2022-07-20

**Authors:** Evangelia S. Papadopoulou, Eleftheria Bachtsevani, Christina V. Papazlatani, Constantina Rousidou, Antonios Brouziotis, Eleni Lampronikou, Myrto Tsiknia, Sotirios Vasileiadis, Ioannis Ipsilantis, Urania Menkissoglu-Spiroudi, Constantinos Ehaliotis, Laurent Philippot, Graeme W. Nicol, Dimitrios G. Karpouzas

**Affiliations:** a Laboratory of Plant and Environmental Biotechnology, Department of Biochemistry and Biotechnology, University of Thessaly, Larissa, Greece; b Laboratory of Environmental Microbiology, Department of Environmental Sciences, University of Thessaly, Larissa, Greece; c Laboratory of Soils and Agricultural Chemistry, Agricultural University of Athens, Athens, Greece; d Laboratory of Soil Sciences, School of Agriculture, Forestry and Environment, Faculty of Agriculture, Aristotle University of Thessaloniki, Thessaloniki, Greece; e Pesticide Science Laboratory, School of Agriculture, Forestry and Environment, Faculty of Agriculture, Aristotle University of Thessaloniki, Thessaloniki, Greece; f Université Bourgogne Franche-Comté, INRAE, AgroSup Dijon, Agroécologie, Dijon, France; g Environmental Microbial Genomics Group, Laboratoire Ampère, École Centrale de Lyon, CNRS UMR 5005, Université de Lyon, Lyon, France; University of Thessaly

**Keywords:** quinone imine, DCD, nitrapyrin, ammonia-oxidizing microorganisms, nitrite-oxidizing bacteria, soil microbial toxicity

## Abstract

Dicyandiamide (DCD) and nitrapyrin (NP) are nitrification inhibitors (NIs) used in agriculture for over 40 years. Recently, ethoxyquin (EQ) was proposed as a novel potential NI, acting through its derivative quinone imine (QI). Still, the specific activity of these NIs on the different groups of ammonia-oxidizing microorganisms (AOM), and mostly their effects on other soil microbiota remain unknown. We determined the impact of QI, and comparatively of DCD and NP, applied at two doses (regular versus high), on the function, diversity, and dynamics of target (AOM), functionally associated (nitrite-oxidizing bacteria-NOB), and off-target prokaryotic and fungal communities in two soils mainly differing in pH (5.4 versus 7.9). QI was equally effective to DCD but more effective than NP in inhibiting nitrification in the acidic soil, while in the alkaline soil QI was less efficient than DCD and NP. This was attributed to the higher activity of QI toward AOA prevailing in the acidic soil. All NIs induced significant effects on the composition of the AOB community in both soils, unlike AOA, which were less responsive. Beyond on-target effects, we noted an inhibitory effect of all NIs on the abundance of NOB in the alkaline soil, with *Nitrobacter* being more sensitive than *Nitrospira*. QI, unlike the other NIs, induced significant changes in the composition of the bacterial and fungal communities in both soils. Our findings have serious implications for the efficiency and future use of NIs on agriculture and provide unprecedented evidence for the potential off-target effects of NIs on soil microbiota.

**IMPORTANCE** NIs could improve N use efficiency and decelerate N cycling. Still, we know little about their activity on the distinct AOM groups and about their effects on off-target soil microorganisms. Here, we studied the behavior of a new potent NI, QI, compared to established NIs. We show that (i) the variable efficacy of NIs across soils with different pH reflects differences in the inherent specific activity of the NIs to AOA and AOB; (ii) beyond AOM, NIs exhibit negative effects on other nitrifiers, like NOB; (iii) QI was the sole NI that significantly affected prokaryotic and fungal diversity. Our findings (i) highlight the need for novel NI strategies that consider the variable sensitivity of AOM groups to the different NIs (ii) identify QI as a potent AOA inhibitor, and (iii) stress the need for monitoring NIs’ impact on off-target soil microorganisms to ensure sustainable N fertilizers use and soil ecosystem functioning.

## INTRODUCTION

Nitrification, the microbial conversion of ammonia (NH_3_) to nitrate (NO^−^_3_), is an essential process of the global nitrogen (N) cycle with paramount importance for plant nutrition and productivity (1). In agricultural soil ecosystems, where N is typically added through the application of inorganic fertilizers, nitrification can result in a significant loss of N, through NO^−^_3_ leaching and N oxides (N_x_O) or elemental N_2_ emissions, raising agricultural production costs and contributing to environmental pollution and climate change (2). It is therefore essential to regulate nitrification in agricultural soils, particularly the first and often rate-limiting step of ammonia oxidation, to prolong the presence of ammonium (NH^+^_4_) in the root zone and facilitate uptake of N by plants (3). In this first step of nitrification, ammonia is oxidized to hydroxylamine by ammonia monooxygenase and further to nitrite (NO^−^_2_) via nitric oxide (4), by canonical ammonia-oxidizing bacteria (AOB), ammonia-oxidizing archaea (AOA), and the recently discovered complete ammonia-oxidizing (comammox) bacteria (5).

The direct inhibition of soil ammonia-oxidizing microorganisms (AOM) using commercial nitrification inhibitors (NIs) is an established strategy for improving N use efficiency in intensive farming systems (6). Hundreds of compounds have been identified as potential NIs (7); however, only three have been widely used in agricultural practice: dicyandiamide (DCD), nitrapyrin (NP; 2-chloro-6-[trichloromethyl] pyridine), and 3,4-dimethylpyrazole phosphate (DMPP) (8). While the underlying inhibition mechanisms of these NIs have been associated with their presumed capacity to interfere with the activation of ammonia monooxygenase, an enzyme carried by all chemolithoautotrophic AOM (9), their efficacy in regulating soil N transformations is highly variable across soils (10, 11). Soil properties such as pH, clay content, and organic matter content (12, 13) and environmental variables such as temperature (14) and moisture (15) are known to affect the performance of NIs in soil. For example, the inhibitory efficiency of DCD and NP in soils with variable physicochemical properties varied from 5.5 to 83.8% for the former (13) and from −39.8 to 135.3% for the latter (16).

To date, the impact of NIs on the indigenous soil microbial communities has been typically considered for target AOM, while little is known about their off-target effects on other functionally associated microbial groups, such as nitrite-oxidizing bacteria (NOB), fueled by substrates produced during ammonia oxidation. Soil studies have shown a trend of selective inhibition of commercial NIs toward AOB, which is further modulated by the application rates and the composition of the metabolically active AOM in soil (17–19). The variable sensitivity of soil AOM to different NIs may arise from fundamental differences in their biochemistry and physiology (1) and their well-documented niche specialization (20) and implies a potential suboptimal efficiency of the NIs currently used in agriculture. This stresses the need for the development of novel nitrification inhibition strategies that could rely on new broad-range NIs, or perhaps on mixtures of NIs with complementary activity against the different AOM groups.

Besides the effects of NIs on target microorganisms, studies on the effects of NIs on nontarget microorganisms are limited. The studies available have explored the effects of DCD- (or DMPP-) fortified fertilizers on the abundance of bacteria or fungi or on microbial community structure determined by low-resolution methods (e.g., PLFAs and DGGE), with contradictory results (21, 22). On the other hand, the potential effects of NP on nontarget soil microorganisms are less studied, with recent reports suggesting possible effects on the overall soil microbial community within and beyond N cycling (23, 24). Careful evaluation of the off-target effects of NIs on the soil microbiota is essential to define potential risks associated with their use in agricultural settings that might compromise soil ecosystem diversity and functioning, beyond nitrification and N cycling.

Ethoxyquin (EQ; 1,2-dihydro-6-ethoxy-2,2,4-trimethylquinoline) is an antioxidant used as a preservative in fruit-packaging plants (25). Previous soil studies have shown that it is rapidly transformed to 2,6-dihydro-2,2,4-trimethyl-6-quinone imine (QI) and suggested a strong inhibitory effect on both AOA and AOB, without, however, affecting soil microbial activity endpoints and PLFA composition (26). A follow-up analysis of the spectrum of the inhibitory activity of EQ and its derivatives, compared to DCD and NP, against a range of soil AOB and AOA strains revealed a selective inhibitory activity of DCD on AOB, whereas NP showed equivalent inhibitory activity against AOB and AOA. EQ showed inhibitory activity against AOA and AOB, comparable to that of NP and DCD, respectively, with its major transformation product QI being the active NI (27). Considering the low cost of EQ (25), and its ability to be transformed in soil to a highly potent NI like QI, there is a major potential for its use in agricultural settings.

The main objective of this study was to determine the efficacy and off-target effects of QI, the active NI component of EQ, in comparison with two established NIs, such as DCD and NP, on the soil microbiota, and to define how these effects are modulated by soil pH and NI application rate. To minimize the involvement of other confounding soil factors on NIs performance, we selectively determined NI effects in two soils that largely differed in their pH (acidic versus alkaline) but had otherwise similar main physicochemical properties (Table 1). We hypothesized that (i) the efficacy of the tested NIs toward nitrification would depend on the functionally dominant AOM groups/phylotypes in each soil, in accord with the pH-driven niche specialization of AOM (20), and the different sensitivity of the distinct AOM groups to the tested NIs (27); (ii) the inhibitory effects of NIs go beyond ammonia oxidation and extend to other soil microbiota components within and beyond N cycling; (iii) high NI dose rates would minimize differences in NIs efficacy and maximize potential off-target effects on soil microbiota. To test these hypotheses, we compared the impact of QI, DCD, and NP, applied at two dose rates, a low one equivalent to the agriculturally recommended application (low dose, *LD*) and a high dose aiming at universal AOM inhibition (high dose, *HD*), on (i) the activity of AOM by monitoring N-pools, potential nitrification rates, and changes in *amoA* gene and transcripts abundance; (ii) the diversity and dynamics of AOM communities and functionally linked NOB, respectively; and (iii) the diversity and dynamics of the total prokaryotic and fungal soil communities. To relate our results to the level and duration of the microbial exposure to these compounds, the dissipation of the NIs in soil was also determined.

**TABLE 1:** Properties of the soils used in this study

Property	Acidic soil	Alkaline soil
Site name	Livadi, Ellasona	Rhodia, Ampelonas
Latitude	40°08'29.7"N	39°46'57.6"N
Longitude	22°10'13.5′E	22°19'36.6"E
Soil order	Cambisol	Cambisol
Soil texture	loam	loam
Sand/silt/clay (%)	40.8/47.8/11.4	45.8/36.0/18.2
Land use	Arable	Arable
Crops/Plants	Lettuce-potato-fallow rotation(One harvest per yr)	Peach (5 sequential growing seasons)
Soil pH (H_2_O)	5.40	7.90
Water Holding Capacity (WHC) (%)	50.32	60.20
Cation-exchange capacity (cmol kg^−1^)	9.3	8.5
Organic C content (%)	2.1	1.8
Organic matter content (%)	4.3	3.6
Total N content (%)	0.13	0.08
Exch. NH^+^_4_-N (mg kg^−1^)	54.01	72.27
NO^−^_3_ -N (mg kg^−1^)	33.40	32.61
Ca (mg kg^−1^)	1182	1164
K (mg kg^−1^)	90	89
Mg (mg kg^−1^)	271	224
Na (mg kg^−1^)	21	18
Olsen-P (mg kg^−1^)	39.1	43.5

## RESULTS

### Dissipation of NIs.

The dissipation patterns of DCD, NP, and QI in the two soils, as described by the single first order (SFO) kinetic model (28), are shown in Fig. S1. DCD showed both a dose-dependent and soil-specific dissipation pattern with dissipation time 50% (DT_50_) values of 23.1 days (acidic soil) and 57.2 days (alkaline soil) in the samples treated with the low dose, and 28.9 days (acidic soil) and 110.2 (alkaline soil) days in the samples treated with the high dose (Table 2). The dissipation of NP and QI did not show a clear dose-dependent pattern. For example, the DT_50_ values of NP in samples treated with the low and the high dose rates were 2.8 and 5.2 days in the acidic and 4.7 and 3.9 days in the alkaline soil, respectively. Similarly, QI DT_50_ values in the acidic and alkaline soil were 2.5 and 3.3 days, respectively, at the low dose rate and 1.8 and 6.8 days at the high dose rate.

**TABLE 2:** The kinetic parameters describing the dissipation of dicyandiamide (DCD), nitrapyrin (NP), and quinone imine (QI) applied at low *(LD)* and high *(HD)* dose rates in the two studied soils (acidic and alkaline)^*a*^

Treatment	Soil	K (d^−1^)	DT_50_ (d)	DT_90_ (d)	χ^2^^*a*^ (%)
DCD *LD*	Acidic	0.030	23.06	76.61	5.88
	Alkaline	0.012	57.20	190.0	3.74
DCD *HD*	Acidic	0.024	28.93	96.09	4.05
	Alkaline	0.006	110.20	366.2	3.64
NP *LD*	Acidic	0.246	2.82	9.38	22.37
	Alkaline	0.133	5.21	17.31	17.13
NP *HD*	Acidic	0.147	4.73	15.71	7.08
	Alkaline	0.178	3.90	12.96	8.81
QI *LD*	Acidic	0.282	2.46	8.17	3.14
	Alkaline	0.210	3.30	10.96	2.68
QI *HD*	Acidic	0.385	1.80	5.98	4.13
	Alkaline	0.102	6.83	22.68	7.95

a The error χ^2^ provides an indication of how accurately the kinetic model describes the dissipation pattern. Error values of 15% suggest acceptable fit to the observed data. Dissipation kinetic parameters were calculated with the single first order (SFO) kinetic model which provided adequate fit to the dissipation data in all cases.

### The impact of NIs on the dynamics of soil inorganic nitrogen.

**FIG 1:**
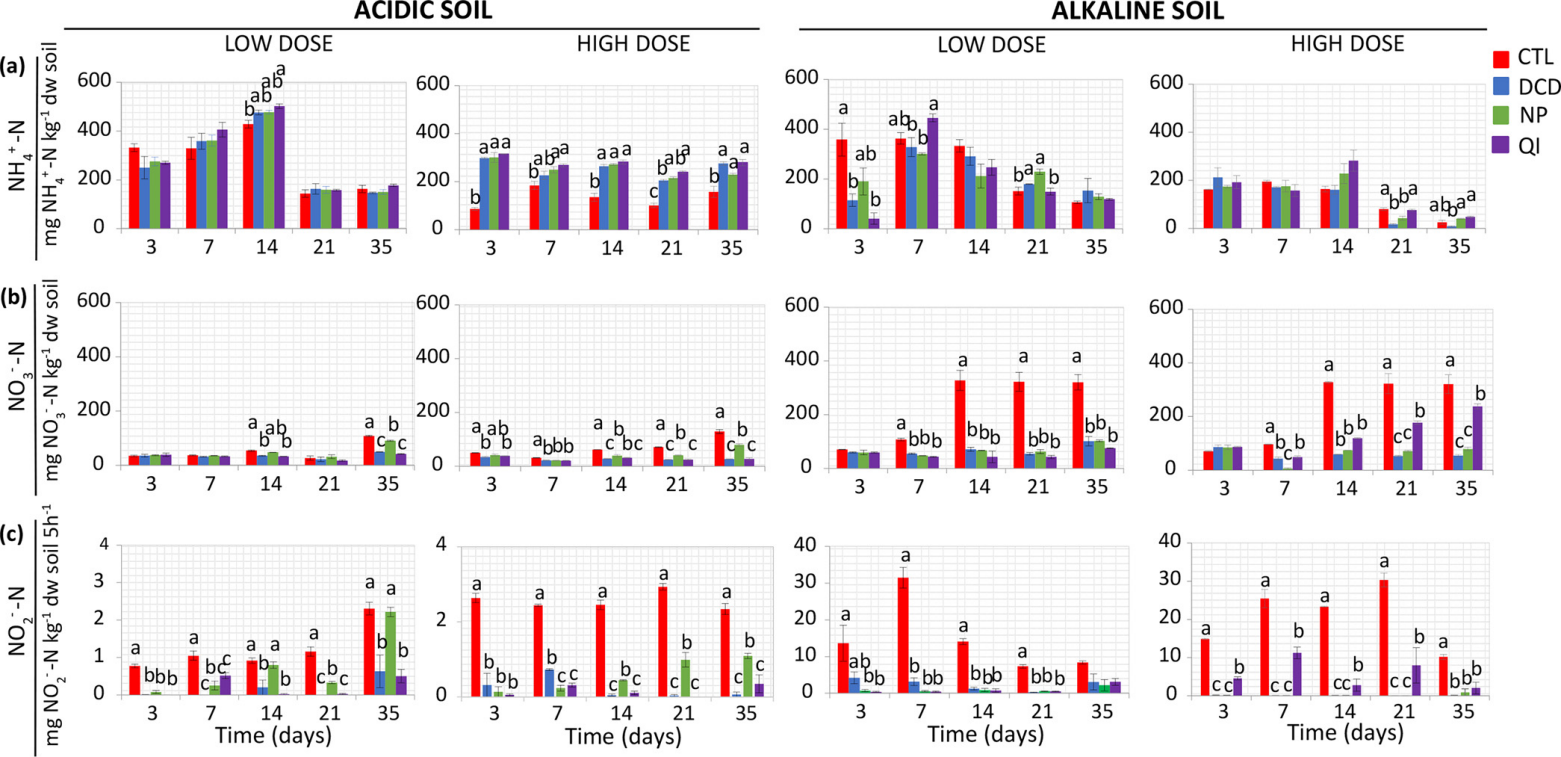
The effect of dicyandiamide (DCD), nitrapyrin (NP), and quinone imine (QI), applied at two dose rates (low and high), on ammonium concentration (a), nitrate concentration (b), and potential nitrification (PN) (c) in an acidic and alkaline soil. Each value is the mean of three replicates ± standard error. At each time point, groups designated by the same letter are not significantly different at the selected *P*-value levels.

We first determined the effects of NIs on inorganic nitrogen pools and potential nitrification as measures of potential effects on nitrification activity. In both soils, the low dose rate of the NIs did not induce a clear temporal pattern in NH^+^_4_-N levels, unlike their high dose rate, which resulted in a significant (*P* < 0.001) and persistent increase of NH^+^_4_-N levels only in the acidic soil (Fig. 1a).

NO^−^_3_-N, DCD ,and QI low dose rates induced a significant reduction at days 14 and 35 in the acidic soil, whereas at high dose rates all NIs induced a significant (*P* < 0.001) and persistent decrease, with NP showing the lowest and least persistent effect (Fig. 1b). In the alkaline soil, all tested NIs at both dose rates significantly (*P* < 0.05) decreased NO^−^_3_-N formation from day 7 onward (Fig. 1b).

DCD and QI exerted a significant and persistent inhibitory effect (*P* < 0.001) on potential nitrification in the acidic soil, whereas the effect of NP was weaker and less persistent, especially at the low dose rate (Fig. 1c). In the alkaline soil, both NI doses induced a significant decrease (*P* < 0.001) in potential nitrification, with recovery observed only at the low dose rates at 35 days (Fig. 1c). QI at the high dose rate showed a weaker inhibitory effect (*P* < 0.05) compared to DCD and NP, still being significantly lower than the control (Fig. 1c).

### The impact of NIs on the abundance and transcriptional activity of AOs.

In parallel we monitored, via q-PCR and RT-q-PCR, the effects of NIs on the abundance and transcriptional activity of AOB, AOA, and comammox bacteria. In the acidic soil, regardless of the treatments, the abundance of the AOA *amoA* genes (2.2 × 10^6^ to 3.0 × 10^7^ g^−1^ d.w. soil) was significantly higher than that of AOB (9.2 × 10^4^ to 8.8 × 10^5^ g^−1^ d.w. soil). Conversely, in the alkaline soil the AOB *amoA* genes (3.5 × 10^7^ to 2.4 × 10^8^ g^−1^ d.w. soil) were more abundant (*P* < 0.05) than those of AOA (7.3 × 10^5^ to 2.1 × 10^7^ g^−1^ d.w. soil). Comammox clade A and B *amoA* genes were not detected in either soil.

**FIG 2:**
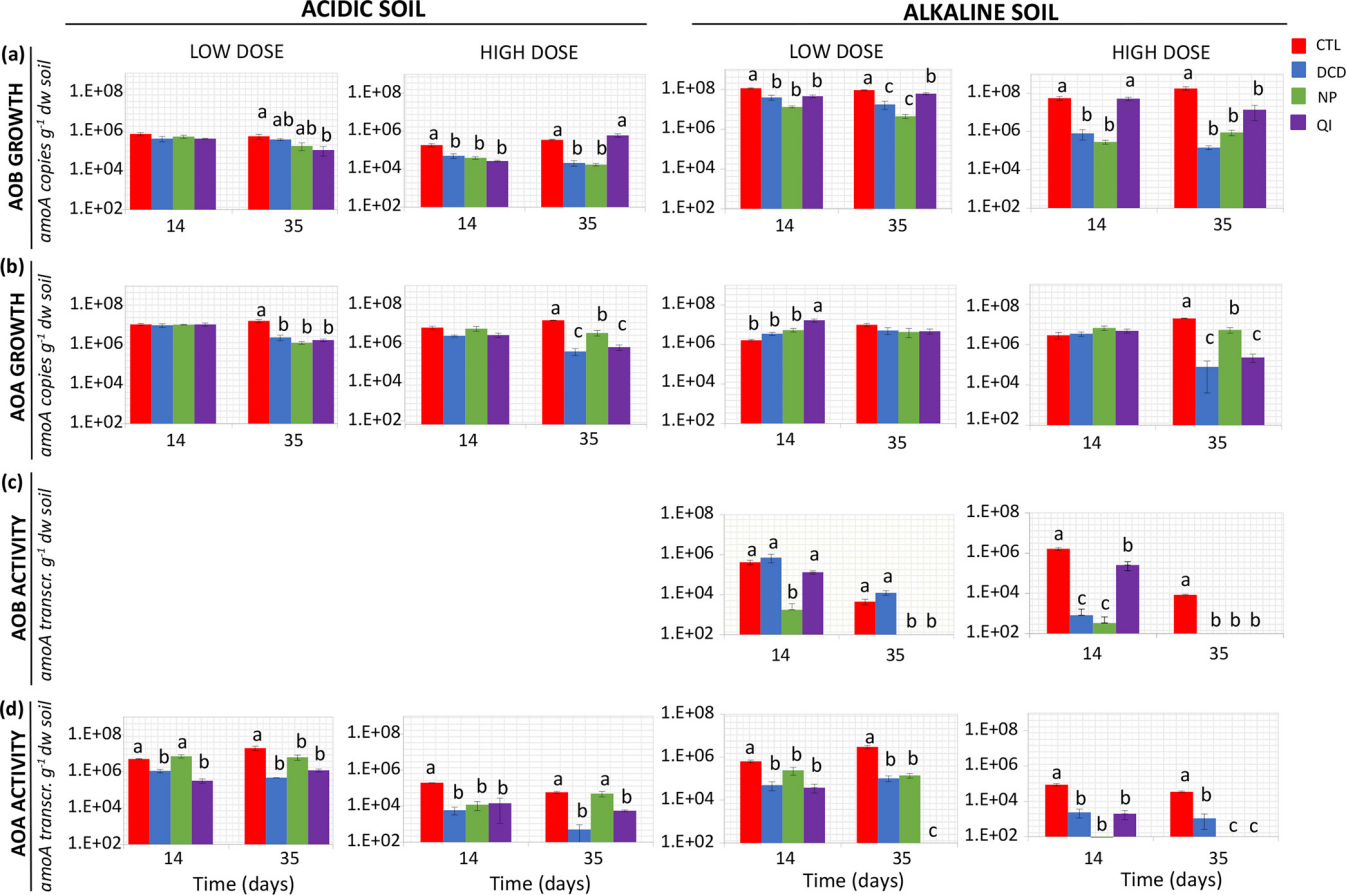
The effect of dicyandiamide (DCD), nitrapyrin (NP), and quinone imine (QI), applied at two dose rates (low and high), on the abundance of the *amoA* genes and transcripts of AOB (a, c) and AOA (b, d), in an acidic and alkaline soil. Each value is mean of three replicates ± standard error. All values are expressed in log scale. At each time point, groups designated by the same letter are not significantly different at the selected *P*-value levels. In the acidic soil bacterial *amoA* gene transcripts were below the limit of detection.

In the acidic soil, QI induced a significant reduction (*P* < 0.05) in AOB abundance compared to the control on days 35 (low dose) and 14 (high dose). DCD and NP induced a significant (*P* < 0.05) and persistent reduction in AOB abundance only at the high dose rate (Fig. 2a). Regarding AOA, both doses of NIs significantly decreased (*P* < 0.05) their abundance at day 35, with the high NP dose showing significantly lower inhibition (*P* < 0.05) compared to DCD and QI (Fig. 2b). Bacterial *amoA* gene transcripts were below the limit of detection (<48 gene transcripts g^−1^ d.w. soil). Regarding AOA, both doses of DCD and QI induced a significant and persistent reduction (*P* < 0.05) of *amoA* gene transcripts, while NP showed a weaker and temporary inhibition of *amoA* transcription (*P* < 0.05) (Fig. 2d).

In the alkaline soil, all NIs at both dose rates significantly and persistently reduced (*P* < 0.05) the abundance of AOB (Fig. 2a). Regarding AOA, we observed a reduction in their abundance (*P* < 0.05) in the samples treated with the high NI dose rates only at 35 days (Fig. 2b). At the transcription level, NP and QI reduced (*P* < 0.05) AOB *amoA* gene transcript abundance at both dose rates, with the impact of the QI low dose only being significant (*P* < 0.05) on day 35. DCD induced a significant reduction (*P* < 0.05) of AOB *amoA* gene transcripts only at the high dose rate (Fig. 2c). Regarding AOA, the application of NIs at both dose rates induced a significant and persistent reduction (*P* < 0.05) of *amoA* gene transcripts (*P* < 0.001) (Fig. 2d).

### The impact of NIs on the dynamics of NOB.

We expanded our tests to NOB, functionally associated with AOM. In the acidic soil, all tested NIs induced a significant reduction on the abundance of the *nxrB* gene of *Nitrobacter* and *Nitrospira* at day 35 (*P* < 0.05) at the low dose rate. In the alkaline soil, all NIs significantly (*P* < 0.05) decreased the abundance of the *nxrB* gene of *Nitrobacter* at both dose rates, compared to *Nitrospira,* whose abundance was negatively affected only at the high dose rates of DCD and QI (Fig. S2).

### The impact of NIs on the abundance of bacteria, fungi, and Thaumarchaeota.

Beyond N cycling, we investigated, via q-PCR, the impact of NIs on the abundance of total bacteria, fungi, and Thaumarchaeota. DCD, NP, and QI, at both rates, significantly and persistently decreased (*P* < 0.05) the abundance of Thaumarchaeota in the acidic soil but not of bacteria and fungi. In the alkaline soil, NIs did not show a clear temporal dose-dependent effect on the abundance of any of the studied microbial groups (Fig. S3). The ratio of AOA *amoA* to thaumarcheotal 16S rRNA gene abundance ranged from 0.5 to 192.6 in the acidic soil (median 16.8), and from 0.02 to 113.9 in the alkaline soil (median 5.7). The resulting ratios of *amoA*: thaumarcheotal 16S rRNA did not vary significantly between NI treatments (*P* > 0.05) and increased only in the acidic soil at day 14 upon application of NP (low dose rate) and QI (high dose rate) (Table S1).

### The impact of NIs on the diversity of the different soil microbial groups.

Eventually we determined the impact of NIs on the diversity of (i) target microorganisms (AOM) via amplicon sequencing of the *amoA* gene of AOA and AOB and (ii) the total prokaryotic and fungal community via amplicon sequencing of the 16S rRNA and ITS, respectively. The per-sample average sequence numbers obtained and passing quality control screening are presented in Table S2. Our sequencing effort provided adequate coverage of the microbial diversity as evidenced by rarefaction curves that reached a plateau in all samples and for all microbial domains (Fig. S4).

The two tested soils supported similar bacterial and fungal communities at the phylum level. The soil bacterial communities were composed of Proteobacteria (39.5 and 44.6%, in the acidic and alkaline soil, respectively), Actinobacteria (16.6 and 15.0%), Gemmatimonadota (9.7 and 7.5%), Acidobacteriota (10.7 and 9.0%), and Chloroflexi (7.4 and 5.3%) (Fig. S5). NI-driven changes in the relative abundance of the bacterial phyla were evident with QI (both dose rates) significantly increasing the relative abundance of Proteobacteria (*P* < 0.001) compared to the control, while other phyla like Gemmatimonadota were significantly decreased upon application of QI (*P* < 0.001). The fungal community was dominated by *Ascomycetes* (84.7 and 85.6%) (orders Sordariomycetes (38.7 and 56.7%), Dothideomycetes (9.5 and 17.3%), Eurotiomycetes (15.0 and 4.5%), Leotiomycetes (15.2 and 0.4%), and *Basidiomycetes* (11.3 and 9.6%) (Fig. S6). In the acidic and alkaline soil AOB *amoA* amplicon sequencing analysis showed a dominance of *Nitrosospira* (89.2 and 43.4%, respectively) and unclassified *amoA* sequences (10.8 and 56.6%, respectively), with *N. briensis* (11.3 and 34.3%, respectively) identified as the only characterized *Nitrosospira* cluster, according to Abell et al. (29) (Fig. S7, Table S3). Regarding AOA, the two soils supported distinct communities, with the acidic soil dominated by *Nitrososphaerales* γ clade (γ-2.2. [69.1%] and γ-2. [26.1%]), and the alkaline soil dominated by *Nitrososphaerales* α, γ, and ε clades (γ-2.1. [34.3%], ε-2.2. [22.8%], *α*-3.1. [10.2%], *γ*-1.1. [8.8%]) (Fig. S8, Table S4).

**(i) Effects on α-diversity.** The diversity of the bacterial community was only affected by the high dose rate of QI in the alkaline soil (Inverse Simpson index and Pielou’s evenness, *P* < 0.05), whereas QI had a significant effect at both doses on the diversity of the fungal community in the acidic soil only. DCD applied at the high dose rate also significantly decreased (*P* < 0.05) the Shannon index for fungi in the acidic soil. The observed richness of AOB was only affected by the low dose rate of QI (*P* < 0.05) in the acidic soil, whereas QI at both dose rates and NP at the high dose rate significantly reduced the observed richness of AOB (*P* < 0.05) in the alkaline soil. NP and QI, at both dose rates, reduced the α-diversity of AOB in the acidic (Inverse Simpson, *P* < 0.05) and the alkaline (Shannon, Inverse Simpson and Pielou’s evenness, *P* < 0.05) soil. In contrast, DCD significantly reduced the α-diversity of AOB only at the high dose rate in the acidic (Inverse Simpson, *P* < 0.05) and the alkaline (Inverse Simpson and Pielou’s evenness, *P* < 0.05) soil. For AOA, we noted a significant reduction at Pielou’s evenness values in the alkaline soil by the high dose rate of all NIs (Table S5).

**FIG 3:**
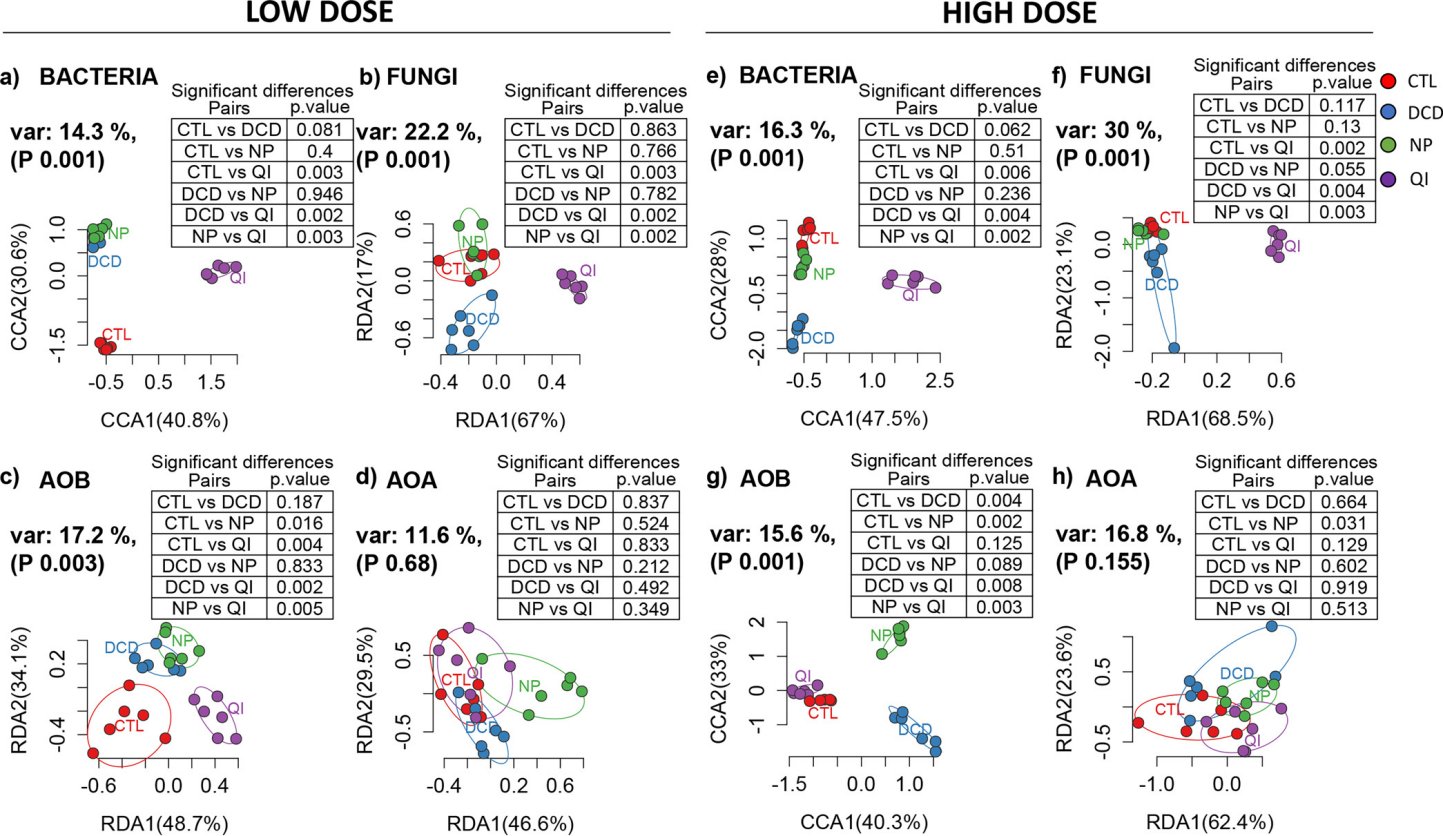
Canonical correspondence analysis (CCA) or redundancy analysis (RDA) of bacterial (a, e), fungal (b, f), AOB (c, g), and AOA (d, h) community structures in samples of the acidic soil treated with the low (a, b, c, d) or the high (e, f, g, h) dose rates of the three NIs (DCD, QI, NP) and in untreated samples (CTL). The percentages of the plotted canonical variance and *P*-values of the multilevel pairwise PERMANOVA are provided next to the axes and at the top-right tables, respectively. Ellipses encompass all samples of each treatment.

**FIG 4:**
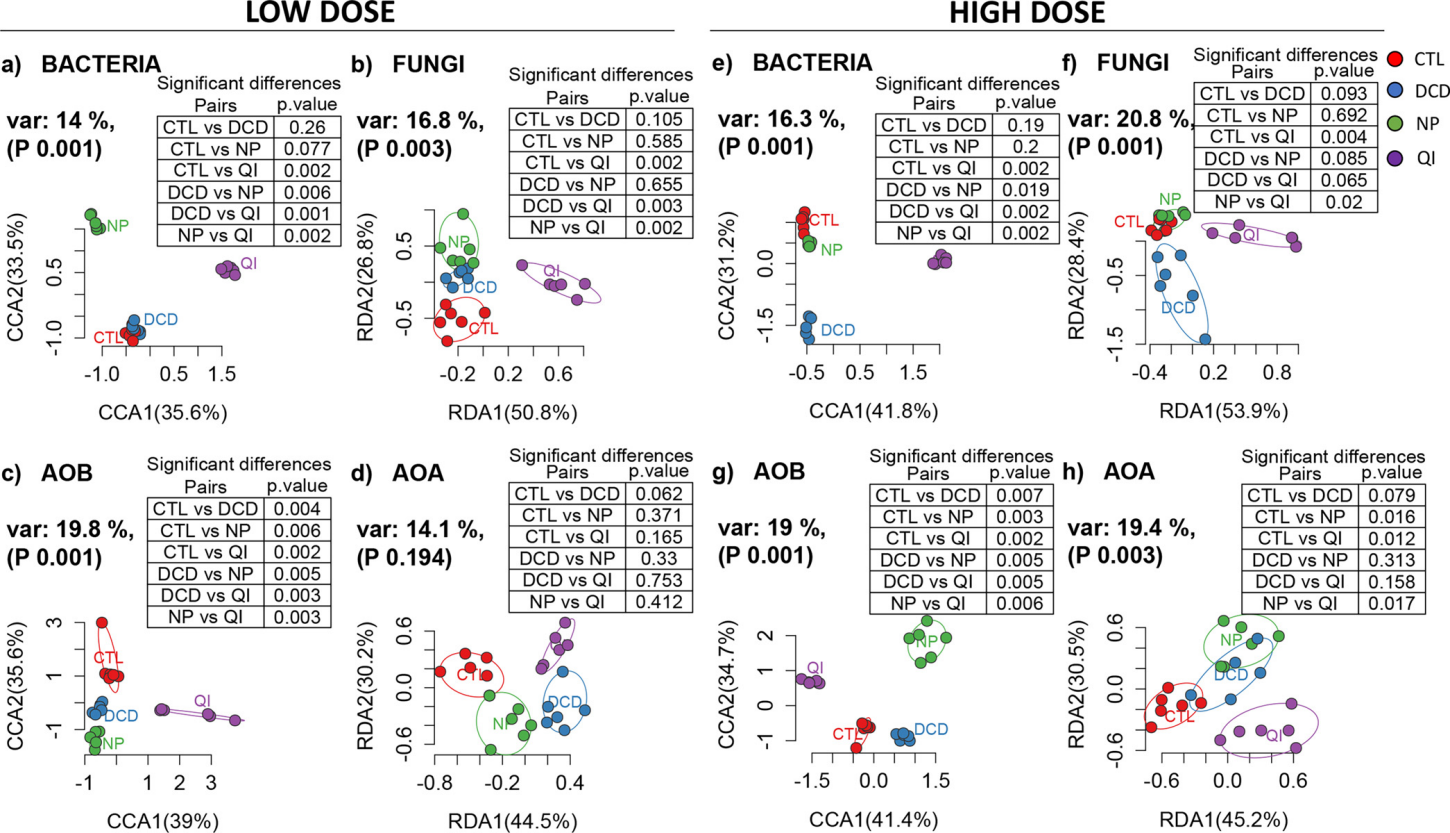
Canonical correspondence analysis (CCA) or redundancy analysis (RDA) of bacterial (a, e), fungal (b, f), AOB (c, g), and AOA (d, h) community structures, in samples of the alkaline soil treated with the low (a, b, c, d) or the high (e, f, g, h) dose rates of the three NIs (DCD, QI, NP) and in untreated samples (CTL). The percentages of the plotted canonical variance and the *P*-values of the multilevel pairwise PERMANOVA are provided next to the axes and at the top-right tables, respectively. Ellipses encompass all samples of each treatment.

**(ii) Effects on β-diversity.** We employed multivariate analysis to determine the effects of NIs on the β-diversity of the different microbial groups studied. Canonical correspondence analysis (CCA) or redundancy analysis (RDA) (not considering the time factor) coupled with pairwise permutational multivariate analysis of variance (PERMANOVA) showed that, for the NIs tested, only QI induced a significant (*P* ≤ 0.006) and universal (dose-wise) effect on the structure of the bacterial and fungal communities in both soils (Fig. 3 and 4). Regarding AOB, all NIs significantly affected their community structure (*P* < 0.05), except for the low dose rate of DCD and the high dose rate of QI in the acidic soil (Fig. 3 and 4). Conversely, the AOA community was less responsive with significant changes only induced by the high dose rates of NP in both soils and QI only in the alkaline soil (Fig. 3 and 4).

**FIG 5:**
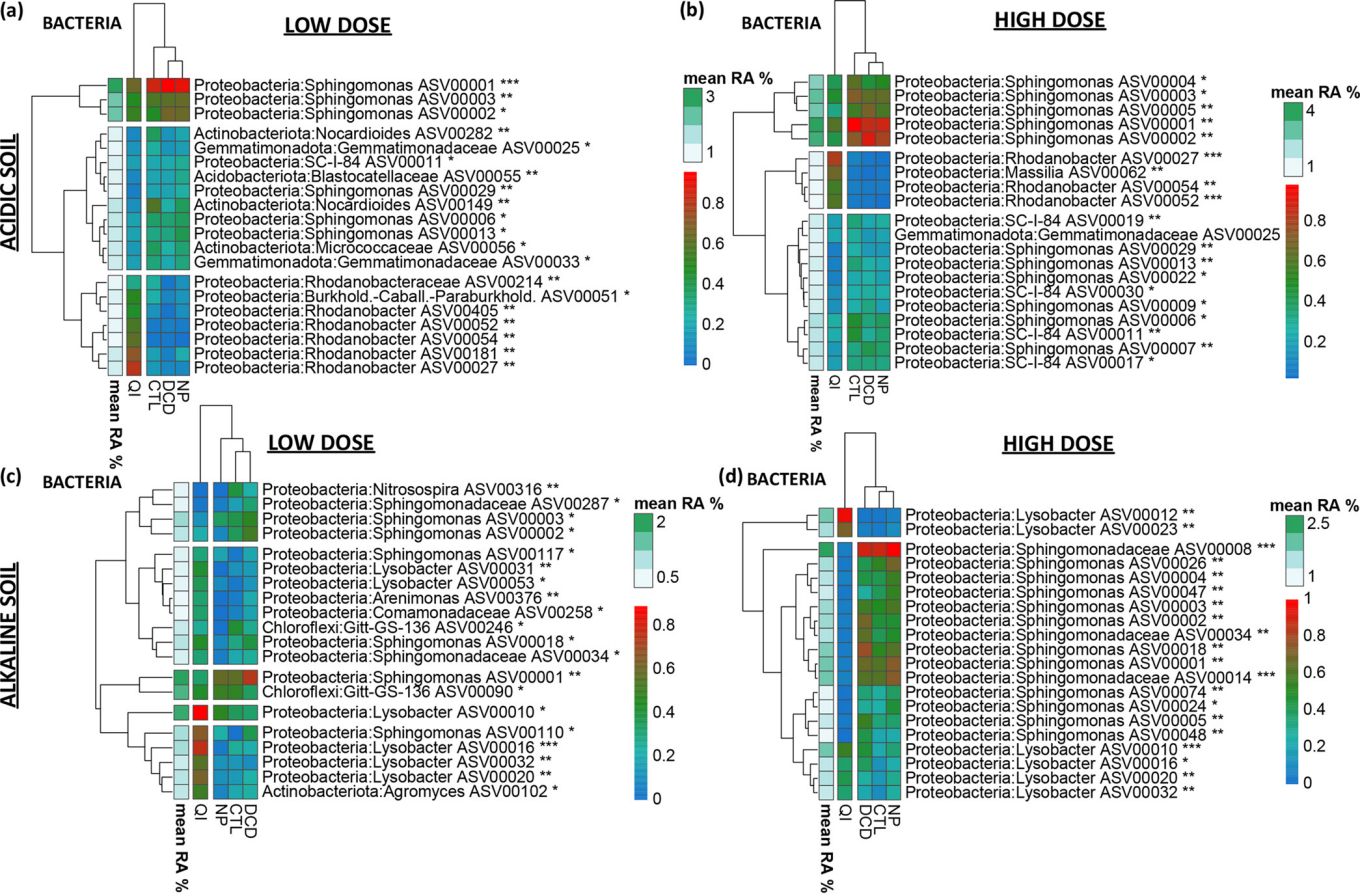
Heatmaps of differentially abundant bacterial ASVs (Kruskal-Wallis and Wilcoxon rank sum analysis, test significance is indicated in each ASV annotation * *P* < 0.05, ** *P* < 0.01, *** *P* < 0.001), scaled from 0 to 1 (blue to red)) in the acidic (a, b) and alkaline (c, d) soil untreated (CTL) or treated with a low (a, c) or high (b, d) dose rate of NIs (DCD, QI, NP). The mean relative abundance of each ASV is indicated by the white-to green column and key. Hierarchical clustering was performed for depicting treatment and ASV groupings. Per ASV, barplots with the corresponding *P*-values/ÀSV taxonomies/treatment relative abundances are provided in Fig. S9 to S12 in the supplemental material.

**FIG 6:**
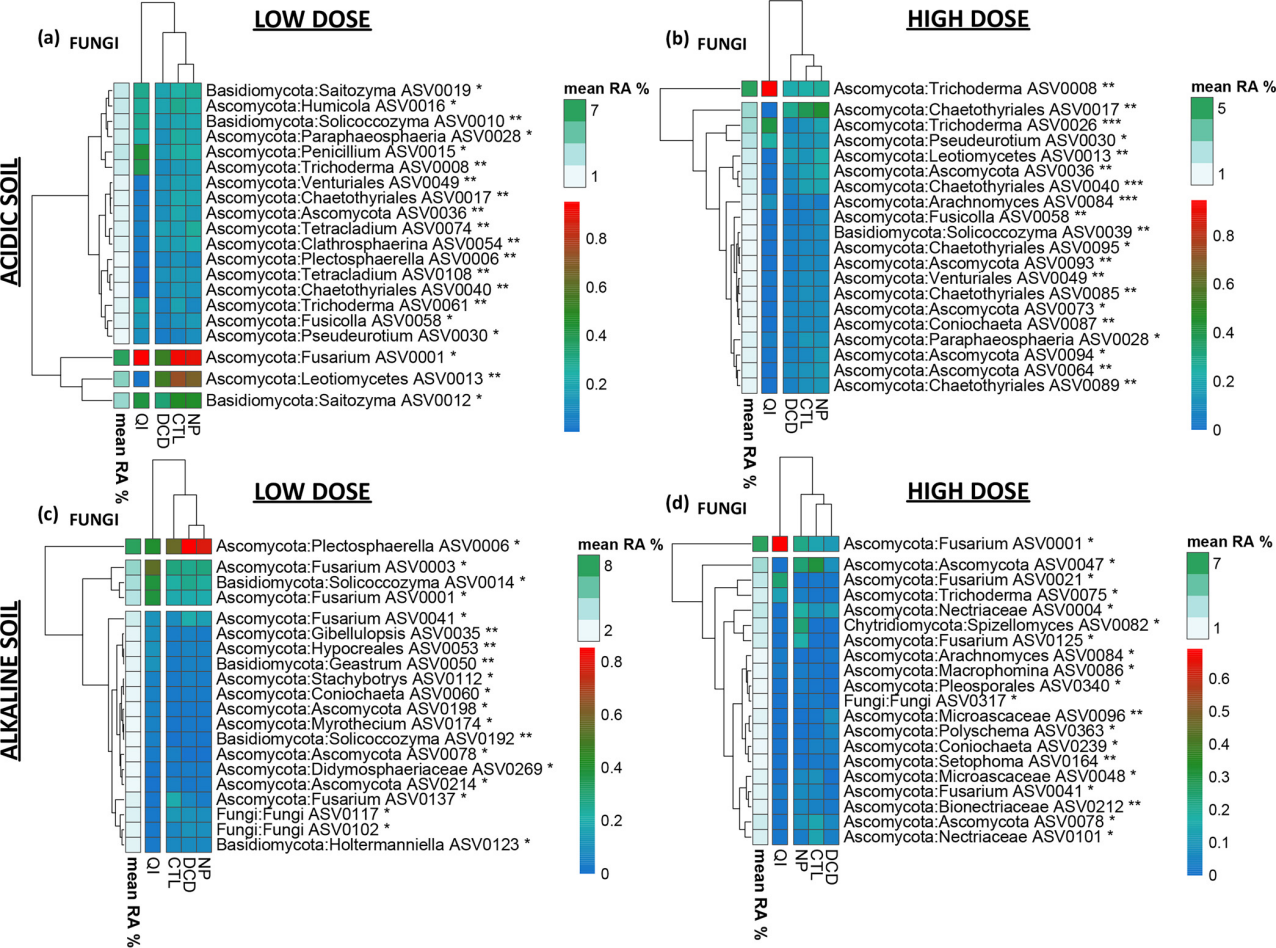
Heatmaps of differentially abundant fungal ASVs (Kruskal-Wallis and Wilcoxon rank sum analysis, test significance is indicated in each ASV annotation * *P* < 0.05, ** *P* < 0.01, *** *P* < 0.001), scaled from 0 to 1 (blue to red)) in the acidic (a, b) and alkaline (c, d) soil for samples treated with the low (a, c) or the high (b, d) dose rate of NIs (DCD, QI, NP) or untreated (CTL). The mean relative abundance of each ASV is indicated by the white-to green column and key. Hierarchical clustering was performed for depicting treatment and ASV groupings. Per ASV barplots with the corresponding *P*-values/ÀSV taxonomies/treatment relative abundances are provided in Fig. S13 to S16 in the supplemental material.

We further looked for amplicon sequencing variants (ASVs) (30) that were associated with certain NI treatments. We first looked for universal responses to NIs across soils. *Sphingomonas* ASVs were significantly reduced in the QI-treated samples from both soils (Fig. 5, S9 to S12). We also identified taxa exhibiting soil-specific responses to QI like *Rhodanobacter* (Fig. 5, S9, S10) and *Lysobacter* (Fig. 5, S11, S12) that significantly increased in the QI-treated samples of the acidic and alkaline soil, respectively. Regarding fungi, in both soils ASVs belonging to Fusarium, *Trichoderma,* and *Solicoccozyma* showed a significant increase in their relative abundance upon exposure to QI; conversely ASVs belonging to *Coniochaeta* were significantly reduced in soils treated with QI (Fig. 6, S13 to S16).

**FIG 7:**
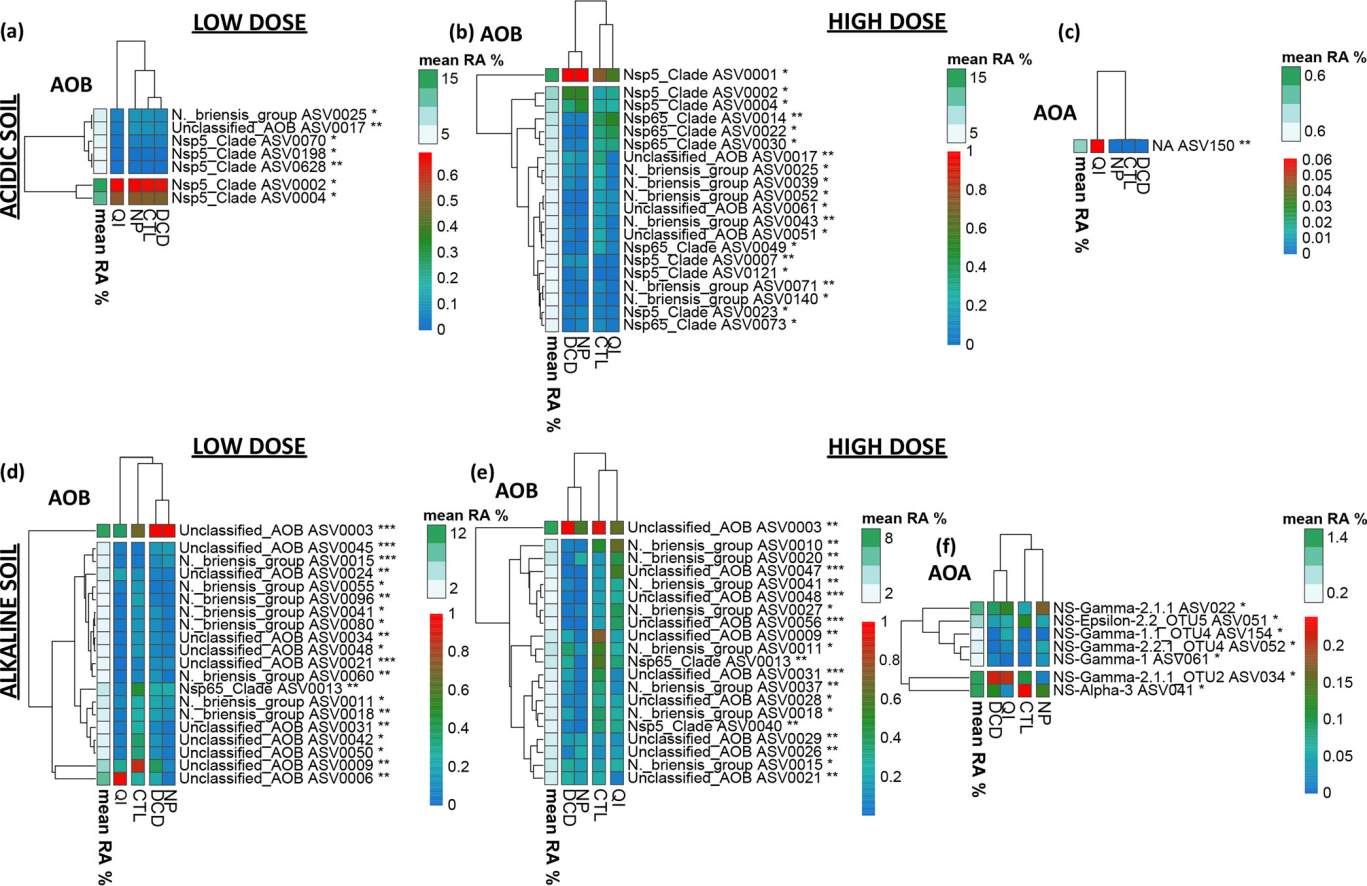
Heatmaps of differentially abundant ASVs of AOB (a, b, d, e) and AOA (c, f) (Kruskal-Wallis and Wilcoxon rank sum analysis, test significance is indicated in each ASV annotation * *P* < 0.05, ** *P* < 0.01, *** *P* < 0.001), scaled from 0 to 1 (blue to red key)) in the acidic (a, b, c) and the alkaline (d, e, f) soil for samples treated with the low (a, d) or the high (b, c, e, f) dose rate of NIs (DCD, QI, NP) or untreated (CTL). The mean relative abundance of each ASV is indicated by the white-to green column and key. Hierarchical clustering was performed for depicting treatment and ASV groupings. Per ASV barplots with the corresponding *P*-values/ÀSV taxonomies/treatment relative abundances are provided in Fig. S17 to S23 in the supplemental material.

For AOM, QI significantly affected the relative abundance of AOB belonging to *Nitrosospira* sp. Nsp5 group in the acidic soil (Fig. 7a, S17), whereas DCD and NP induced a significant reduction on ASVs belonging to *Nitrosospira* sp. Nsp65 and Nitrosospira briensis groups at high dose rates (Fig. 7b, S18). In the alkaline soil, the majority of ASVs significantly affected by NIs belonged to the *N. briensis* group (Fig. 7d and e, S19, S20). Regarding AOA, NP applied in the alkaline soil suppressed ASVs of the *Nitrososphaerales* ε-2.2 clade but favored ASVs belonging to clade γ. In the same soil, ASVs belonging to *Nitrososphaerales* α-3 and ε-2.2. clades decreased with the high dose rate of QI (Fig. 7, S22-23).

## DISCUSSION

### NIs soil dissipation.

QI and NP did not persist in both soils, in line with previous studies (26, 31), unlike DCD, which was the most persistent NI, showing a dose-dependent increase in its persistence (32, 33). The DT_50_ values of DCD in the acidic soil was within the range reported in studies for acidic soils (6.2 to 39 days, at 20–25°C) (14, 33–35), whereas there is a lack of studies on its persistence in alkaline soils, where higher DT_50_ values were recorded in our study. The different persistence of DCD in the two soils might be attributed to their different pH, since the differences in other properties of the two soils known to affect the environmental fate of organic compounds (e.g., organic matter content and soil texture) were small to induce such a distinct effect on the persistence of DCD. pH is a major factor governing the degradation activity of soil microbes (36) but also the polarity of chemicals and hence their sorption and bioavailability (37).

### On-target effects of NIs on the function and diversity of AOM.

An accumulation of NH^+^_4_ was observed only at the high dose rates of the tested NIs and only in the acidic soil. Previous studies looking at the effect of DCD and NP on NH^+^_4_ in various soils have reported contrasting results with accumulation (17, 38, 39) or no increase in NH^+^_4_ retention (17, 40). This is perhaps not surprising considering that, in addition to nitrification, the NH^+^_4_ soil equilibrium is affected by numerous other soil-specific processes, including sorption and NH_3_ volatilization, both of which can be greater in alkaline soils (41). A clear inhibitory effect by all NIs was evident when potential nitrification and NO^−^_3_ levels were considered, and it was more consistent in the alkaline soil. Comprehensive mechanistic studies with stable isotope labeling are required to elucidate the effects of NIs on gross N transformation rates and to provide best insights into N processes other than nitrification (42).

QI reduced the abundance and the transcriptional activity of AOA and AOB in both soils, although effects on the transcriptional activity of AOB were weaker. Earlier studies by Papadopoulou et al. (26) showed a clear and persistent inhibition of the transcription of *amoA* genes of both AOA and AOB by QI applied at rate of 16.1 mg kg^−1^ d.w. soil (i.e., approximately half of the high QI dose in the current study) in an alkaline soil (pH 7.9). The weaker effect of QI on AOB in the current study is probably a function of the lower dose rate of QI used in this study (10 mg kg^−1^ d.w. soil) compared to our earlier study and reflects the lower intrinsic sensitivity of AOB to QI compared to AOA (27).

The impact of DCD on the abundance and activity of the different AOM groups varied in the two studied soils. In the acidic soil DCD showed a more consistent inhibitory effect on AOA, compared to the alkaline soil where DCD was more inhibitory on AOB. At transcriptional level DCD showed again a soil-dependent effect on AOA and AOB. In the acidic soil, it reduced the transcriptional activity of AOA at both dose rates, compared to the alkaline soil, where DCD had a more consistent effect on AOA than on AOB transcripts. Most studies to date have investigated the effects of DCD on the abundance of *amoA* genes but not on the *amoA* transcripts, an indicator of AOM ammonia oxidation activity. These studies have shown that DCD significantly decreased the abundance of AOB, while ΑΟΑ showed no or little response (19, 43). The few studies that have reported a significant inhibition of AOA by DCD (44, 45) were all conducted in acidic soils (pH 4.2 to 5.4), often dominated by AOA. Robinson et al. (12) investigated the effect of DCD on AOB and AOA abundance in a field site where soil pH was maintained at native (pH 6.0) or adjusted to alkaline or acidic levels. They found that DCD inhibited AOB at alkaline and native pH and AOA in acidic pH and concluded that DCD could effectively inhibit AOB or AOA under soil conditions favoring their growth. Our data concur with these findings: in the acidic soil, where AOA prevail functionally and numerically, DCD inhibits AOA more effectively, whereas in the alkaline soil, where AOB are numerically dominant, DCD reduces AOB abundance more effectively than AOA.

NP showed a similar inhibitory pattern with DCD in the two soils. It reduced more effectively the abundance of AOA and AOB in the acidic and alkaline soil, respectively. When looking for effects at *amoA* transcript levels, NP strongly inhibited the expression of AOB and AOA *amoA* gene in the alkaline soil, as opposed to the acidic soil, where the transcriptional activity of the functionally dominant AOA was only temporarily affected by NP. Only a few studies have examined the effect of NP on AOA and AOB dynamics, with contrasting results. In studies performed in acidic soils where AOA functionally and numerically prevailed, NP applied at the recommended dose rates reduced the abundance of AOA but had no effect on AOB (18, 46), while others reported no effect of NP on AOB in soils numerically and functionally dominated by AOB (38). Recently, Hayden et al. (24) reported an increase in AOA abundance in response to NP application in a range of soils, attributed to reduced competition by AOB, whose population was reduced by NP.

Besides their impact on the functional activity of AOM, all NIs induced significant effects in the composition of AOB, unlike AOA, whose community was marginally affected only by the high dose rates of NP and QI. Previous studies (43, 47) suggested a selective effect of DCD on the diversity of AOB, while AOA were not affected, in line with our findings. Recently Schmidt et al. (23) showed that NP, when applied at rates similar to our high dose rate, induced a significant shift on the AOA soil community, in accord with our results. Our study provides first evidence for the effects of NP and QI on the diversity of AOB and AOA, indicating a greater impact of both compounds on AOB compared to AOA, even in soils numerically and functionally dominated by AOA. When looking for specific AOB and AOA that were responsive to the tested NIs, a clear phylogenetic-based inhibition trend was not evident. However, we should note that the sets of primers used in our study may not cover the full range of the AOA harboring *amoA* gene (48, 49).

### Off-target effects of NIs on the abundance and diversity of microbial groups beyond AOM.

Consistent effects on NOB, were observed only in the alkaline soil, where all NIs suppressed *Nitrobacter* at both dose rates, unlike *Nitrospira,* which were inhibited only by the high dose rates of DCD and QI. Previous studies looking at the effect of DCD on NOB in various soils (pH 5.2 to 7.2) reported contrasting results leading to either a decrease (21) or no effect on the abundance of *Nitrobacter* and *Nitrospira* (19, 50). We suggest that the higher inhibitory effect of NIs on *Nitrobacter* over *Nitrospira* NOB most probably reflects the stronger inhibitory activity of the NIs on ammonia oxidation in the alkaline versus the acidic soil. This has resulted in lower substrate (NO^−^_2_-N) availability in the former, a condition expected to favor *Nitrospira*, characterized as k-strategists thriving in soils with nitrite limitations, over *Nitrobacter* which are superior competitors at high resources availability (51). Our hypothesis fits well with the theory that nonrandom associations exist between different AOM and NOB lineages that, along with edaphic factors, shape field-scale spatial patterns of nitrifying communities (52). Still a higher intrinsic tolerance of *Nitrospira* over *Nitrobacter* to NIs cannot be excluded, although data from relevant *in vitro* assays are only available for *Nitrobacter* (27).

The NIs tested did not induce significant alterations in the composition of nontarget prokaryotes and fungal communities, with QI being the sole exception. Little is known regarding the effects of NIs on the overall microbial diversity in soil, with previous studies typically using lower resolution methods that are not directly comparable to our findings (21, 26). Recent studies, using amplicon sequencing, showed that DCD, applied at a dose similar to the low dose rate used in our study, did not alter the composition of the soil bacterial community (50, 53), in line with our findings. We further examined which bacteria and fungi were benefited or suppressed by QI. We noted a soil independent negative effect of QI on *Sphingomonas*. Bacteria of this genus possess multifaceted functions ranging from the biodegradation of environmental contaminants to the production of beneficial phytohormones (54). In contrast, QI stimulated *Lysobacter,* which are considered to be facultative predators (55) or biocontrol microbes, producing secondary metabolites involved in the suppression of plant pathogens (56), pointing to potential impacts of QI on microbial predator-prey interactions in the soil food web. Beyond bacteria, QI favored fungi belonging to Fusarium, *Trichoderma,* and *Solicoccozyma* encompassing plant pathogens (e.g., Fusarium) (57), plant growth promoting agents (e.g., *Trichoderma*, Fusarium) (58, 59), and soil lignocellulose decomposers (e.g., *Solicoccozyma*) (60). These effects could be attributed either (i) to the direct toxicity of QI on target and off-target soil microorganisms that have reciprocal and cascading effects on the proliferation of other soil microorganisms (release from competition, cellular content release as nutrient sources for copiotrophs) or (ii) to the use of QI as a growth substrate favoring the proliferation of the fraction of the soil microbiota being capable of catabolizing and using QI as an energy source. Further studies using shotgun metagenomic and metatranscriptomic approaches are needed to provide insights into the causality of the effects of QI on nontarget soil bacteria and fungi and identify how these diversity changes translate into effects on agricultural soil ecosystem functioning.

### Conclusions.

Using a combination of molecular, biochemical, and analytical tools we provide a comprehensive assessment of the on-target activity (AOM) and off-target effects (NOB, bacteria, fungi) of QI, a new potent NI compound, and two currently used in agriculture NIs, DCD, and NP, on the soil microbiota of two soils mostly varying in pH known to affect microbial community structure, AOM niche differentiation, and chemicals persistence. Our study (i) highlights QI as an alternative, highly efficient NI against AOA, showing, however, significant effects on off-target soil microbiota, within and beyond N cycling, that might reciprocate broader effects on soil ecosystem functioning; (ii) verifies the different sensitivity of the distinct AOM groups and phylotypes to the different NIs that should be considered along with soil microbial dynamics and community composition data for the design of novel, more efficient NI strategies; and (iii) provides first insights into the potential impact of NIs on the wider soil microbial community, beyond the target microbial groups.

## MATERIALS AND METHODS

### Soils and chemicals.

Two arable loamy soils, one acidic vegetable (pH 5.4) and one alkaline (pH 7.9) orchard soil, characterized as Cambisols, were collected in 2019 from two field sites in Thessaly, Greece (61). The two soils were selected to confer very similar physicochemical properties and texture but largely differed in pH (Table 1). Topsoil samples were obtained from 5 selected points (0 to 10 cm depth), according to ISO (62, 63). For each soil, individual samples were mixed thoroughly to provide a single bulk sample (2 kg). After collection, the soils were partially air dried and sieved to pass through a 2 mm mesh sieve. For the preparation of Nis, working solutions for soil treatment and analytical purposes, analytical standards of DCD (99% purity), and NP (≥98%), purchased from Sigma-Aldrich (Germany), were used. The oxidation derivative of ethoxyquin QI was synthesized as described by Thorisson et al. (64).

### Experimental setup.

Each bulk soil was treated with 80 mL of 0.42 M urea (corresponding to 0.47 g N kg^−1^ d.w. soil) to stimulate nitrification and left to equilibrate for 5 days at room temperature, to ensure the ammonification of urea residues in both soils. The final concentration of urea was expected to result in NH_4_^+^-N levels, which have been demonstrated to support the growth of both AOA and AOB (65). Each bulk soil sample was then divided into two sets of four subsamples (0.5 kg each). The first set of subsamples was treated with aqueous suspensions (DCD) or acetonitrile solutions (QI and NP) of NIs, resulting in nominal concentrations in soil of 15.4, 10, and 0.86 mg kg^−1^ d.w. soil of DCD, QI, and NP, respectively. These dose rates were recommended for agricultural use for DCD and NP, assuming that (i) NIs are distributed through the top 5 cm of the soil profile of a field site of 0.1 ha and (ii) soil bulk density was equal to 1.3 g cm^−3^. As QI has no established recommended dose for use as an NI, the nominal concentration of 10 mg kg^−1^ d.w. soil was used, based on previous observations from soil microcosms and liquid batch culture assays (26, 27). A subsample from the first set of each soil was treated with an equivalent amount of acetonitrile and water to serve as an untreated control. DCD-amended samples were also treated with 5 mL acetonitrile to ensure that all treatments received the same volume of water and acetonitrile. Control samples used in the present study were the same as those used in a previous study of our group (61) that was running in parallel, following a similar experimental setup. The second set of subsamples was treated in the same way with the three NIs resulting in concentrations of 200, 30, and 5 mg kg^−1^ d.w. soil for DCD, QI, and NP, respectively. These high dose rates were selected based on the concentrations previously found to suppress the activity of both AOB and AOA strains in liquid culture assays (27). Soil samples were left to equilibrate for 1 h, and sterile water was added to adjust moisture to 40% of the water holding capacity. Soil samples were then split into ~30-g portions and placed in aerated plastic bags, which were incubated in the dark at 20°C for 35 days. Triplicate samples of each treatment were removed from the incubator at 3, 7, 14, 21, and 35 days and stored at –20°C for NI dissipation measurements or at –80°C for molecular analyses. Fresh soil samples were used for NH^+^_4_-N, NO^−^_3_-N, and potential nitrification measurements. The moisture content of all soil samples was kept constant with regular addition of deionized water.

### Analysis of NI residues in soil samples.

DCD residues were extracted from soil according to Kim et al. (66) with slight modifications. Briefly, 5 g of soil was mixed with 10 mL dH_2_O in 50 mL tubes and shaken on a horizontal shaker for 1 h, at 250 rpm, at room temperature. The tubes were centrifuged (7232 × *g*, 5 min), and 2 mL of the soil extract were acidified with 0.2 mL of 0.66 M H_2_SO_4_ and were left to settle for 30 min before centrifugation (16200 × *g*, 10 min). For the extraction of NP, 5 g of soil was mixed with 10 mL acetonitrile. Samples were shaken for 90 min, at 250 rpm, sonicated for 30 min, and centrifuged at 7232 × *g*, for 5 min. Residues of QI were extracted from soil as described by Papadopoulou et al. (26). Before analysis, extracts were passed through 0.45 μm nylon syringe filters. Analysis was performed in a Shimadzu LC-20AD HPLC-PDA system and a Shimadzu GVP-ODS (4.6 mm × 10 mm) precolumn, connected to a RP Shimadzu VP-ODS (4.6 mm × 150 mm, 5 μm) column as described by Papadopoulou et al. (27).

### Analysis of inorganic nitrogen.

To determine NH^+^_4_-N and NO^−^_3_ -N levels in soil samples, soils (2 g) were extracted with 20 mL of 1 M KCl, shaken on a horizontal shaker, at 300 rpm, at room temperature for 30 min, and filtered gravimetrically through Whatman filter paper. NH^+^_4_-N and NO^−^_3_ -N were determined in KCl extracts using a modified indophenol method based on the classical Berthelot reaction (67), and on the VCl_3_/Griess method (68), respectively. Potential nitrification was determined with the method of Kandeler (69).

### Nucleic acid extraction.

RNA was extracted from 2 g of soil using the RNeasy PowerSoil Total RNA isolation kit (Qiagen, Germany) with DNA co-eluted using the RNeasy PowerSoil DNA elution accessory kit (Qiagen, Germany). DNA and RNA purity was determined spectrophotometrically with integrity (shearing and degradation) checked by agarose gel electrophoresis. Nucleic acids were quantified using a Qubit fluorometer (Invitrogen).

### qPCR analysis of AOB, AOA, Comammox bacteria, NOB, total bacteria, fungi, and Thaumarcheota.

The effect of NIs on the abundance of these microbial groups was assessed in samples collected at 14- and 35-days post application. Primers and thermocycling conditions for each target gene are presented in Table S5. qPCR amplification efficiencies were between 82 and 110%, with R^2^ values of ≥ 0.993. Standard curves were obtained using serial dilutions of plasmid vectors containing amplicons of nearly full-length target genes.

### RT-qPCR analysis of AOB and AOA *amoA* gene transcripts.

The effect of NIs on the transcriptional activity of AOB and AOA was further tested via reverse transcription-qPCR (RT-qPCR) of *amoA* gene transcripts. Soil RNA (~300 ng) was treated with DNase I (1 U/μL) (amplification grade; Invitrogen), reverse transcribed, using 200 U Superscript II (Invitrogen) and a 0.2 μM amoA2R and Arch-amoAR primers for AOB and AOA, respectively, according to the manufacturer’s instructions. Amplification of cDNAs for both AOB and AOA was performed following the same procedure as for DNA.

### Amplicon sequencing and bioinformatic analysis.

Microbial diversity analysis for total bacteria, fungi, AOB, and AOA populations were performed on samples of all treatments collected at days 14 and 35, via multiplex amplicon sequencing. A detailed description of the multiplexing method can be found elsewhere (70). Sequencing was carried out at an Illumina HiSeq 2500 sequencer at the Brigham Young University sequencing facilities (Provo, UT, USA), generating 250 bp paired-end reads (bacteria, fungi, AOA) and an Illumina MiSeq sequencer at the Macrogen facilities (Seoul, South Korea), generating 300 bp paired-end reads (AOB). Primers and thermal cycling conditions are shown in Table S6, while primer-indexing sequences are given in Table S7. The read-pairs were assembled and demultiplexed with Flexbar, version 3.0.3 (71). Sequence quality control, generation of ASVs matrices, and sequence classification were performed with dada2 version 1.18.0 (72) using R v3.3.2, software (73) as described in (74). Bacterial 16S rRNA gene amplicon sequence annotations were obtained through assembly of paired reads and subsequently compared with the Silva database v138 (75). Fungal ITS amplicon sequences were classified using UNITE general fasta release version 8.2 (76). AOB and AOA *amoA* amplicons were compared with the databases of Abell et al. (29) and Alves et al. (77), respectively, for classifying taxonomically the generated ASVs. Unclassified AOB *amoA* sequences were subjected to blastx against the nr database (78) and those that were assigned as unclassified or untargeted products were excluded from further analysis.

### Data analysis.

The SFO kinetic model provided adequate fit to the data, and it was used to calculate soil dissipation kinetics of the NIs. The SFO kinetic model is based on the assumption that the change in a given NI concentration with time (d*C*/d*t*) is directly proportional to the actual concentration of the NI at this time (28). The goodness of fit was assessed using a χ^2^ test (<15%, for an α of 0.05), visual inspection, and the distribution of residuals. Data from NH^+^_4_-N, NO^−^_3_-N, potential nitrification, qPCR, and RT-qPCR measurements were subjected to two-way-ANOVA, where treatment and time constituted the two main factors. In case significant interactions between these two factors were observed, significant differences between treatments within each time point were determined via Tukey’s *post hoc* test.

The ASV matrices of bacteria, fungi, AOA, and AOB were used to assess the impact of NIs on the α- and β-diversity values. α-diversity indices used included the Shannon index (79), the Inverse Simpson index (80), observed richness (S), and the Pielou’s evenness index (81). Microbial diversity coverage was assessed through rarefaction curves (82). ANOVA and a Tukey’s *post hoc* analysis or their nonparametric equivalents (Kruskal-Wallis test followed by the Wilcoxon rank-sum test) in case the ANOVA conditions were not met, were implemented for assessing statistically significant differences of each a-diversity index between the treatments. This was performed using the Agricolae v1.3.3 package (83) implemented in R. β-diversity analysis was performed using multivariate and canonical multivariate analysis tests. Detrended correspondence analysis (DCA) on Hellinger-transformed matrices (84) was used to assess the type of ASV responses to the environmental gradients as denoted by the first axis length (unimodal for values higher than 2.5 standard deviations or linear for lower values). Then, CCA or RDA was preferred depending on the first axis value: CCA if higher and RDA if lower than 2.5 standard deviations (85). The PERMANOVA (86), accompanying CCA or RDA, was performed using the pairwise Adonis, v0.0.1 package (87) with the default Bray-Curtis dissimilarity estimation. The nonparametric Kruskal-Wallis test was used for identifying differentially abundant ASVs among the different treatments to assess potential effect at an ASV level (88). All statistical analyses were performed with R.

### Data availability.

The data were submitted to the NCBI Sequence Read Archive with bioproject accession number PRJNA733873.
