# Supplementary material for: The Effects of Quinone Imine, a New Potent Nitrification Inhibitor, Dicyandiamide, and Nitrapyrin on Target and Off-Target Soil Microbiota
Source: Microbiol Spectr. 2022 Jul 20;10(4):e02403-21. doi: 10.1128/spectrum.02403-21 (PMC9431271; doi:10.1128/spectrum.02403-21)
Supplement: Supplemental file 1 — Supplemental material. Download spectrum.02403-21-s0001.pdf, PDF file, 5.3 MB [file spectrum.02403-21-s0001.pdf]

Supplemental material

The effects of quinone imine, a new potent nitrification inhibitor, dicyandiamide and nitrapyrin on target and off-target soil microbiota

Evangelia S. Papadopoulou[1,2*], Eleftheria Bachtsevani[1], Christina V. Papazlatani[1], Constantina Rousidou[1], Antonios Brouziotis[1], Eleni Lampronikou[1], Myrto Tsiknia[3], Sotirios Vasileiadis[1], Ioannis Ipsilantis[4], Urania Menkissoglu-Spiroudi[5], Constantinos Ehaliotis[3], Laurent Philippot[6], Graeme W. Nicol[7], Dimitrios G. Karpouzas[1]

[1]*Laboratory of Plant and Environmental Biotechnology, Department of Biochemistry and Biotechnology, University of Thessaly, Larissa, Greece*

[2]*Laboratory of Environmental Microbiology, Department of Environmental Sciences, University of Thessaly, Larissa, Greece*

[3]*Laboratory of Soils and Agricultural Chemistry, Agricultural University of Athens, Athens, Greece*

[4]*Laboratory of Soil Sciences, School of Agriculture, Forestry and Environment, Faculty of Agriculture, Aristotle University of Thessaloniki, Thessaloniki, Greece*

[5] *Pesticide Science Laboratory, School of Agriculture, Forestry and Environment, Faculty of Agriculture, Aristotle University of Thessaloniki, Thessaloniki, Greece*

[6] *Université Bourgogne Franche-Comté, INRAE, AgroSup Dijon, Agroécologie, 21000 Dijon, France*

[7] *Environmental Microbial Genomics Group, Laboratoire Ampère, École Centrale de Lyon, CNRS UMR 5005, Université de Lyon, Ecully 69134, France*

[+] Corresponding author

Dr. Evangelia S. Papadopoulou

Tel. +30-2410-565232, Fax. +30-2410-565290, Email. evapapadopoulou@.uth.gr

Supplemental tables

TABLE S1 Ratio of AOA *amoA* to thaumarcheotal 16S rRNA gene copies calculated in the acidic and the alkaline soil treated with the low and the high dose rates of dicyandiamide (DCD), nitrapyrin (NP), and quinone imine (QI). Asterisks indicate significant differences between control and NIs low or NIs high dose rates ($p < 0.05$) within each soil and time point.

SOIL	DOSE	TIME (days)	TREATMENT	*amoA*: 16S rRNA
ACIDIC	low	14	CTL	3.1±0.6
			DCD	4.5±0.9
			NP	26.5±7.0*
			QI	15.2±2.6
		35	CTL	0.7±0.1
			DCD	0.9±0.1
			NP	2.0±1.2
			QI	12.7±6.4
	high	14	CTL	4.1±1.1
			DCD	13.4±2.1
			NP	19.7±5.8
			QI	126.5±34.5*
		35	CTL	12.2±3.1
			DCD	11.6±3.9
			NP	9.0±4.0
			QI	6.1±2.2
ALKALINE	low	14	CTL	0.10±0.06
			DCD	0.13±0.03
			NP	0.04±0.03
			QI	0.26±0,13
		35	CTL	0.09±0.01
			DCD	0.14±0.01
			NP	0.06±0.03
			QI	0.11±0.03
	high	14	CTL	1.2±0.7
			DCD	3.7±3.0
			NP	3.1±0.4
			QI	7.6±1.1
		35	CTL	48.7±32.9
			DCD	0.05±0.04
			NP	21.1±11.5
			QI	2.5±2.1

TABLE S2 Per sample sequence numbers obtained and passing quality control screening.

					BACTERIA		FUNGI		AOB		AOA	
**SOIL**	**DOSE**	**TIME**	**TREATMENT**	**BIOLOGICAL REPLICATE**	**No. of raw sequences**	**Post quality screening**	**No. of raw sequences**	**Post quality screening**	**No. of raw sequences**	**Post quality screening**	**No. of raw sequences**	**Post quality screening**
ACIDIC	HIGH	T14	CTL	1	166573	111248	27536	18595	37894	17150	5264	1882
				2	80485	52456	-	-	2297	1397	3866	1829
				3	74113	50441	23007	16850	25511	14056	3591	1666
			DCD	1	23950	16207	24884	17202	25937	15639	2324	1333
				2	28874	17978	20141	13814	23054	15346	2605	1476
				3	31278	20577	20433	12727	32041	21114	1922	1120
			NP	1	22792	14483	20009	13670	25285	14192	3311	1503
				2	31653	21188	17161	11806	28861	15245	8307	2851
				3	108271	74671	3049	1995	23469	12108	4388	1921
			QI	1	51090	37811	23198	19326	32131	7298	3535	1875
				2	67502	51055	26298	21653	30565	9452	2130	1288
				3	32826	23633	21878	17901	27811	8989	2409	1576
		T35	CTL	1	36621	23949	24641	17795	22324	13159	2215	1271
				2	34049	22632	25419	19000	-	-	2281	999
				3	37304	24971	23206	17886	29984	14815	2578	1275
			DCD	1	61882	42233	17512	9703	19527	10194	2286	762
				2	72462	50498	16546	8809	30379	17302	1689	719

				3	38500	26194	42	4	23203	12910	398	147
			NP	1	151332	106106	47044	30449	27012	19052	809	299
				2	18700	12223	26458	19406	26794	17815	1403	509
				3	42034	29334	15548	10796	22894	12361	2379	975
			QI	1	39052	23064	12839	7702	35406	14236	815	445
				2	38631	25541	12126	6463	33150	15215	2294	1315
				3	69777	48232	16030	10852	50224	21182	2612	1557
ACIDIC	LOW	T14	CTL	1	62368	39406	23359	17916	24468	12290	5230	3672
				2	36440	22285	22724	17389	1984	1162	4464	3309
				3	40684	24614	23100	17878	22390	10548	3058	2103
			DCD	1	21014	12084	22789	17618	20291	10392	2463	1641
				2	13789	7589	16485	12755	21177	10170	2487	1659
				3	21428	12312	18934	14371	17535	9169	2481	1704
			NP	1	11970	5958	23718	17479	19232	9039	4535	3173
				2	12999	6404	24516	18714	16957	8413	5910	4264
				3	50728	30665	23950	18172	22820	9642	4153	2958
			QI	1	33257	19611	17785	14406	20860	9724	3859	2782
				2	42751	25923	10618	8143	16747	8183	3130	2240
				3	52470	33071	15620	12330	19470	10173	1462	1059
		T35	CTL	1	53143	34220	9409	6950	21505	13565	2006	1572

				2	9109	4658	39921	35359	30086	21034	5439	4377
				3	33441	20697	14793	11000	24811	17593	4675	3578
			DCD	1	25842	14413	27916	21648	20558	12162	1725	1214
				2	19564	11631	12482	8818	41741	32594	7657	6463
				3	54843	33602	20982	15977	19560	8697	3675	2559
			NP	1	46851	27321	16446	12589	17818	9999	3009	2092
				2	53135	33395	10111	7404	17938	8609	3130	2267
				3	23050	13756	14320	10896	16974	9786	2433	1765
			QI	1	39584	25127	20365	16471	15706	7656	2275	1612
				2	17436	10334	11854	9390	16221	8676	2616	1803
				3	34158	21359	20744	16753	11403	5426	2602	1872
ALKALINE	HIGH	T14	CTL	1	116920	74649	13364	10368	41543	16693	2017	1391
				2	109741	70760	13758	10014	46991	18756	5506	3901
				3	44865	30215	9749	7118	48488	19584	2619	1838
			DCD	1	16037	8074	14397	11478	41799	20078	2139	1681
				2	26257	15713	31570	27552	53087	28903	4892	3428
				3	12235	6160	11068	7882	40721	19563	7044	5552
			NP	1	48881	29623	5997	4571	32394	19280	2866	2153
				2	79584	48770	19747	13135	29234	18324	2698	1961
				3	72974	46907	14094	10035	30174	15690	5428	3889

			QI	1	69417	47730	15177	10469	52819	19876	1810	1324
				2	7386	4508	15783	12232	44866	16893	2975	2171
				3	69262	47270	13649	9346	39013	15873	4923	3764
		T35	CTL	1	34592	22909	14045	9780	35434	13613	3703	2565
				2	13793	8358	12869	9414	65782	28568	2791	1949
				3	4977	2187	14994	11070	35890	13541	4003	2767
			DCD	1	116393	71943	25	3	38556	20941	-	-
				2	58887	36633	2119	1606	31154	19596	1290	986
				3	123498	79523	14426	11205	31560	18596	4027	2942
			NP	1	33064	20914	14781	10628	28722	18134	3232	2467
				2	29609	19706	13411	10633	33549	16700	2690	2010
				3	23722	14531	27288	20497	34141	19447	3889	2898
			QI	1	21841	14604	15919	13605	42193	17012	2003	1565
				2	23688	16745	15606	13347	34813	16772	3808	2837
				3	68361	48736	21640	19029	37487	12526	4355	3210
ALKALINE	LOW	T14	CTL	1	23855	11037	22642	16953	29789	10393	8305	6602
				2	21008	9333	28018	19866	44203	16997	4103	3149
				3	14542	6369	20671	16518	23997	7639	8707	6309
			DCD	1	38091	19832	20812	15569	22190	7517	8845	6955
				2	39323	20923	24347	16970	20176	6853	5882	4644

				3	55821	32206	14536	9244	29144	11597	8631	7051
			NP	1	25367	12164	15735	11564	26888	10032	8375	6873
				2	19373	8996	15818	12184	19966	6989	7880	6136
				3	16955	7195	11107	7942	21716	8919	9040	7336
			QI	1	26430	14309	17839	13284	23650	8323	4877	3630
				2	17465	8436	10663	7236	26806	9671	5360	4156
				3	34015	19037	14412	10415	14343	5751	3769	2932
		T35	CTL	1	30105	15992	10916	7544	23417	7490	6275	5168
				2	59086	33939	10377	8101	23333	7527	3113	2346
				3	42420	23867	9033	6483	21612	6429	6283	4994
			DCD	1	55573	31619	10552	7574	27920	10231	5568	4336
				2	40464	21813	7562	5385	32492	12776	5425	4289
				3	20142	9540	19789	15036	30158	14155	4207	3372
			NP	1	25786	12561	34117	25275	25259	11738	3843	3019
				2	19542	8645	260	127	26750	11828	3906	3061
				3	10419	4182	33436	25335	-	-	3500	2590
			QI	1	14365	6951	26472	19208	29365	12485	6009	4701
				2	24896	14551	22996	17461	30162	12301	7821	6325
				3	50534	29804	22547	16715	28369	13862	7873	6250
			**TOTAL**		**4121366**	**2587629**	**1698079**	**1261836**	**2666120**	**1261391**	**378190**	**267804**

TABLE S3 Total abundance of the ASVs of each AOB taxonomic group per treatment (CTL: Control, DCD: Dicyandiamide, NP: Nitrapyrin, QI: Quinone Imine).

	ACIDIC SOIL *LOW DOSE*				ACIDIC SOIL *HIGH DOSE*			
**Taxonomic group**	**CTL**	**DCD**	**NP**	**QI**	**CTL**	**DCD**	**NP**	**QI**
Nc. mobilis cluster	0 ± 0	0 ± 0	0 ± 0	0 ± 0	0 ± 0	0 ± 0	0 ± 0	0 ± 0
Nitrosomonas oligotropha	0 ± 0	0 ± 0	0 ± 0	0 ± 0	0 ± 0	0 ± 0	0 ± 0	0 ± 0
Nitrosospira briensis cluster	1751.7 ± 602.9	887.7 ± 83.5	615.0 ± 45.5	457.5 ± 93.4	2763.2 ± 584.5	1918.5 ± 213.4	1404.7 ± 181.0	1474.5 ± 454.7
Nitrosospira et rel	210.2 ± 41.8	353.5 ± 186.8	161.7 ± 75.0	188.2 ± 50.7	3695.6 ± 1098.0	1723.3 ± 424.4	1675.2 ± 158.9	5532.5 ± 1831.7
Nitrosospira Unc AOB II	10171.8 ± 2369.6	12260.8 ± 3581.3	8118.3 ± 209.3	7497.0 ± 610.6	2866.0 ± 989.8	9264.8 ± 1085.5	10087.5 ± 1018.0	3281.7 ± 612.6
Unclassified	565.0 ± 114.8	362.0 ± 82.1	353.0 ± 29.6	163.7 ± 20.3	2790.6 ± 665.0	2510.8 ± 248.0	1961.5 ± 176.0	2440.0 ± 719.0
	**ALKALINE SOIL *LOW DOSE***				**ALKALINE SOIL *HIGH DOSE***			
**Taxonomic group**	**CTL**	**DCD**	**NP**	**QI**	**CTL**	**DCD**	**NP**	**QI**
Nc. mobilis cluster	2.8 ± 2.8	0 ± 0	0 ± 0	0.3 ± 0.3	0 ± 0	0 ± 0	0 ± 0	0 ± 0
Nitrosomonas oligotropha	0 ± 0	0 ± 0	1.6 ± 1.6	0 ± 0	0 ± 0	0 ± 0	0 ± 0	0 ± 0
Nitrosospira briensis cluster	3019.3 ± 290.6	4158.3 ± 322.0	3172.6 ± 295.6	2034.8 ± 466.8	6252.8 ± 547.9	7029.0 ± 684.5	7089.7 ± 904.7	6292.2 ± 436.0
Nitrosospira et rel	485.8 ± 33.1	509.2 ± 127.5	683.6 ± 140.1	99.7 ± 32.0	2029.8 ± 1003.6	1346.2 ± 141.8	284.7 ± 62.2	746.7 ± 129.3
Nitrosospira Unc AOB II	1498.7 ± 1297.7	124.3 ± 43.5	193.0 ± 41.8	28.0 ± 16.4	1486.3 ± 530.8	657.7 ± 59.9	336.7 ± 57.7	1142.7 ± 189.7
Unclassified	4405.8 ± 270.8	5729.7 ± 729.5	5850.4 ± 552.4	8236.0 ± 1583.4	8690.2 ± 732.2	12246.7 ± 864.1	10218.2 ± 1118.2	8310.5 ± 648.2

TABLE S4 Total abundance of the ASVs of each AOA taxonomic group per treatment (CTL: Control, DCD: Dicyandiamide, NP: Nitrapyrin, QI: Quinone Imine).

	ACIDIC SOIL *LOW DOSE*				ACIDIC SOIL *HIGH DOSE*			
**Taxonomic group**	**CTL**	**DCD**	**NP**	**QI**	**CTL**	**DCD**	**NP**	**QI**
NS[a]	27.0 ± 4.8	19.7 ± 15.4	31.8 ± 7.5	12.2 ± 6.1	16.5 ± 1.7	4.3± 2.9	5.3 ± 3.8	10.2 ± 4.0
NS-Alpha-3	0 ± 0	0 ± 0	0 ± 0	0 ± 0	0 ± 0	0 ± 0	0 ± 0	0 ± 0
NS-Alpha-3.1	2.8 ± 2.0	0 ± 0	0 ± 0	0 ± 0	0.7 ± 0.7	0 ± 0	0 ± 0	0 ± 0
NS-Alpha-3.2	1.7 ± 1.7	0 ± 0	2 ± 2	0 ± 0	0 ± 0	0 ± 0	0 ± 0	0 ± 0
NS-Beta-1. Incertae sedis	0 ± 0	0 ± 0	0 ± 0	0 ± 0	0 ± 0	0 ± 0	0 ± 0	0 ± 0
NS-Beta-1 OTU12	0 ± 0	0 ± 0	0 ± 0	0 ± 0	0.7 ± 0.7	0 ± 0	0 ± 0	0 ± 0
NS-Beta-2.2	19.8 ± 8.3	12.8 ± 5.0	25.0 ± 4.1	12.3 ± 2.6	0.8 ± 0.8	0 ± 0	0 ± 0	2.2 ± 1.4
NS-Delta-1.1	0 ± 0	0 ± 0	6.2 ± 3.9	1.8 ± 1.8	0 ± 0	0 ± 0	0 ± 0	0 ± 0
NS-Delta-1.2	0 ± 0	1.3 ± 1.3	0 ± 0	1.0 ± 1.0	0 ± 0	0 ± 0	0 ± 0	0 ± 0
NS-Epsilon-2.2	1.3 ± 1.3	0 ± 0	0 ± 0	0 ± 0	1.5 ± 1.5	4 ± 4	1.8 ± 1.8	0.5 ± 0.5
NS-Gamma-1	0 ± 0	0 ± 0	0 ± 0	0 ± 0	0 ± 0	0 ± 0	0 ± 0	0 ± 0
NS-Gamma-1.1	19.3 ± 10.1	0 ± 0	4 ± 4	0 ± 0	0 ± 0	0 ± 0	0 ± 0	0 ± 0
NS-Gamma-1.2	0 ± 0	0 ± 0	0 ± 0	0 ± 0	0 ± 0	0 ± 0	0 ± 0	0 ± 0
NS-Gamma-1. Incertae sedis	2.0 ± 2.0	0 ± 0	0 ± 0	0 ± 0	0.8 ± 0.8	0 ± 0	0 ± 0	0 ± 0
NS-Gamma-2	604.7 ± 112.5	503.0 ± 186.0	676.0 ± 145.1	383.2 ± 35.0	398.8 ± 58.0	287.0 ± 56.0	499.8 ± 160.5	428.7 ± 86.5
NS-Gamma-2.1	24.0 ± 19.7	0 ± 0	0.8 ± 0.5	0 ± 0	0 ± 0	0 ± 0	5.0 ± 4.3	0.3 ± 0.3
NS-Gamma-2.2	2255.5 ± 319.6	1927.7 ± 621.2	1829.0 ± 188.2	1370.0 ± 188.1	1058.3 ± 107.2	627.8 ± 151.5	825.5 ± 223.9	851.8 ± 122.9
NS-Gamma-2.3	121.7 ± 27.1	48.7 ± 15.34	128.5 ± 22.8	69.7 ± 15.2	1.2 ± 1.2	0 ± 0	0.5 ± 0.5	4.7 ± 4.7
NS-Gamma-2_OTU1	0 ± 0	0 ± 0	0 ± 0	0 ± 0	0 ± 0	0 ± 0	0 ± 0	0 ± 0
NS-Zeta	0 ± 0	0 ± 0	0 ± 0	0 ± 0	0 ± 0	0 ± 0	0 ± 0	0 ± 0
NS-Zeta-1.2	0 ± 0	0 ± 0	1.7 ± 1.7	0.8 ± 0.8	0 ± 0	0 ± 0	0 ± 0	0 ± 0
Unclassified	22.0 ± 10.4	26.8 ± 7.01	48.2 ± 18.9	43.7 ± 8.2	7.7 ± 6.3	3.0 ± 3.0	5.0 ± 2.8	44.3 ± 14.8

	ALKALINE SOIL *LOW DOSE*				ALKALINE SOIL *HIGH DOSE*			
**Taxonomic group**	**CTL**	**DCD**	**NP**	**QI**	**CTL**	**DCD**	**NP**	**QI**
NS	0 ± 0	0 ± 0	0 ± 0	0 ± 0	0 ± 0	0 ± 0	0 ± 0	0 ± 0
NS-Alpha-3	137.5 ± 19.9	55.7 ± 6.1	119.8 ± 26.3	90.2 ± 17.0	71.7 ± 21.0	52.2 ± 15.7	49.0 ± 10.5	11.2 ± 5.6
NS-Alpha-3.1	609.3 ± 110.9	840.5 ± 126.6	623.7 ± 105.0	744.3 ± 90.1	153.2 ± 31.2	150.4 ± 50.4	177.8 ± 35.6	137.0 ± 27.6
NS-Alpha-3.2	166.2 ± 38.8	78.3 ± 8.5	76.8 ± 16.9	67.2 ± 8.8	9 .0± 2.2	7.4 ± 3.8	6.2 ± 4.5	6.0 ± 1.7
NS-Beta-1. Incertae sedis	8.5 ± 1.9	10.5 ± 2.3	16.3 ± 4.1	21.0 ± 6.0	2.2 ± 1.1	0.8 ± 0.8	2.7 ± 1.1	1.8 ± 0.9
NS-Beta-1_OTU12	0 ± 0	2.3 ± 2.3	2.5 ± 2.5	1.5 ± 1.5	1.0 ± 1.0	1.4 ± 1.4	0 ± 0	0 ± 0
NS-Beta-2.2	0 ± 0	0 ± 0	0 ± 0	0 ± 0	0 ± 0	0 ± 0	0 ± 0	0 ± 0
NS-Delta-1.1	228.0 ± 35.9	126.2 ± 15.0	135.5 ± 32.2	86.3 ± 5.9	43.8 ± 10.1	26.2 ± 9.1	42.8 ± 8.9	50.0 ± 13.7
NS-Delta-1.2	3.8 ± 2.4	14.5 ± 4.1	13.5 ± 4.9	12.7 ± 6.4	1.3 ± 1.3	1.2 ± 1.2	0.8 ± 0.8	6.5 ± 3.5
NS-Epsilon-2.2	809.5 ± 84.2	608.0 ± 75.4	722.3 ± 126.5	620.3 ± 105.3	670.5 ± 81.3	908.6 ± 244.3	779.8 ± 69.8	804.8 ± 121.1
NS-Gamma-1	294.0 ± 31.6	349.8 ± 39.8	358.3 ± 63.1	291.12 ± 25.1	162.5 ± 20.4	191.0 ± 42.2	204 .0± 24.5	215.0 ± 37.6
NS-Gamma-1.1	256.2 ± 53.7	315.2 ± 38.1	370.7 ± 97.6	313.7 ± 53.0	254.7 ± 34.7	358.0 ± 93.7	297.3 ± 35.3	224.2 ± 33.2
NS-Gamma-1.2	146.8 ± 16.7	147.7 ± 9.8	134.2 ± 37.5	144.5 ± 18.4	69.2 ± 12.4	31.4 ± 6.9	59.0 ± 15.3	67.3 ± 12.6
NS-Gamma-1. Incertae sedis	253.8 ± 34.5	344.3 ± 32.8	309.5 ± 71.23	285.8 ± 41.7	87.2 ± 14.1	169.0 ± 46.4	114.7 ± 14.6	126.2 ± 35.1
NS-Gamma-2	0 ± 0	0 ± 0	0 ± 0	0 ± 0	0 ± 0	0 ± 0	0 ± 0	0.8 ± 0.8
NS-Gamma-2.1	1623.0 ± 332.6	2001.0 ± 305.9	1704.3 ± 273.8	1764.7 ± 225.4	828.2 ± 161.9	993.0 ± 294.1	792.2 ± 105.4	800 .0± 134.9
NS-Gamma-2.2	214.3 ± 49.2	207.2 ± 17.7	232.8 ± 49.3	206.5 ± 37.9	45.2 ± 13.0	27.2 ± 14.9	35.5 ± 7.7	26.7 ± 4.4
NS-Gamma-2.3	0 ± 0	0 ± 0	0 ± 0	0 ± 0	0 ± 0	0 ± 0	0 ± 0	0 ± 0
NS-Gamma-2 OTU1	0.5 ± 0.5	0 ± 0	0 ± 0	0 ± 0	0 ± 0	0 ± 0	0.3 ± 0.3	0 ± 0
NS-Zeta	0.7 ± 0.7	0 ± 0	0.5 ± 0.5	1.2 ± 1.2	0 ± 0	0 ± 0	0 ± 0	0 ± 0
NS-Zeta-1.2	0 ± 0	0 ± 0	0 ± 0	0 ± 0	0 ± 0	0 ± 0	0 ± 0	0 ± 0
Unclassified	9.2 ± 2.7	6.7 ± 2.6	15.0 ± 6.1	14.7 ± 6.1	2.3 ± 1.5	0 ± 0	0.8 ± 0.5	1 .0± 0.5

[a] *Nitrososphaerales*

TABLE S5 The observed richness and diversity indices (Shannon, Inverse Simpson and Pielou's evenness) calculated for the different microbial groups in the acidic and the alkaline soil treated with the low and the high dose rates of dicyandiamide (DCD), nitrapyrin (NP), and quinone imine (QI). Asterisks indicate significant differences between control and NIs low or NIs high dose rates ($p < 0.05$) within each soil and microbial group tested.

SOIL	DOSE	TREATMENT	OBSERVED	DIVERSITY SHANNON	DIVERSITY INVERSE_SIMPSON	EVENNESS PIELOU
			**BACTERIA**			
ACIDIC	LOW	CTL	618.5 ± 111	6.05 ± 0.26	387.6± 67.2	0.96 ± 0.004
		DCD	396.3 ± 64.6	5.66 ± 0.15	237.8 ± 41.1	0.95 ± 0.002
		NP	459.0 ± 113	5.70 ± 0.26	278.5 ± 67.6	0.96 ± 0.003
		QI	583.3 ± 71.9	6.09 ± 0.14	374.5 ± 42.6	0.96 ± 0.001
	HIGH	CTL	1147.7 ± 270	6.52 ± 0.16	363.1 ± 15.4	0.94 ± 0.005
		DCD	786.7 ± 135	6.14 ± 0.16	258.8 ± 39.6	0.93 ± 0.003
		NP	1019.2 ± 305	6.25 ± 0.25	279.6 ± 46.9	0.93 ± 0.006
		QI	761.3 ± 79.3	6.14 ± 0.10	312.5 ± 40.3	0.93 ± 0.01
ALKALINE	LOW	CTL	421.5 ± 102	5.65 ± 0.24	272.0 ± 67.6	0.96 ± 0.003
		DCD	600.5 ± 80	6.1 ± 0.16	387.7± 50.7	0.96 ± 0.002
		NP	257.8 ± 37	5.25 ± 0.16	169.4 ± 26	0.95 ± 0.003
		QI	408.3 ± 73.5	5.68 ± 0.16	255.0 ± 38.7	0.96 ± 0.001
	HIGH	CTL	1039.2 ± 330	6.24 ± 0.47	521.0 ± 158	0.96 ± 0.003
		DCD	994.8 ± 331	6.26 ± 0.41	520.1 ± 149	0.96 ± 0.002
		NP	927.7 ± 152	6.49 ± 0.17	524.0 ± 81.4	0.96± 0.003
		QI	633.3 ± 164	5.53 ± 0.29	119.4 ± 17.1*	0.89 ± 0.005*
			**FUNGI**			
ACIDIC	LOW	CTL	262.8 ± 20.4	4.69± 0.076	62.54 ± 4.99	0.84 ± 0.007
		DCD	258.3 ± 14	4.64 ± 0.051	52.75 ± 4.39	0.84 ± 0.007
		NP	250.2± 17.7	4.67 ± 0.064	61.08 ± 6.04	0.85 ± 0.008

		QI	215.8 ± 11.3	4.36 ± 0.061*	40.83 ± 3.06 *	0.81 ± 0.008*
	HIGH	CTL	219.6 ± 6.58	4.41 ± 0.03	40.59 ± 2.64	0.82 ± 0.006
		DCD	139.2 ± 31.9	3.50 ± 0.71*	29.8 ± 7.29	0.83 ± 0.007
		NP	184.2 ± 24.5	4.34 ± 0.084	41.66 ± 2.89	0.84 ± 0.011
		QI	151.5 ± 17.6	3.78 ± 0.061*	21.92 ± 1.5*	0.76 ± 0.015*
ALKALINE	LOW	CTL	190.3 ± 16.1	4.07 ± 0.11	27.79 ± 4.08	0.78 ± 0.02
		DCD	199.0 ± 20.6	4.20± 0.13	33.52 ± 4.36	0.80 ± 0.01
		NP	199.0 ± 39.9	3.94 ± 0.37	28.93 ± 6.24	0.79 ± 0.01
		QI	212.8 ± 15.1	4.12 ± 0.13	29.14 ± 4.84	0.77 ± 0.021
	HIGH	CTL	129.0 ± 21	3.85 ± 0.084	21.17 ± 3.02	0.81 ± 0.03
		DCD	113.3 ± 27.8	2.73 ± 0.61	12.91 ± 7.12	0.68 ± 0.07
		NP	123.3 ± 14.4	3.74 ± 0.14	19.80 ± 3.81	0.78 ± 0.02
		QI	104.7 ± 11.6	3.31 ± 0.32	20.18 ± 8.34	0.71 ± 0.06
**AOB**						
ACIDIC	LOW	CTL	63.83 ± 10.8	2.92 ± 0.14	10.32 ± 0.72	0.74 ± 0.03
		DCD	56.33 ± 3.37	2.84 ± 0.04	9.02 ± 0.33	0.71 ± 0.01
		NP	48 ± 2.27	2.72 ± 0.04	8.40 ± 0.35 *	0.70 ± 0.01
		QI	34 ± 1.81 *	2.47 ± 0.04 *	7.05 ± 0.22 *	0.70 ± 0.01
	HIGH	CTL	85.2 ± 15.9	3.67 ± 0.17	25.23 ± 3.75	0.85 ± 0.03
		DCD	83.67 ± 14.5	3.47 ± 0.14	16.39 ± 1.2 *	0.8 ± 0.01
		NP	72.33 ± 5.78	3.26 ± 0.06	12.28 ± 0.86 *	0.76 ± 0.01 *
		QI	72.5 ± 8.37	3.26 ± 0.13	14.61 ± 2 *	0.77 ± 0.03
ALKALINE	LOW	CTL	111.17 ± 10.5	4.13 ± 0.07	37.60 ± 3.53	0.88 ± 0.01
		DCD	110.5 ± 8.59	4.07 ± 0.04	29.77 ± 1.93	0.87 ± 0.01
		NP	87.8 ± 5.21	3.66 ± 0.07 *	15.35 ± 1.06 *	0.82 ± 0.01 *
		QI	48.83 ± 8.47 *	2.9 ± 0.30 *	12.8 ± 3.76 *	0.75 ± 0.05 *
		CTL	194 ± 13.3	4.62 ± 0.06	61.79 ± 3.66	0.88 ± 0.004
		DCD	199.17 ± 10.7	4.51 ± 0.04	41.45 ± 1.87 *	0.85 ± 0.004 *

	**HIGH**	NP	90.67 ± 15.8 *	3.17 ± 0.31 *	12.3 ± 2.77 *	0.71 ± 0.04 *
		QI	139.33 ± 5.07 *	4.25 ± 0.04 *	42.52 ± 2.86 *	0.86 ± 0.005 *
			**AOA**			
**ACIDIC**	**LOW**	CTL	25.17 ± 3.19	2.36 ± 0.15	7.85 ± 1.34	0.74 ± 0.02
		DCD	18.17 ± 1.7	2.17 ± 0.095	6.71 ± 0.68	0.75 ± 0.02
		NP	27.5 ± 3.49	2.44 ± 0.066	7.85 ± 0.48	0.75 ± 0.02
		QI	21.33 ± 2.06	2.32 ± 0.097	7.49± 0.67	0.76 ± 0.02
	**HIGH**	CTL	13.33 ± 1.28	1.99 ± 0.098	5.83 ± 0.65	0.78 ± 0.03
		DCD	8.5 ± 1.65	1.70 ± 0.23	5.03± 0.80	0.84 ± 0.02
		NP	10.17 ± 2.3	1.60 ± 0.14	3.95 ± 0.36	0.76 ± 0.04
		QI	12.33 ± 1.63	1.73 ± 0.095	4.13 ± 0.31	0.70 ± 0.02
**ALKALINE**	**LOW**	CTL	84.17 ± 9.44	4.08 ± 0.11	45.71 ± 4.16	0.93 ± 0.003
		DCD	90 ± 7.24	4.20 ± 0.071	53.18 ± 2.93	0.94 ± 0.002
		NP	84 ± 11.9	4.07 ± 0.14	46.93 ± 5.43	0.93 ± 0.002
		QI	84.67 ± 7.85	4.14 ± 0.082	49.35± 3.06	0.94 ± 0.002
	**HIGH**	CTL	47.5 ± 5.6	3.48 ± 0.11	25.39 ± 2.48	0.91 ± 0.001
		DCD	50.4 ± 10.2	3.43 ± 0.17	21.03 ± 3.05	0.90 ± 0.01 *
		NP	51.33 ± 5.26	3.47 ± 0.097	21.82 ± 2.5	0.89 ± 0.007 *
		QI	50.17 ± 5.72	3.45 ± 0.11	21.94 ± 1.89	0.89 ± 0.006 *

TABLE S6 Primers, primer sequences, and thermocycling conditions used for q-PCR and amplicon sequencing.

Target gene	Primer name	Primers Sequence	Thermal cycling conditions	Reference
**q-PCR analyses**				
AOB *amoA*	amoA-1F /amoA-2R	5′-GGGGTTTCTACTGGTGGT-3′ 5′-CCCCTCKGSAAAGCCTTCTTC-3’	95ºC for 5, 57ºC for 10s, 72ºC for 30s (40 cycles)	1
AOA *amoA*	Arch-amoAF Arch-amoAR	5'- STAATGGTCTGGCTTAGACG-3′ 5'-GCGGCCATCCATCTGTATGT-3’	95ºC for 5s, 53ºC for 10s, 72ºC for 30s (45cycles)	2
comammox *amoA*	comaA-244f a-f comaA-659r a-f comaB-244f a-f comaB-659r a-f	See Pjevac et al., 2017	95°C for 30 s, 48 or 52°C for 45s 1 min at 72°C (45 cycles)	3
NOB-*Nitrospira* *nxrB*	nxrB169f nxrB638r	5′- TACATGTGGTGGAACA -3′ 5′- CGGTTCTGGTCRATCA -3’	95ºC for 5s, 56.2ºC for 30s, 72ºC for 30s (40cycles)	4
NOB-*Nitrobacter* *nxrB*	nxrB-1F nxrB-1R	5’-ACGTGGAGACCAAGCCGGG-3’ 5’-CCGTGCTGTTGAYCTCGTTGA-3’	95ºC for 5s, 57ºC for 20s, 72ºC for 30s (40cycles)	5
Bacteria 16S rRNA	Eub 338 Eub 518	5′- ACTCCTACGGGAGGCAGCAG -3′ 5′- ATTACCGCGGCTGCTGG -3’	95ºC for 15s, 60ºC for 20s, 72ºC for 10s (35 cycles)	6
Thaumarchaea 16S rRNA	771F 957R	5′- ACG GTG AGG GAT GAA AGC T -3′ 5′- ACG GTG AGG GAT GAA AGC T -3′	95ºC for 3s, 55ºC for 30s, 72ºC for 55s (35 cycles)	7
Fungi 18S rRNA	FR1 FF 390	5′- AICCATTCAATCGGTAIT -3′ 5′- CGATAACGAACGAGACCT -3’	95ºC for 15s, 50ºC for 35s, 72ºC for 10s (40 cycles)	8
**Amplicon sequencing analyses**				
Bacteria 16S rRNA	515f 806r	NNNNNNNNN**GT**GTGYCAGCMGCCGCGGTAA[a] GGACTACNVGGGTWTCTAAT	98°C for 10 s, 50°C for 30 s, 72°C for 30 s (25 + 7 cycles) [b]	9
Fungi ITS	ITS-7 ITS-4	GTGARTCATCGAATCTTTG NNNNNNNNNG**A**TCCTCCGCTTATTGATATGC[a]	98°C for 10 s, 55°C for 30 s, 72°C for 30 s (28 +7 cycles) [b]	10, 11
AOB	amoA-1f	NNNNNNN**AA**GGGGTTTCTACTGGTGGT[a]	98°C for 10 s, 54°C for 30 s,	1

amoA	amoA-2r	CCCCTCKGSAAAGCCTTCTTC	72°C for 60 s (30 +7 cycles) [b]	
AOA<br>*amoA*	amoA-310f<br>amoA-529r	NNNNNNNNNN**GG**TGGATACCBTCWGCAATG[a]<br>GCAACMGGACTATTGTAGAA	98°C for 10 s, 54°C for 30 s,<br>72°C for 60 s (30+7 cycles) [b]	12

[a]The sample index (consecutive Ns) and linker (bold letters) prior to the extension bases in the forward or reverse primer are indicated.
[b]The first number in parentheses indicates the number of cycles performed in the first PCR where the unindexed primers were used, while the second number indicates the additional cycles performed in the sample indexing PCR.

TABLE S7 Sample associated primer index sequences (5′ - 3′) (represented by Ns in Table 3) associated with the primers (i) 515f for bacteria (ii) ITS-4r for fungi (iii) amoA- 1f for AOB; (iv) amoA-310f for AOA.

No. primer index	16S rRNA 515f	ITS-4r	AOB amoA-1f	AOA amoA-310f
1	TTCTTCTTC	TTATTACCG	TTATTACGC	TTATTACCG
2	TTCTCAATG	TTATTAGGC	TTATTCGTC	TTATTAGGC
3	TTCAGTTCA	TTATTCTCC	TTATATGGC	TTATACTCC
4	TTCGAATCA	TTATTCGTG	TTATCTCTC	TTATACAGG
5	TTGTCAGGT	TTATTGCGA	TTATCCTAC	TTATAGCTG
6	TTGAAGTTC	TTATACTGG	TTATCGAGC	TTATAGGAC
7	TTGCAACAA	TTATACCTC	TTATGGTTC	TTATGTTGG
8	TTGGACGAC	TTATACGCA	TTAATCTGC	TTATGTCTC
9	TTCTTCAAG	TTATAGACC	TTAATGCTC	TTATGAACC
10	TTCTCAGAA	TTATGTTCG	TTAAGCATC	TTATGACGT
11	TTCAGTAAG	TTATGTGAC	TTACTTGAC	TTATGAGAG
12	TTCGACAAT	TTATGAAGG	TTACAATGC	TTATGGTCA
13	TTGTCGATA	TTATGAGCT	TTACGTCGC	TTAATCTCG
14	TTGAAGGAA	TTATGCCAT	TTCTATCCT	TTAATCAGC
15	TTGCAGTAT	TTATGGTGT	TTCTACTTC	TTAATGGTG
16	TATATCAGG	TTAATTCGC	TTCTACGAT	TTAACTACC
17	TTCTTGTCA	TTAATCCAG	TTCTCTTGC	TTAACTCTG
18	TTCATATGG	TTAATCGGT	TTCTCTGTT	TTAACTGGA
19	TTCAGACTT	TTAATGTGG	TTCTCATCT	TTAACAAGG
20	TTCGAGCAC	TTAATGCCT	TTCTCGCAT	TTAACACAC
21	TTGTGTATC	TTAATGGAC	TTCTGTGAC	TTAACCGTT
22	TTGACTATG	TTAACTTCC	TTCTGACTC	TTAACGTAG
23	TTGCCTAGT	TTAACTAGG	TTCTGGATT	TTAACGCCT
24	TATATCGTC	TTAACAGTC	TTCATTCGC	TTAAGTCCA
25	TTCTTGAGT	TTAACCTTG	TTCATCGTT	TTAAGTGAC
26	TTCATAGTC	TTAACCGAA	TTCATGTCT	TTAAGAGCT
27	TTCAGAGGA	TTAACGACA	TTCATGGAC	TTAAGCATG
28	TTGTTCAGA	TTACTTACG	TTCAAGCTT	TTAAGGAGT
29	TTGTGTGAA	TTACTTGTC	TTCCGTTAT	TTACTTACG
30	TTGACGTGA	TTACTAGAG	TTCCGAGCT	TTACTTGTC
31	TTGCCTCAC	TTACTCTGA	TTCCGCTGC	TTACTATGG
32	TATATGCAC	TTACTCCTT	TTCCGCCTT	TTACTAGCA
33	TTCTTGGAC	TTACTGGCA	TTCGTTACT	TTACTCCTT
34	TTCATCACA	TTACATTGC	TTCGTTGTC	TTACTCGAG
35	TTCAGCAGT	TTACAGTAG	TTCGTCTAT	TTACATCAC
36	TTGTTCGTT	TTACAGGTT	TTCGTGTGC	TTACAACCT
37	TTGTGACTA	TTACCTAAC	TTCGACATT	TTACAAGTG
38	TTGACGAAT	TTACCTCTA	TTCGACGGC	TTACACTGT
39	TTGCCAATC	TTACCTGGT	TTCGCATAC	TTACACATC
40	TATAACGAG	TTACCATCG	TTCGCCGCT	TTACAGTCG
41	TTCTATAGG	TTACCGTTC	TTCGCGATC	TTACAGAGA

42	TTCATCGAT	TTACGTCAG	TTGTTCTCT	TTACCTAGT
43	TTCAGCCAA	TTACGATAC	TTGTTGCTT	TTACCACTA
44	TTGTTGTAG	TTACGACCA	TTGTCTACT	TTACCAGAT
45	TTGTGCAAT	TTACGCCGC	TTGTGTCAT	TTACCGAAC
46	TTGAGTTGG	TTACGCGTA	TTGTGCGTT	TTACCGCGG
47	TTGCCAGAG	TTAGTTCTG	TTGATTCCT	TTACGATAC
48	TATAAGTGG	TTAGTTGGA	TTGACTGAT	TTACGCTTA
49	TTCTATCTC	TTAGTAACC	TTGAGCTAT	TTACGCAAT
50	TTCAATCGT	TTAGTACGT	TTGAGGACT	TTACGCCGC
51	TTCAGGTAT	TTAGATCCT	TTGCTTGTT	TTAGTACGA
52	TTGTATCGA	TTAGATGAG	TTGCTACAT	TTAGTCTAC
53	TTGTGGTGT	TTAGACTAC	TTGCATTCT	TTAGTCGCT
54	TTGAGTCAT	TTAGACATG	TTGCCGATT	TTAGTGTGT
55	TTGGTTGTC	TTAGAGTCA	TTGGTGGAT	TTAGTGATC
56	TATAAGCCA	TTAGCAGAT	TTGGATCTT	TTAGTGGAA
57	TTCTATGCA	TTAGCCTGT	TTGGCGTCT	TTAGATAGC
58	TTCAATGAC	TTAGGTACA	TATTAAGGC	TTAGAACTC
59	TTCGTTCTA	TTAGGCGCC	TATTCTCCT	TTAGAAGGT
60	TTGTAATGG	TTCTTATGG	TATTCTGAC	TTAGACTTG
61	TTGATAGCA	TTCTTACTC	TATTCCTTC	TTAGACCAT
62	TTGAGAGTG	TTCTTAGCA	TATTCGCGC	TTAGAGAAG
63	TTGGTATGA	TTCTTCAGT	TATTCGGTT	TTAGCTCAA
64,	TATAGTCTC	TTCTTCGAC	TATTGTTGC	TTAGCATCT
65	TTCTAACAG	TTCTTGAAG	TATTGAGCT	TTAGCCTGA
66	TTCAACTAG	TTCTTGGTT	TATTGGATC	TTAGCCACG
67	TTCGTTGGT	TTCTATTCC	TATATCCTC	TTAGCGGCC
68	TTGTAAGTC	TTCTATAGG	TATAATCGC	TTAGGCGTC
69	TTGATCTTG	TTCTAACAG	TATAAGCCT	TTGTTAAGG
70	TTGAGCCTC	TTCTACCGA	TATAAGGTC	TTGTTACTC
71	TTGGTCTAT	TTCTAGTTG	TATACATGC	TTGTTGTTG
72	TATAGACAG	TTCTAGCCT	TATCTTGTC	TTGTTGAAC
73	TTCTAGTTG	TTCTAGGAA	TATCTAAGC	TTGTTGGCT
74	TTCAACATC	TTCTCTTAG	TATCTCGCT	TTGTAATCG
75	TTCGTAGAG	TTCTCTACA	TATCCGTCT	TTGTACTGA
76	TTGTAGAAC	TTCTCTCTT	TATCGATTC	TTGTACACT
77	TTGATCAAC	TTCTCTGGC	TATCGCGAC	TTGTACCAG
78	TTGAGGACA	TTCTCCATC	TATCGGCTT	TTGTACGTC
79	TTGGTGCCA	TTCTCCGCT	TATGTCTGC	TTGTAGCGC
80	TATAGAGGT	TTCTCGTGA	TATGTGACT	TTGTCTTAC
81	TTCTCTTGT	TTCTGTGTA	TATGATGCT	TTGTCTATG
82	TTCAAGAGA	TTCTGAACC	TATGACATC	TTGTCTCCT
83	TTCGTGATC	TTCTGACGT	TATGGACGC	TTGTCAACA
84	TTGTCTTCA	TTCTGCTCA	TATGGCTCT	TTGTCAGTT
85	TTGATGAGG	TTCATTGTG	TAATTGTGC	TTGTCCGAA
86	TTGCATAAG	TTCATCTTC	TAATTGCCT	TTGTCGTGT
87	TTGGAACTT	TTCATGTCA	TAATACGAC	TTGTGTAGT
88	TATAGCAAC	TTCAGTTAC	TAATCCGCT	TTGTGTCAA
89	TTCTCTAAC	TTCAGTCCT	TAATGTGTC	TTGTGAGGA

90	TTCAAGGTT	TTCAGATTG	TAAGCTTAC	TTGTGGATA
91	TTCGATGTG	TTCAGAAGA	TAAGCAAGC	TTGTGGCCG
92	TTGTCTCTT	TTCAGCTGT	TAAGCCGTC	TTGATAACC
93	TTGAACTCA	TTCAGGCTA	TAAGCGCAT	TTGATAGAG
94	TTGCATGTT	TTCCTTCAT	TAAGGTCCT	TTGATCTGT
95	TTGGACATA	TTCCTAATG	TAAGGCAAT	TTGATCGCA
96	TATAGGATG	TTCCTACGA	TACTATCTC	TTGAATACG

Supplemental figures

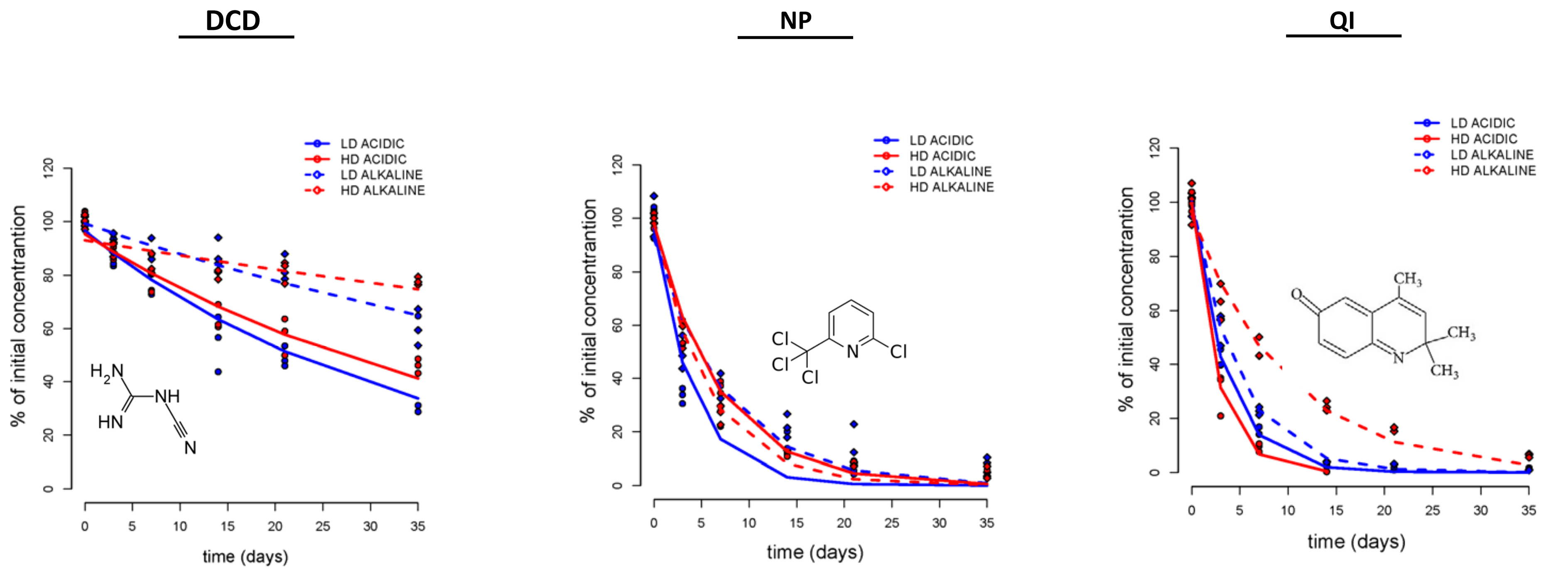

FIG S1 The dissipation pattern of the low (blue line) and the high (red line) dose rate of DCD, NP, and QI in the acidic (solid line) and the alkaline soil (dash line), fitted to the single first-order (SFO) kinetics model.

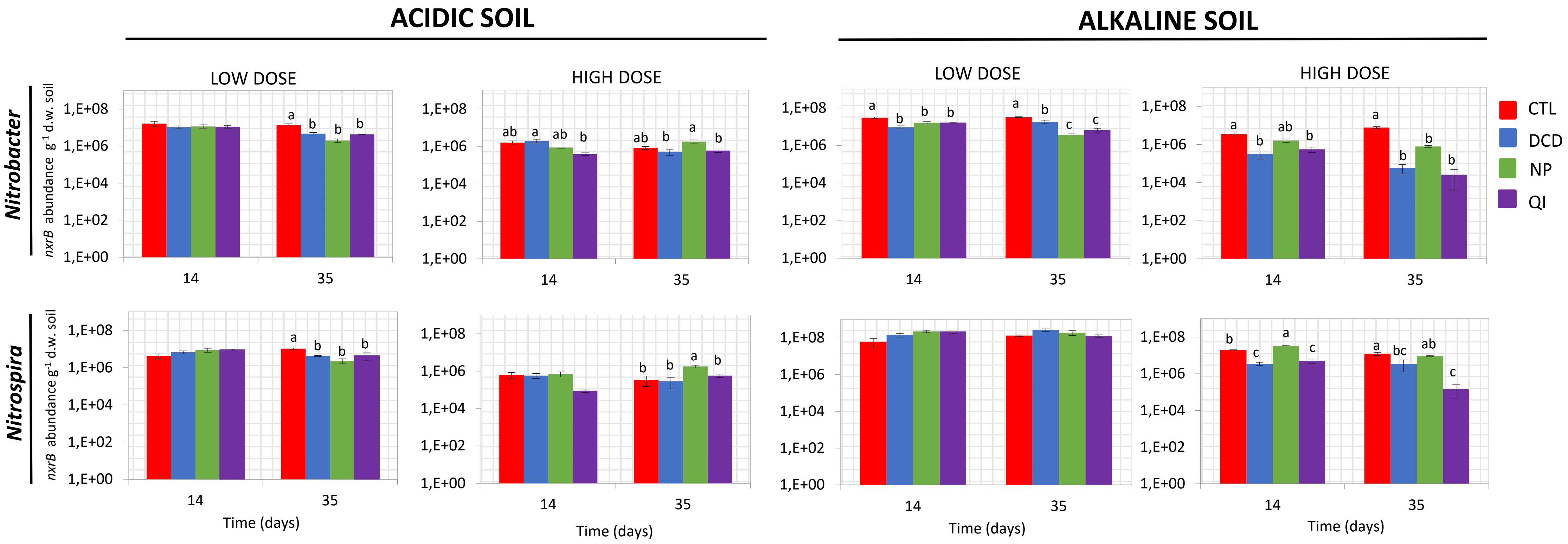

FIG S2 The effect of DCD, NP, and QI applied at low and high dose rates on *nxrB* gene abundance of *Nitrobacter* and *Nitrospira* in the two studied soils (acidic and alkaline). Each value is mean of three replicates ± standard error. Within each time point, groups designated by the same letter are not significantly different at the selected p -value levels.

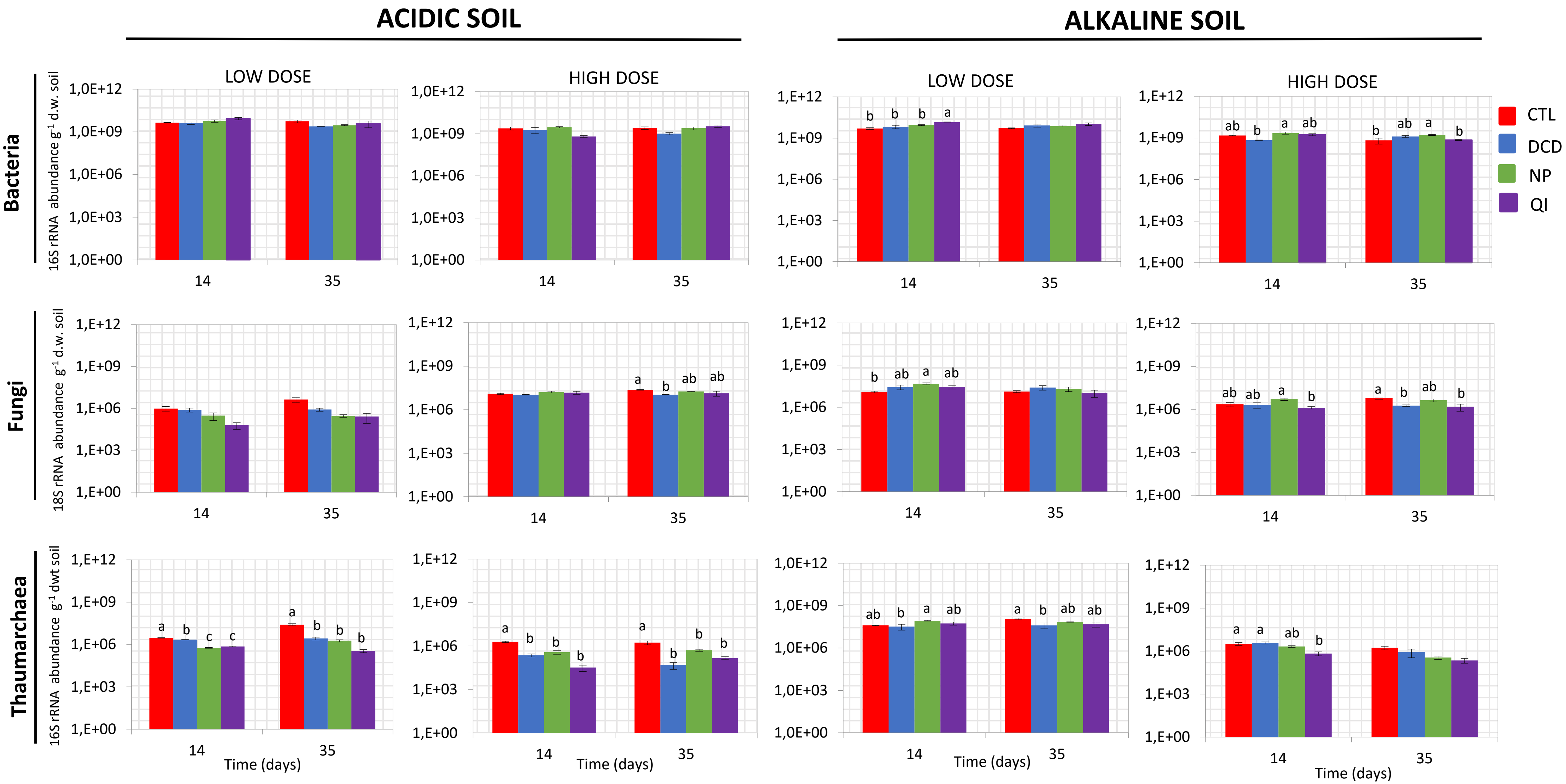

FIG S3 The effect of DCD, NP, and QI applied at low and high dose rates on the abundance of bacteria, fungi and Thaumarchaeota in the acidic soil and the alkaline soil. Each value is mean of three replicates ± standard error.Within each time point, groups designated by the same letter are not significantly different at the selected p-value levels.

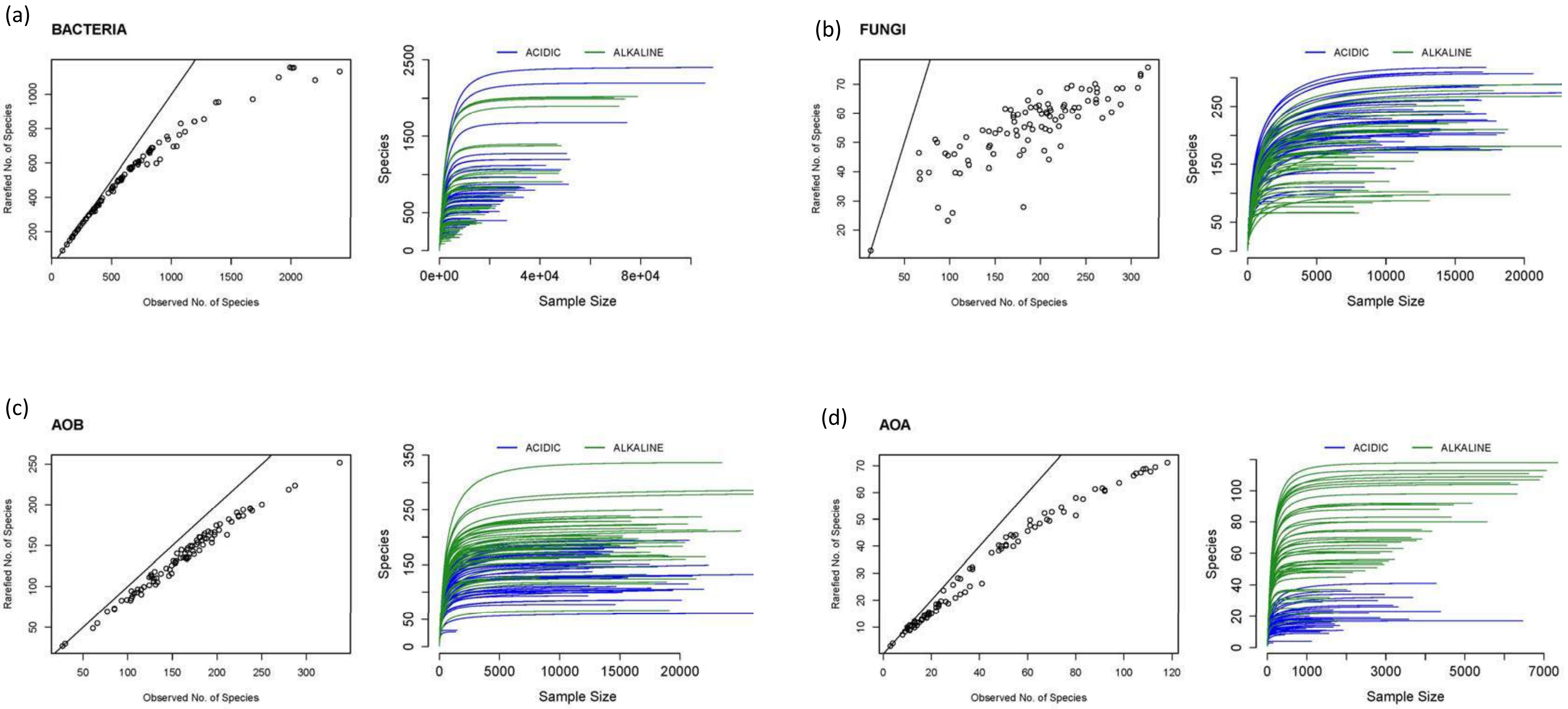

FIG S4 Scatterplots showing the observed and rarefied number of ASVs for each sample (left panel) and rarefaction curves denoting the diversity coverage obtained by our sequencing effort (right panel) for the bacterial (a), fungal (b), AOB (c) and AOA (d) communities in the acidic (blue lines) and the alkaline (green lines) soil.

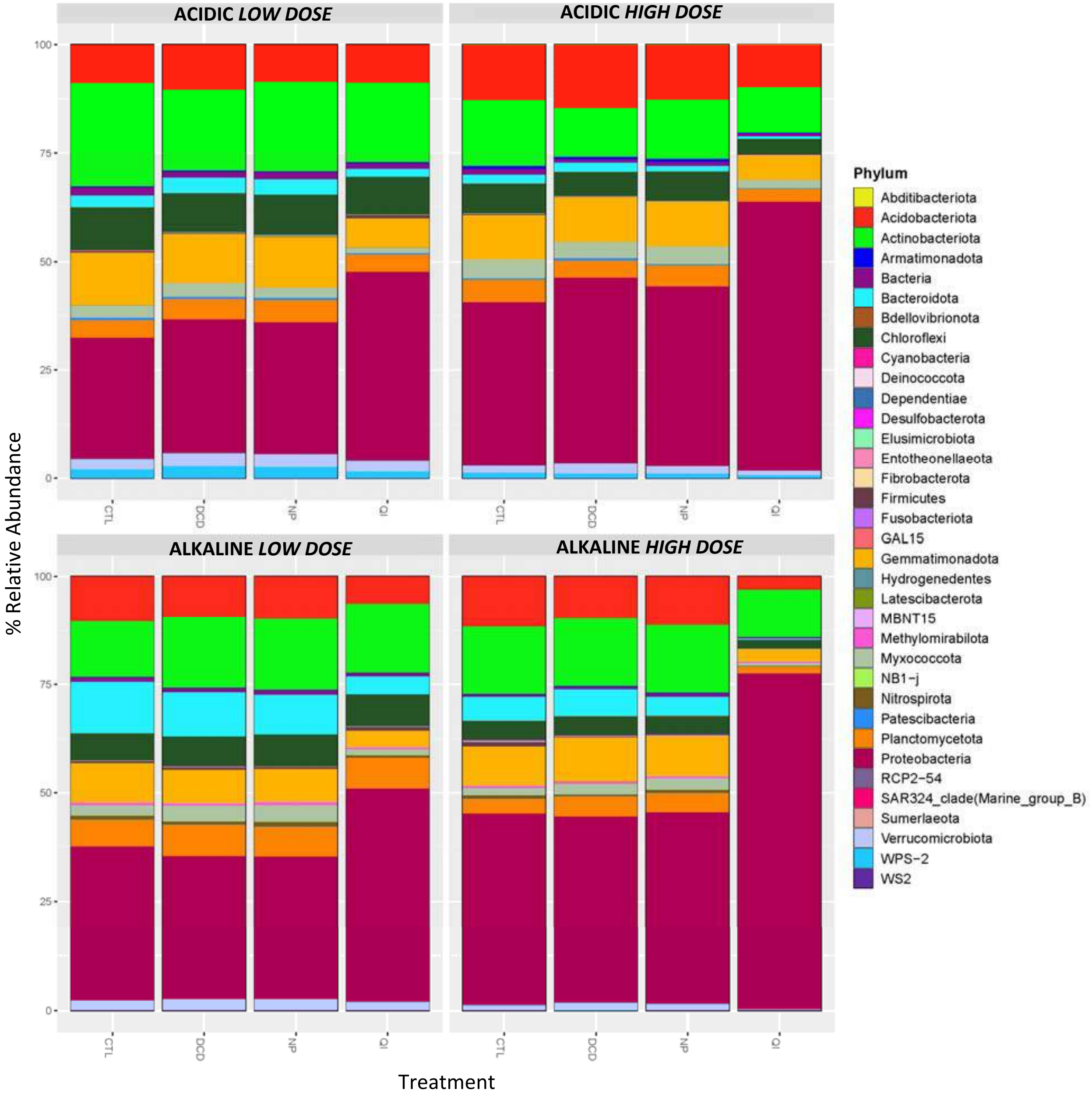

FIG S5 Stacked bar plots presenting soil bacterial taxonomic distribution at the phylum level in samples of the acidic and the alkaline soil treated (DCD, NP, QI low and high dose rates) or not treated with the NIs (CTL). The values presented for each treatment are the average of six biological replicates.

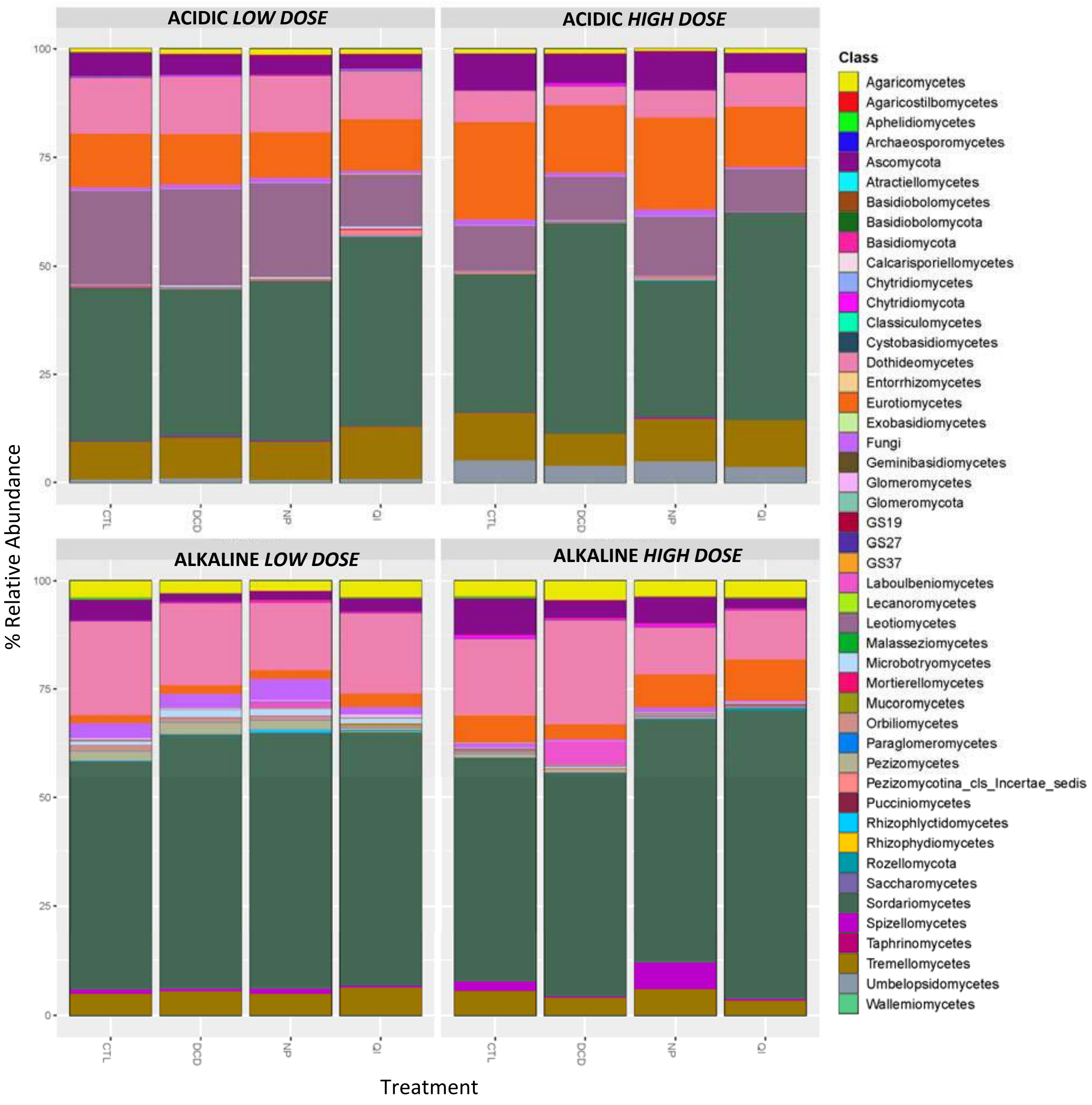

FIG S6 Stacked bar plots presenting soil fungal taxonomic distribution at the class level in samples of the acidic and the alkaline soil treated (DCD, NP, QI low and high dose rates) or not treated with the NIs (CTL). The values presented for each treatment are the average of six biological replicates.

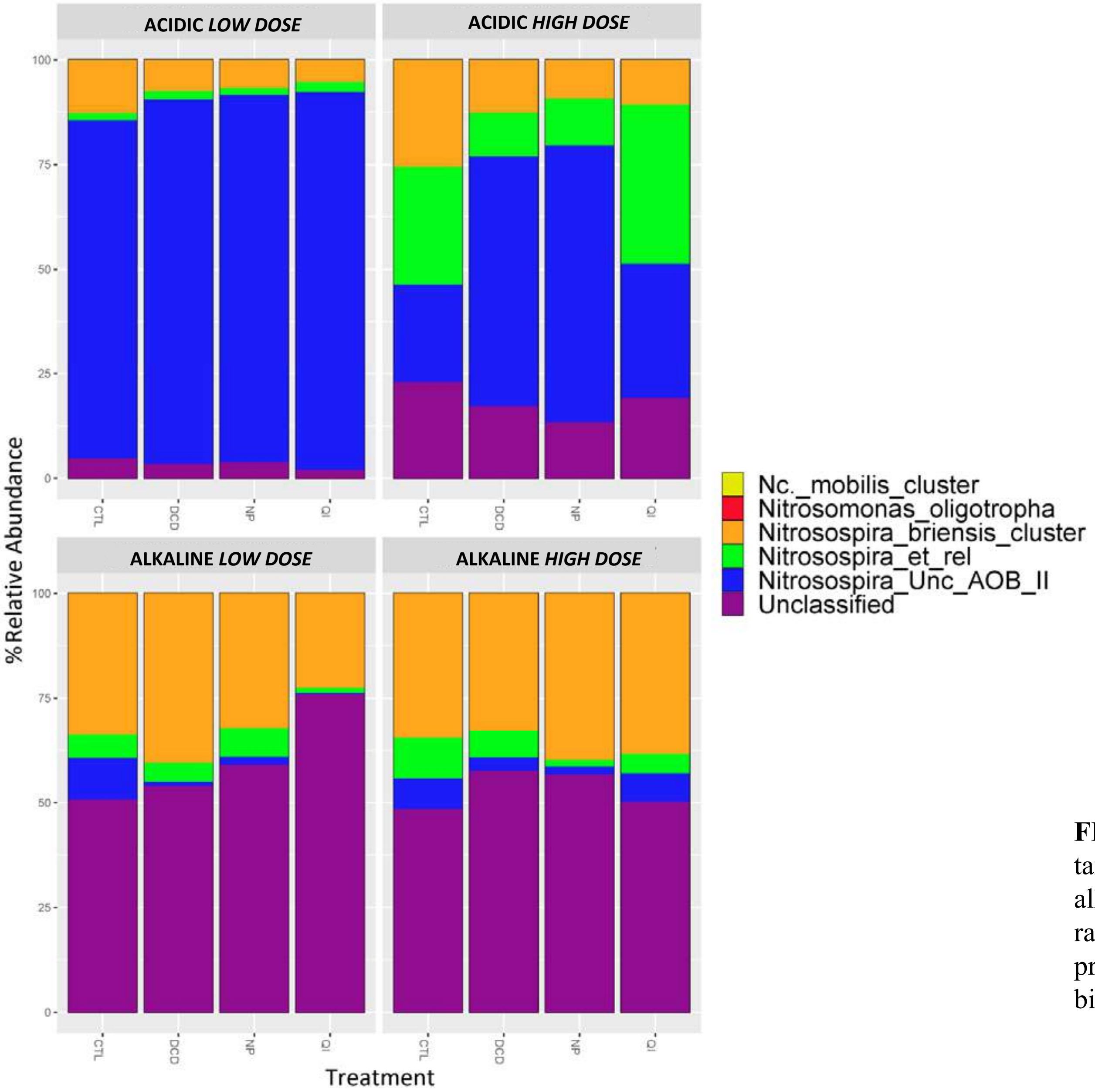

FIG S7 Stacked bar plots presenting soil AOB taxonomic distribution in samples of the acidic and the alkaline soil treated (DCD, NP, QI low and high dose rates) or not treated with the NIs (CTL). The values presented for each treatment are the average of six biological replicates.

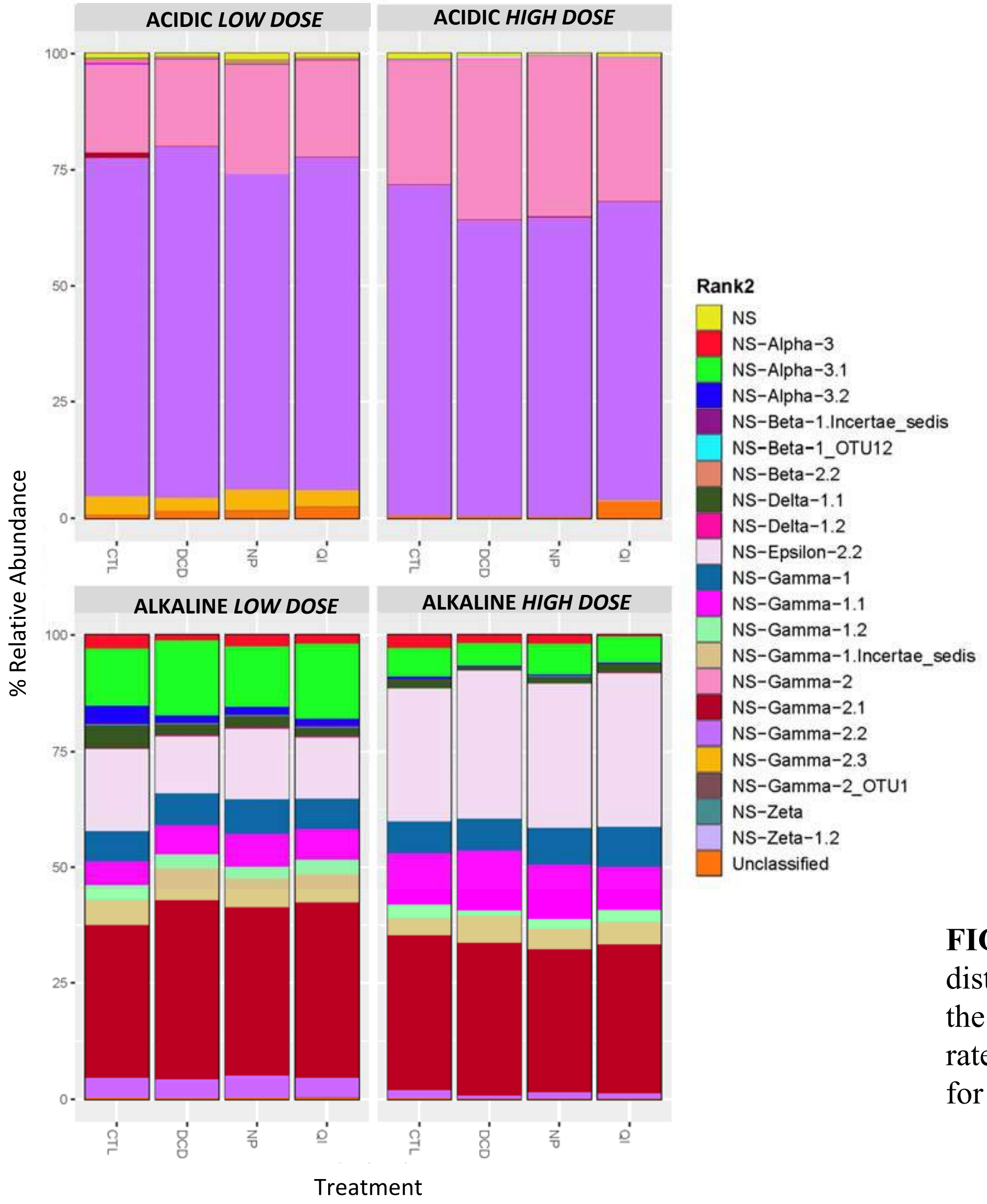

FIG S8 Stacked bar plots presenting soil AOA taxonomic distribution at the Rank 2 level in samples of the acidic and the alkaline soil treated (DCD, NP, QI low and high dose rates) or not treated with the NIs (CTL). The values presented for each treatment are the average of six biological replicates.

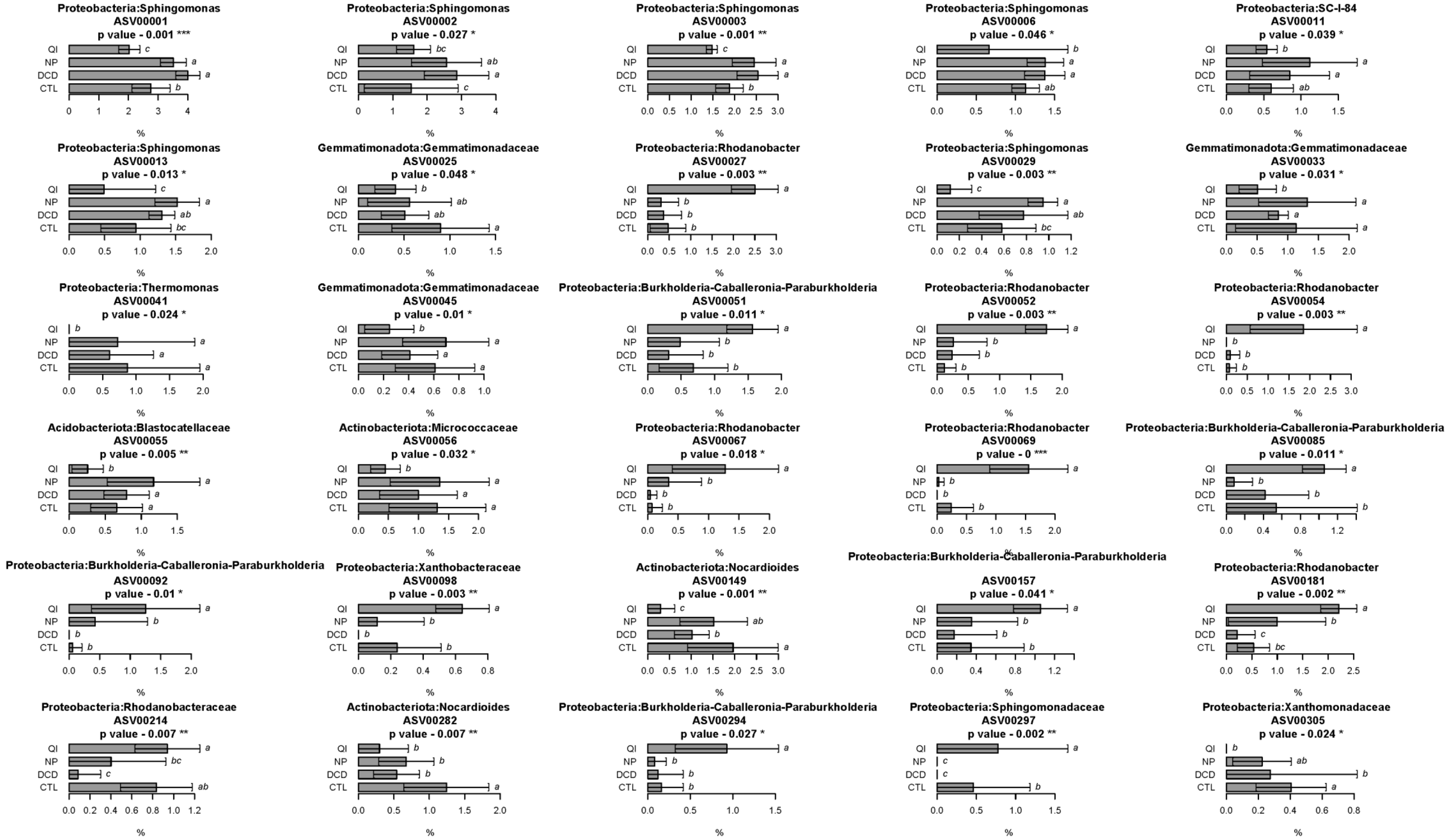

FIG S9a Barplots of the statistically significant differentially abundant bacterial ASVs in samples of the acidic soil treated (DCD, NP, QI low dose) or not treated with the NIs (CTL). The corresponding p-values, ÀSV taxonomies, and treatment relative abundances are provided. Each value is mean of six replicates ± standard error. Per ASV treatment groups designated by the same letter are not significantly different at the selected p -value levels.

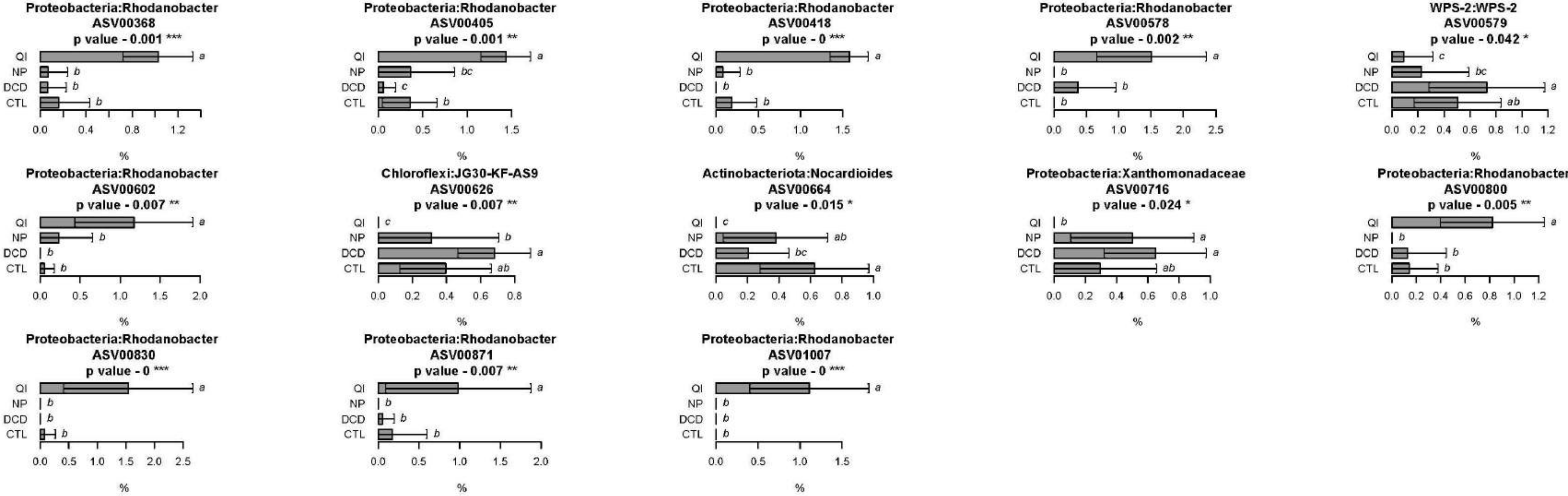

FIG S9b Barplots of the statistically significant differentially abundant bacterial ASVs in samples of the acidic soil treated (DCD, NP, QI low dose) or not treated with the NIs (CTL). The corresponding p-values, ÀSV taxonomies, and treatment relative abundances are provided. Each value is mean of six replicates ± standard error. Per ASV treatment groups designated by the same letter are not significantly different at the selected p -value levels.

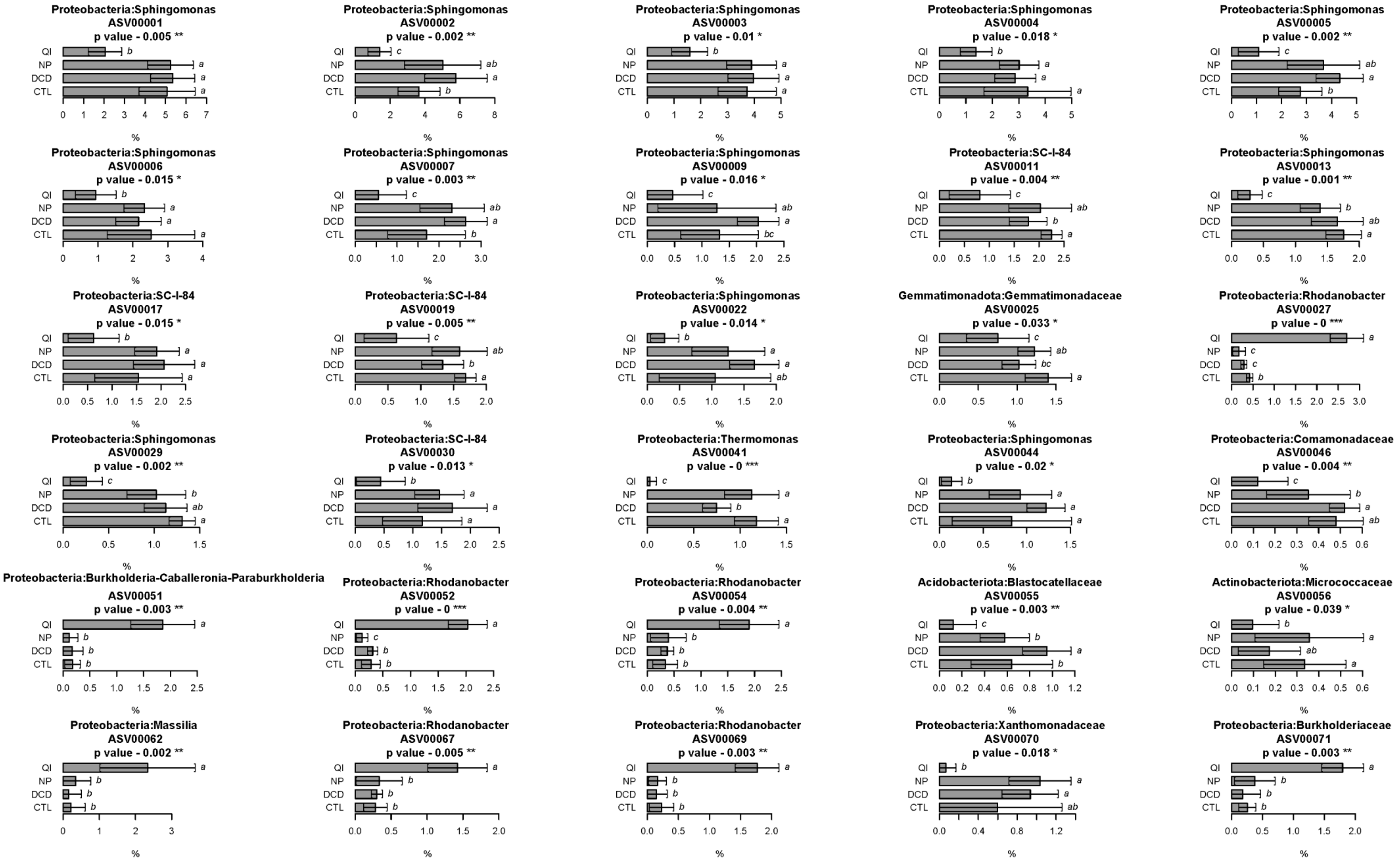

FIG S10a Barplots of the statistically significant differentially abundant bacterial ASVs in samples of the acidic soil treated (DCD, NP, QI high dose) or not treated with the NIs (CTL). The corresponding p-values, ÀSV taxonomies, and treatment relative abundances are provided. Each value is mean of six replicates ± standard error. Per ASV treatment groups designated by the same letter are not significantly different at the selected p -value levels.

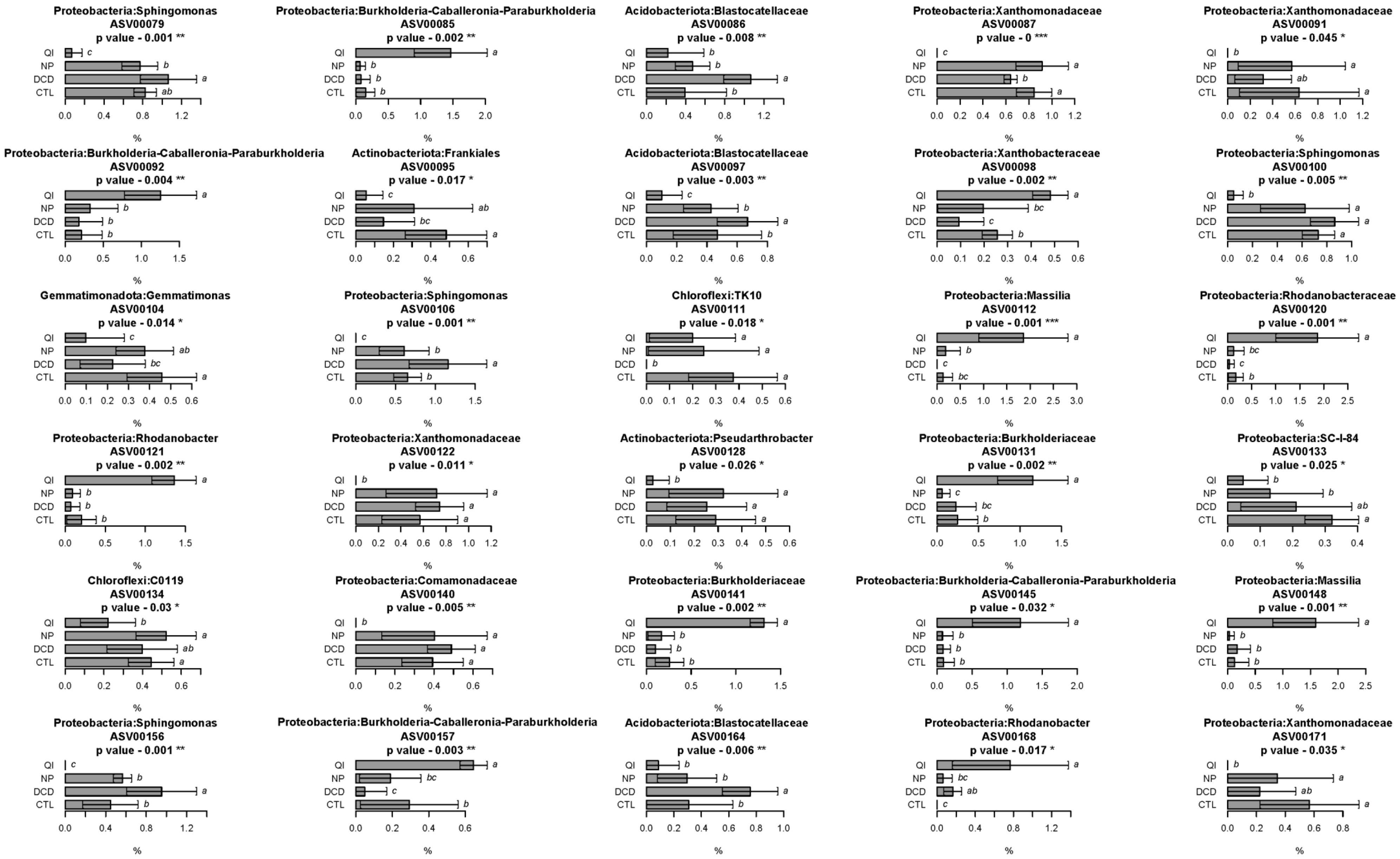

FIG S10b Barplots of the statistically significant differentially abundant bacterial ASVs in samples of the acidic soil treated (DCD, NP, QI high dose) or not treated with the NIs (CTL). The corresponding p-values, ÀSV taxonomies, and treatment relative abundances are provided. Each value is mean of six replicates ± standard error. Per ASV treatment groups designated by the same letter are not significantly different at the selected p -value levels.

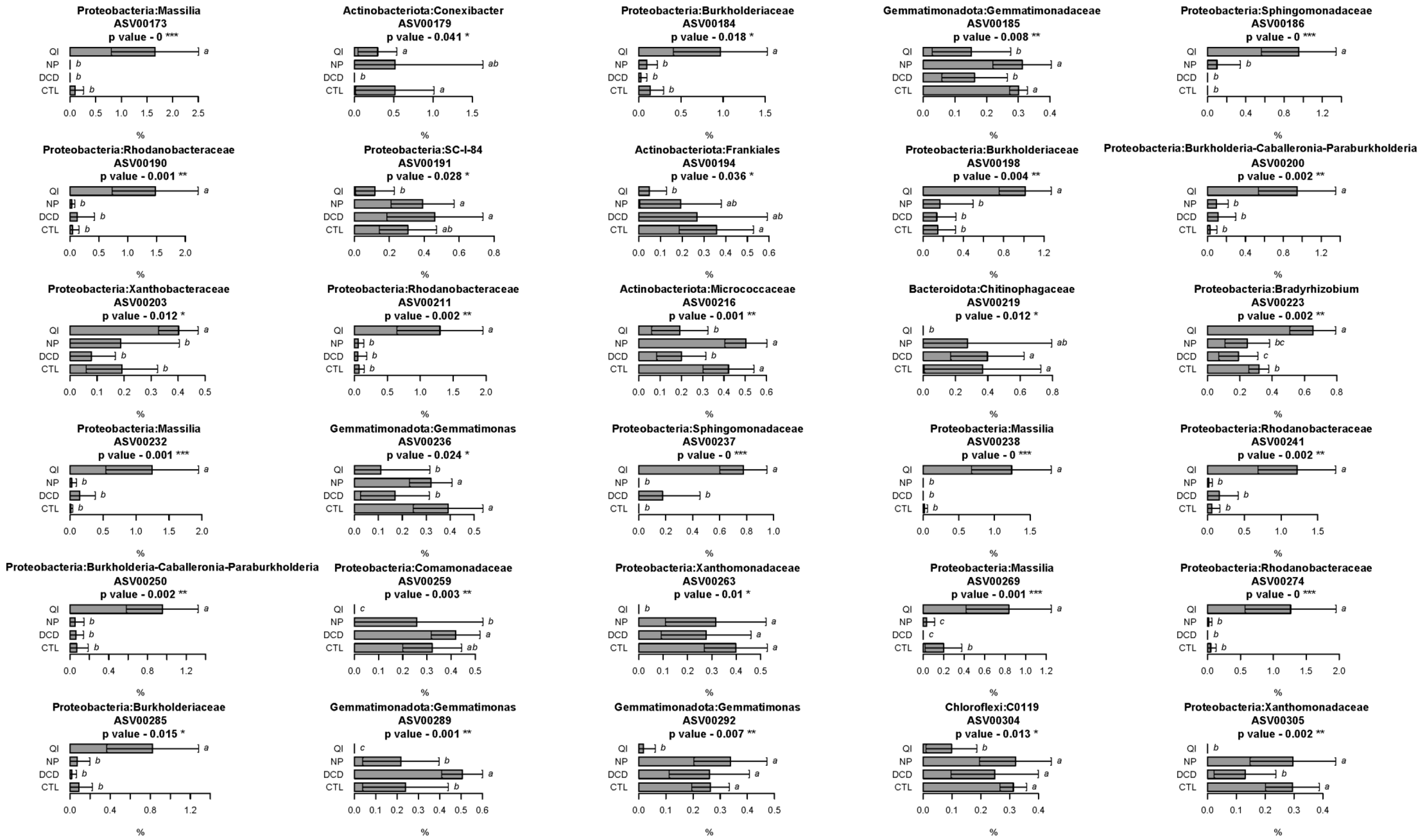

FIG S10c Barplots of the statistically significant differentially abundant bacterial ASVs in samples of the acidic soil treated (DCD, NP, QI high dose) or not treated with the NIs (CTL). The corresponding p-values, ÀSV taxonomies, and treatment relative abundances are provided. Each value is mean of six replicates ± standard error. Per ASV treatment groups designated by the same letter are not significantly different at the selected p -value levels.

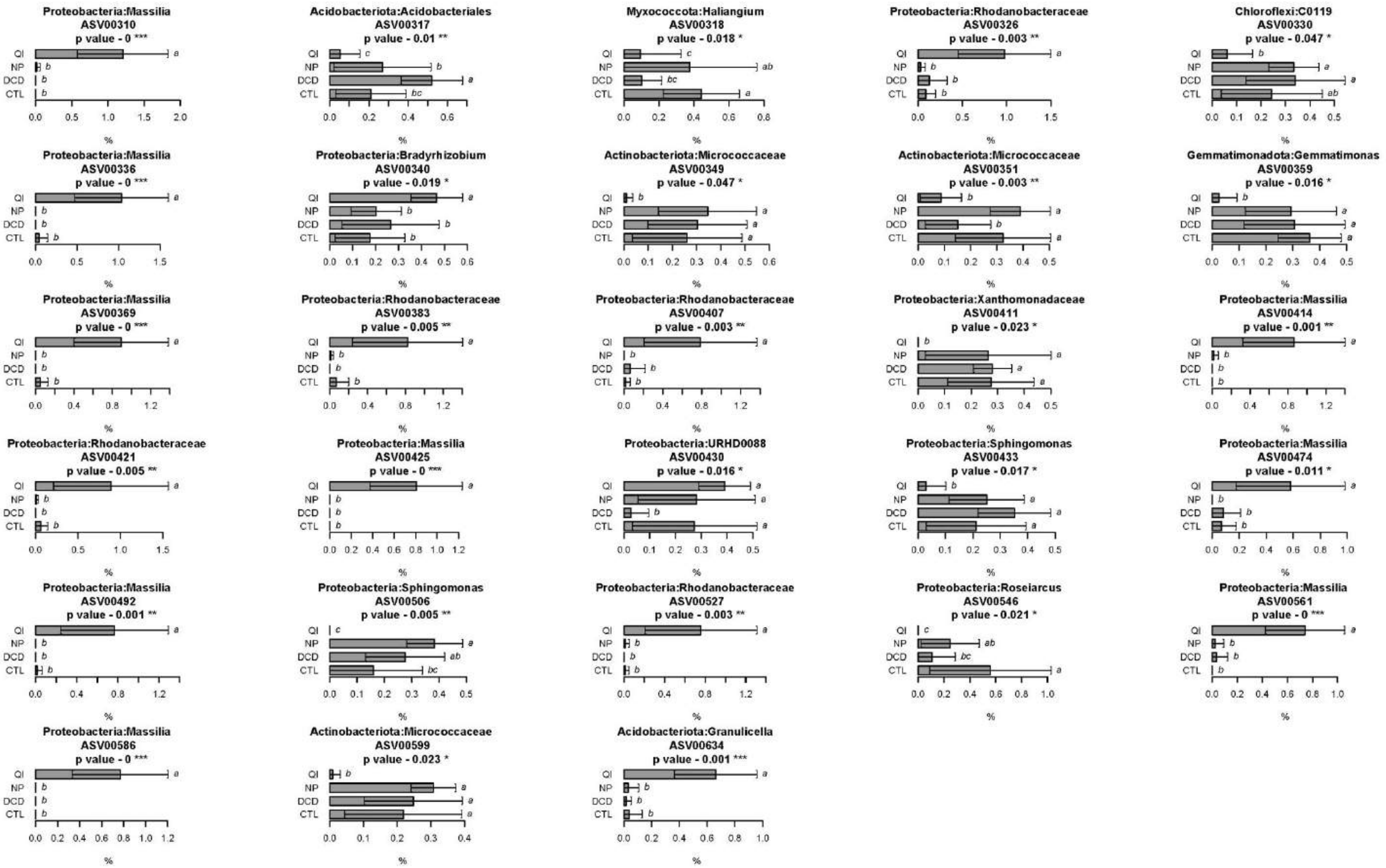

FIG S10d Barplots of the statistically significant differentially abundant bacterial ASVs in samples of the acidic soil treated (DCD, NP, QI high dose) or not treated with the NIs (CTL). The corresponding p-values, ÀSV taxonomies, and treatment relative abundances are provided. Each value is mean of six replicates ± standard error. Per ASV treatment groups designated by the same letter are not significantly different at the selected p -value levels.

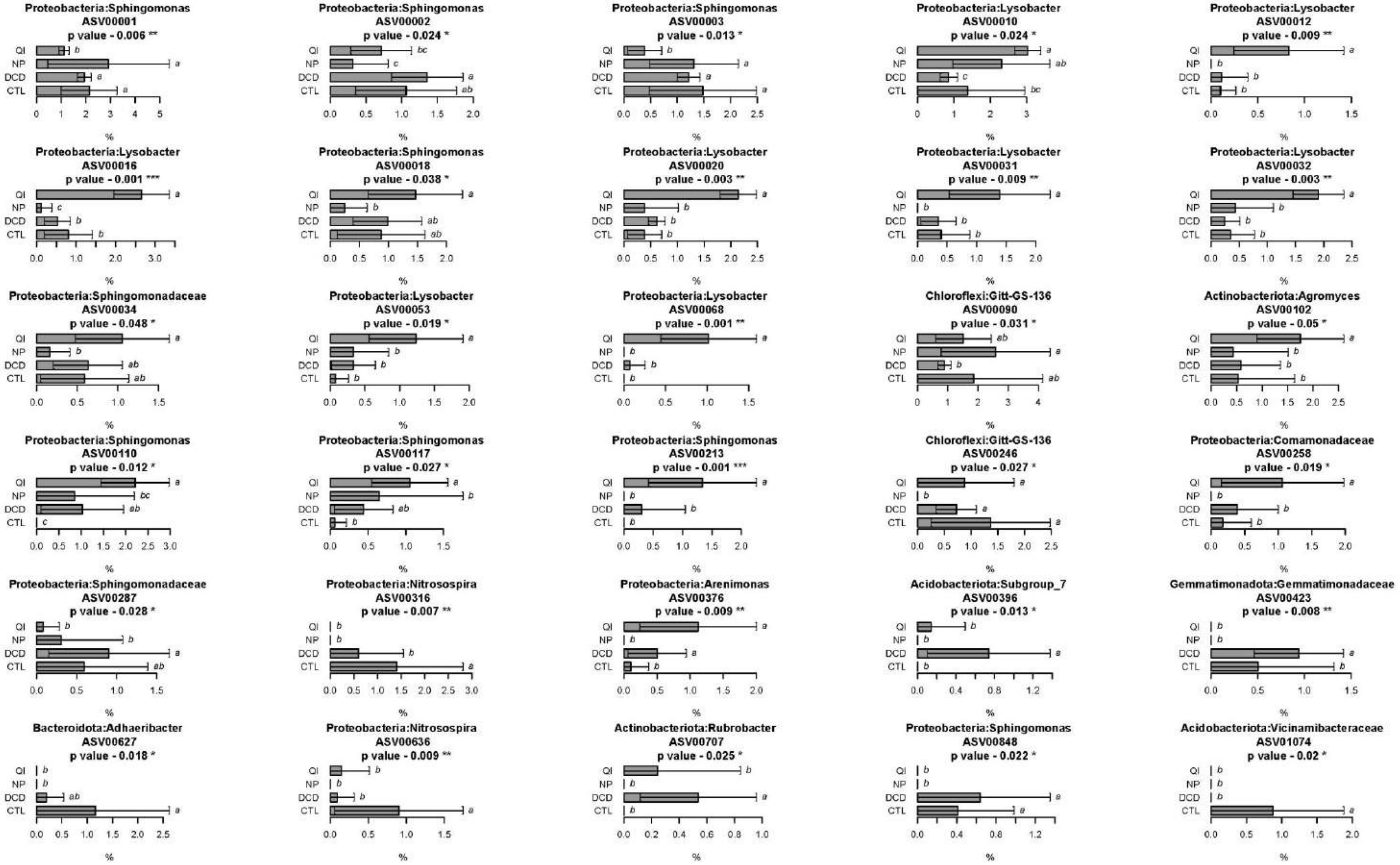

FIG S11a Barplots of the statistically significant differentially abundant bacterial ASVs in samples of the alkaline soil treated (DCD, NP, QI low dose) or not treated with the NIs (CTL). The corresponding p-values, ÀSV taxonomies, and treatment relative abundances are provided. Each value is mean of six replicates ± standard error. Per ASV treatment groups designated by the same letter are not significantly different at the selected p -value levels.

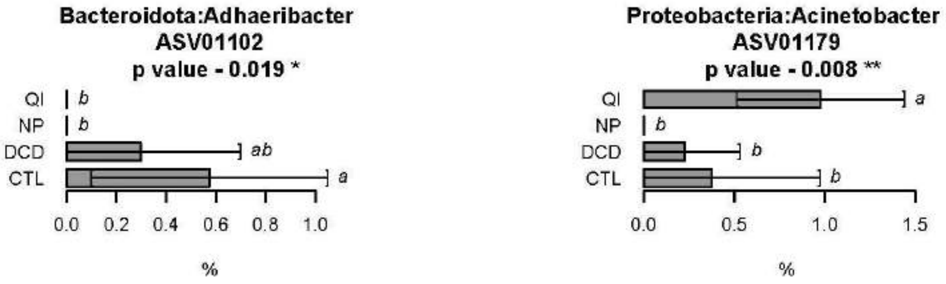

FIG S11b Barplots of the statistically significant differentially abundant bacterial ASVs in samples of the alkaline soil treated (DCD, NP, QI low dose) or not treated with the NIs (CTL). The corresponding p-values, ÀSV taxonomies, and treatment relative abundances are provided. Each value is mean of six replicates ± standard error. Per ASV treatment groups designated by the same letter are not significantly different at the selected p -value levels.

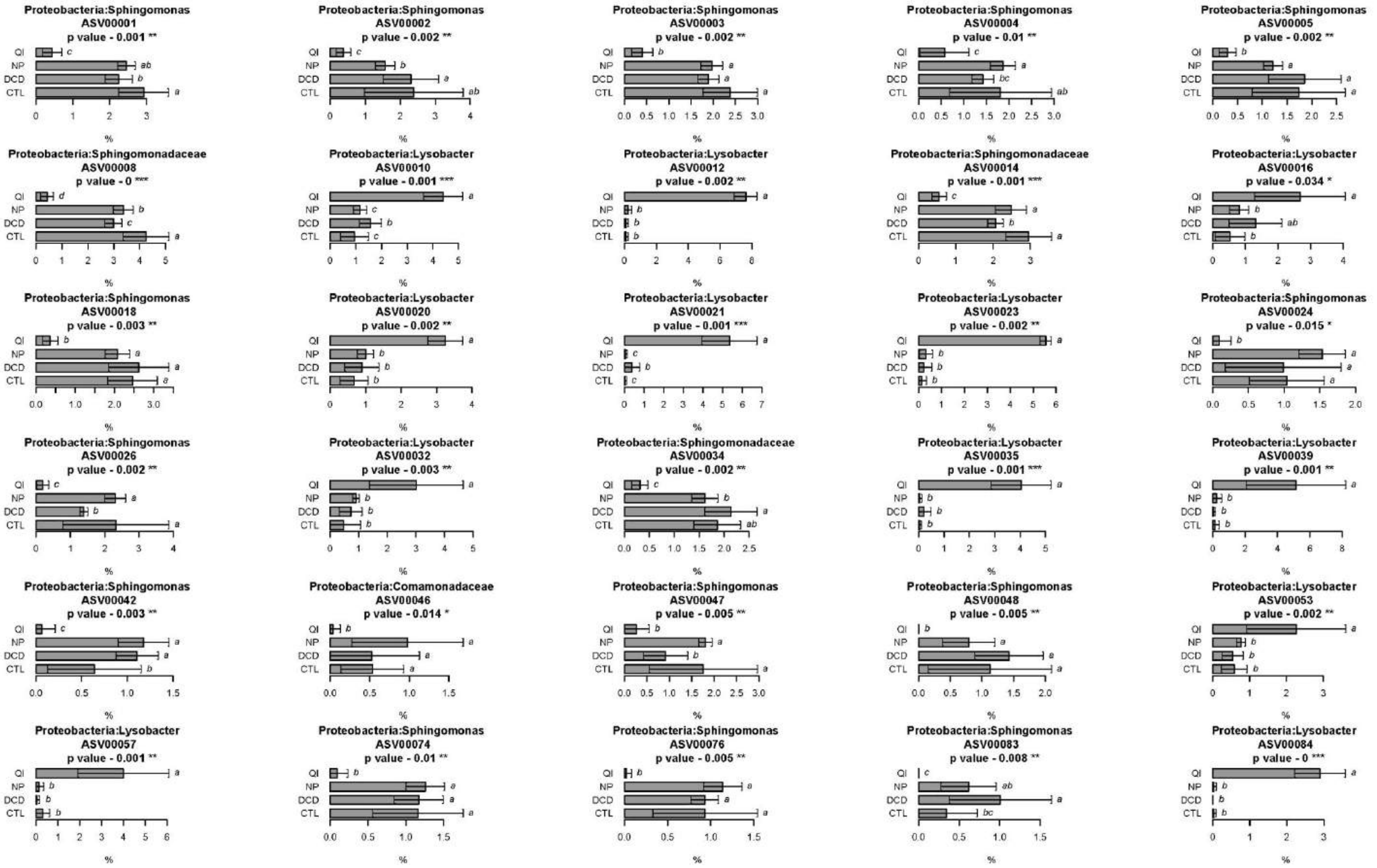

FIG S12a Barplots of the statistically significant differentially abundant bacterial ASVs in samples of the alkaline soil treated (DCD, NP, QI high dose) or not treated with the NIs (CTL). The corresponding p-values, ÀSV taxonomies, and treatment relative abundances are provided. Each value is mean of six replicates ± standard error. Per ASV treatment groups designated by the same letter are not significantly different at the selected p -value levels.

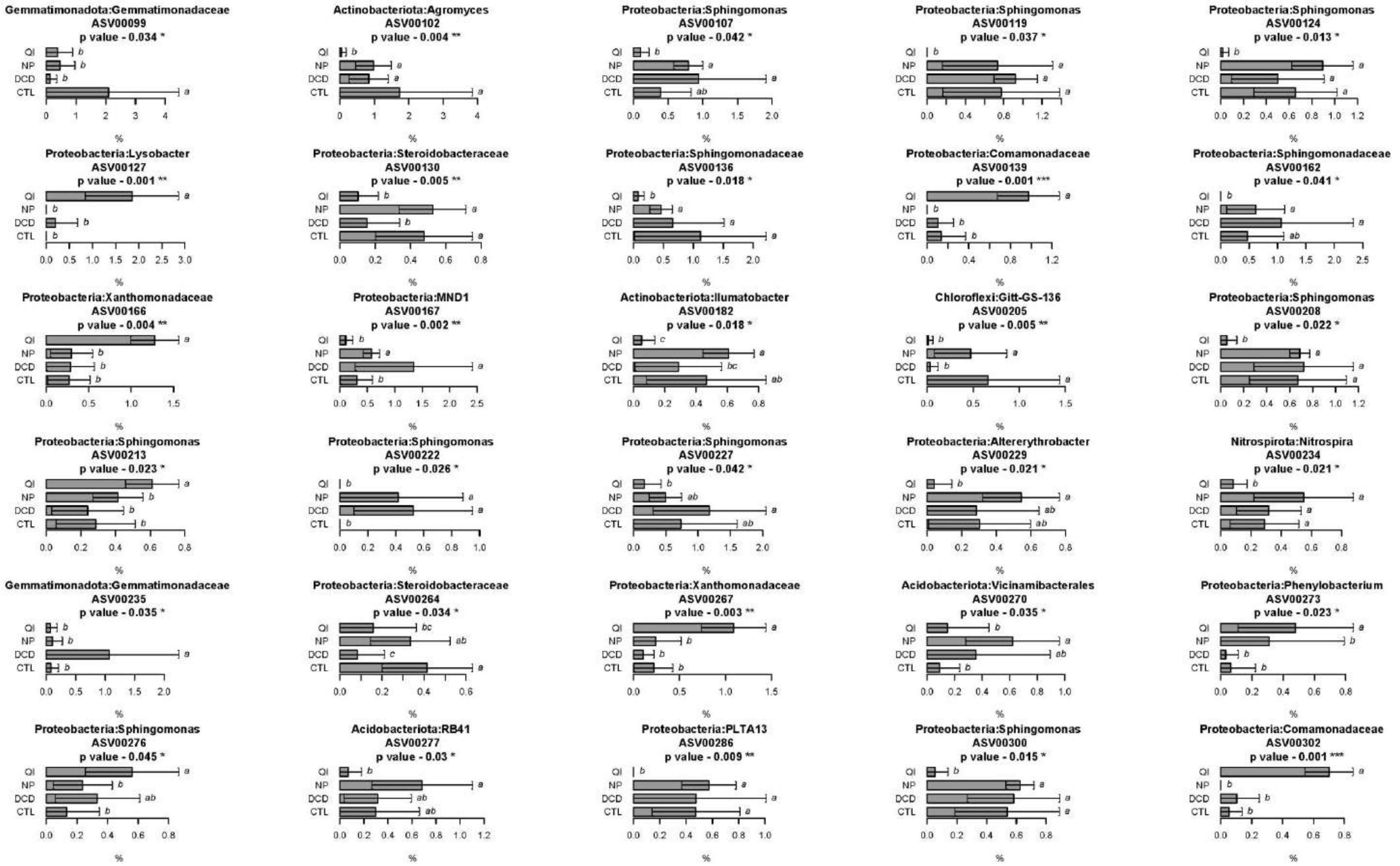

FIG S12b Barplots of the statistically significant differentially abundant bacterial ASVs in samples of the alkaline soil treated (DCD, NP, QI high dose) or not treated with the NIs (CTL). The corresponding p-values, ÀSV taxonomies, and treatment relative abundances are provided. Each value is mean of six replicates ± standard error. Per ASV treatment groups designated by the same letter are not significantly different at the selected p -value levels.

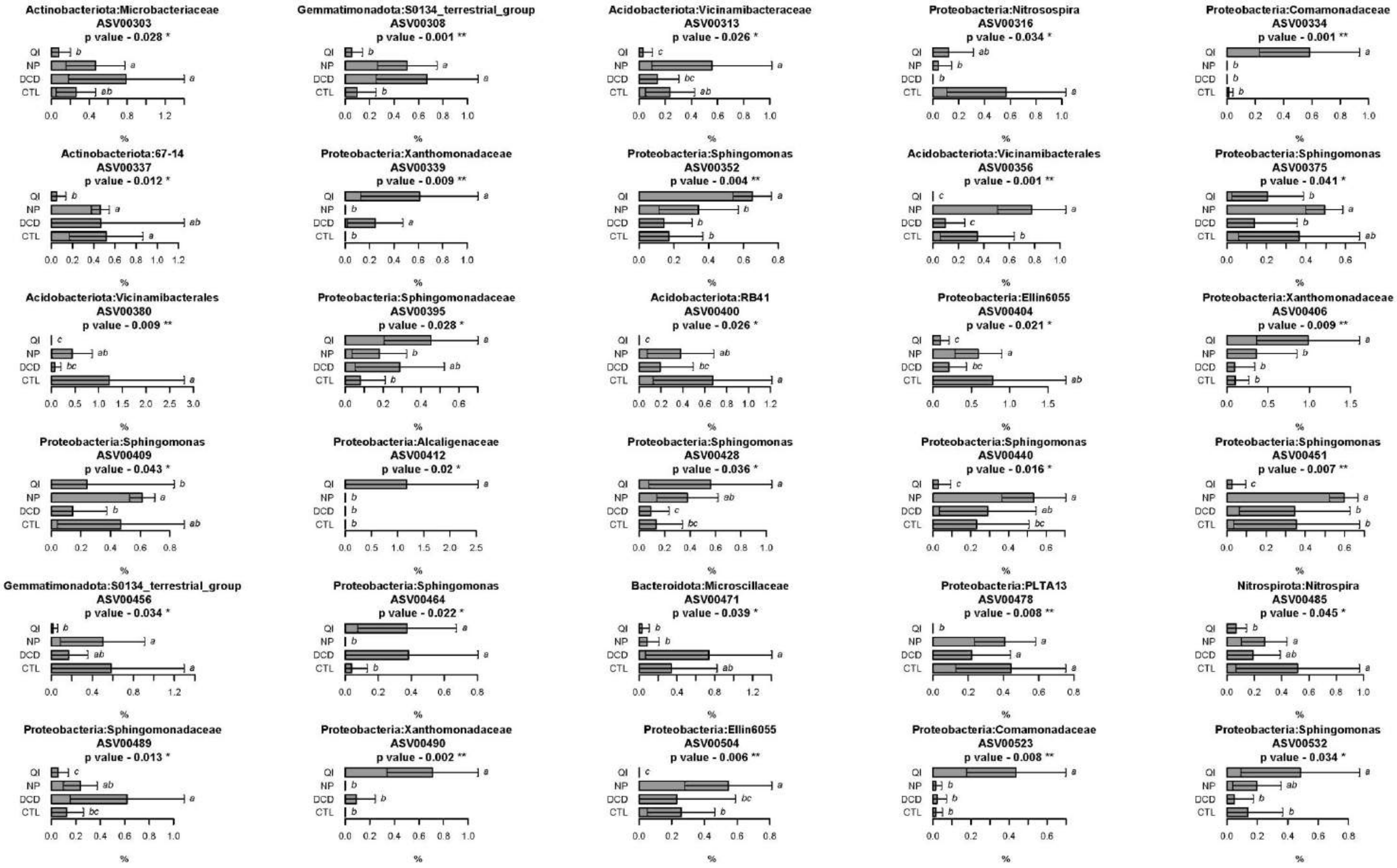

FIG S12c Barplots of the statistically significant differentially abundant bacterial ASVs in samples of the alkaline soil treated (DCD, NP, QI high dose) or not treated with the NIs (CTL). The corresponding p-values, ÀSV taxonomies, and treatment relative abundances are provided. Each value is mean of six replicates ± standard error. Per ASV treatment groups designated by the same letter are not significantly different at the selected p -value levels.

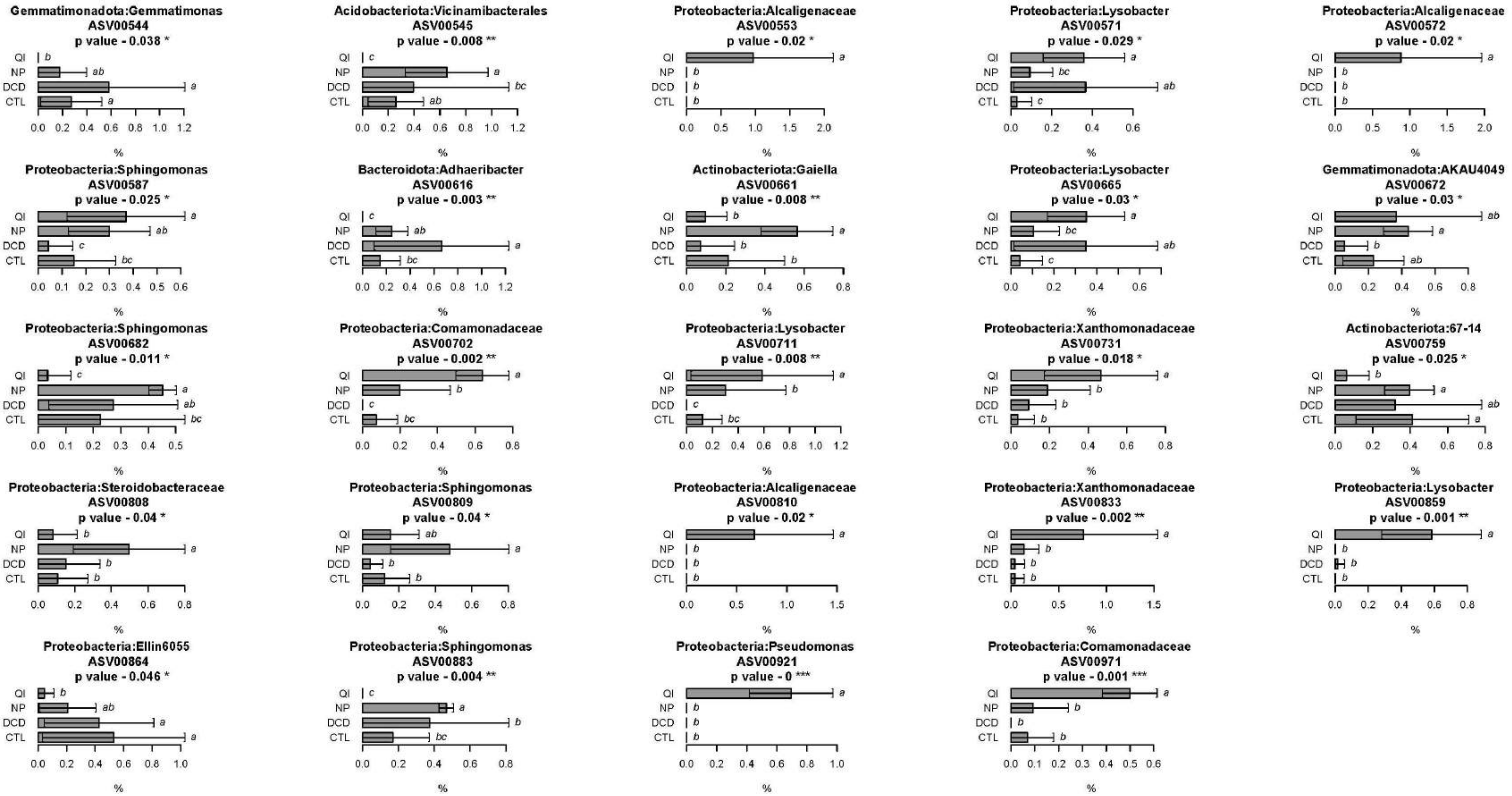

FIG S12d Barplots of the statistically significant differentially abundant bacterial ASVs in samples of the alkaline soil treated (DCD, NP, QI high dose) or not treated with the NIs (CTL). The corresponding p-values, ÀSV taxonomies, and treatment relative abundances are provided. Each value is mean of six replicates ± standard error. Per ASV treatment groups designated by the same letter are not significantly different at the selected p -value levels.

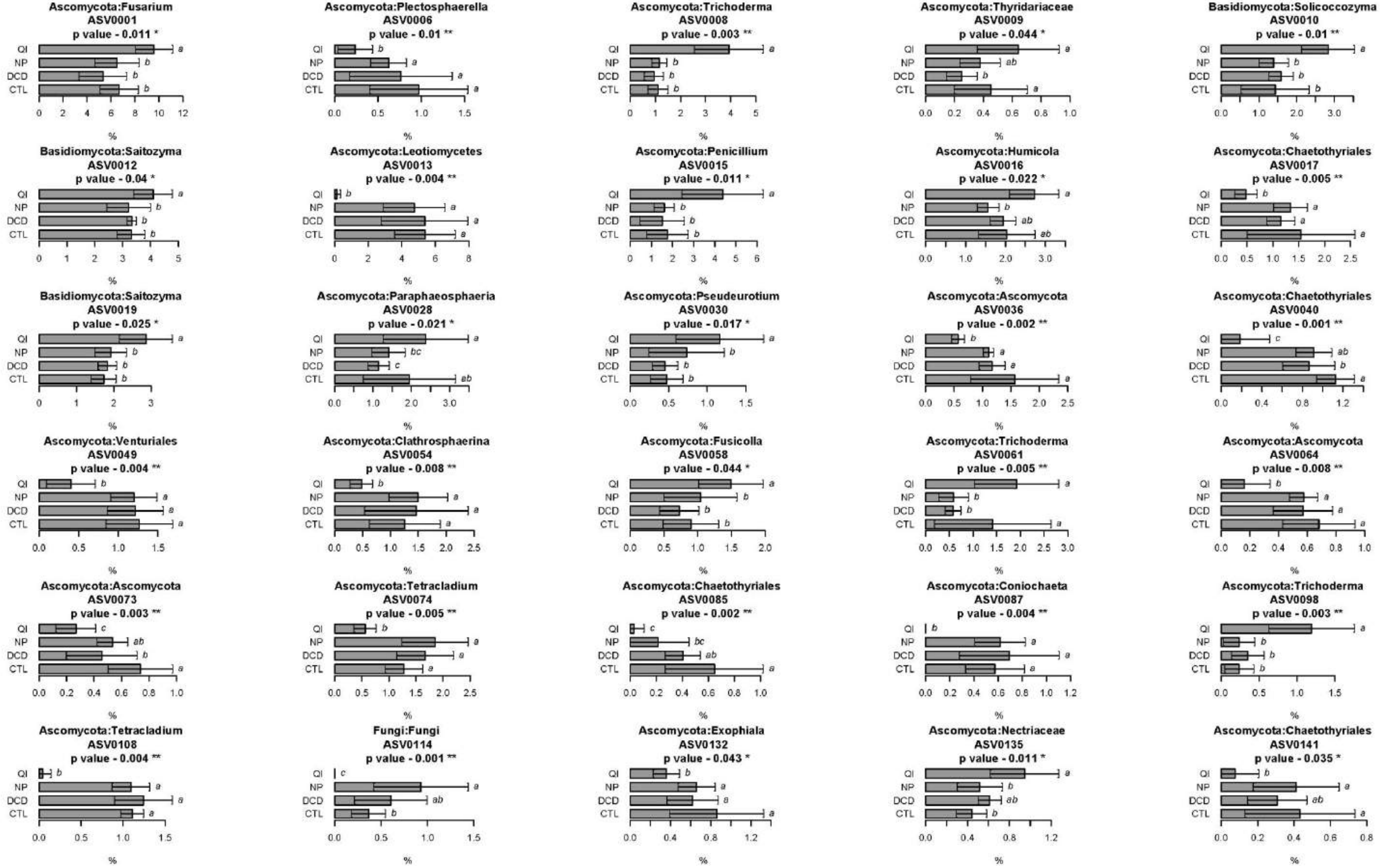

FIG S13a Barplots of the statistically significant differentially abundant fungal ASVs in samples of the acidic soil treated (DCD, NP, QI low dose) or not treated with the NIs (CTL). The corresponding p-values, ÀSV taxonomies, and treatment relative abundances are provided. Each value is mean of six replicates ± standard error. Per ASV treatment groups designated by the same letter are not significantly different at the selected p -value levels.

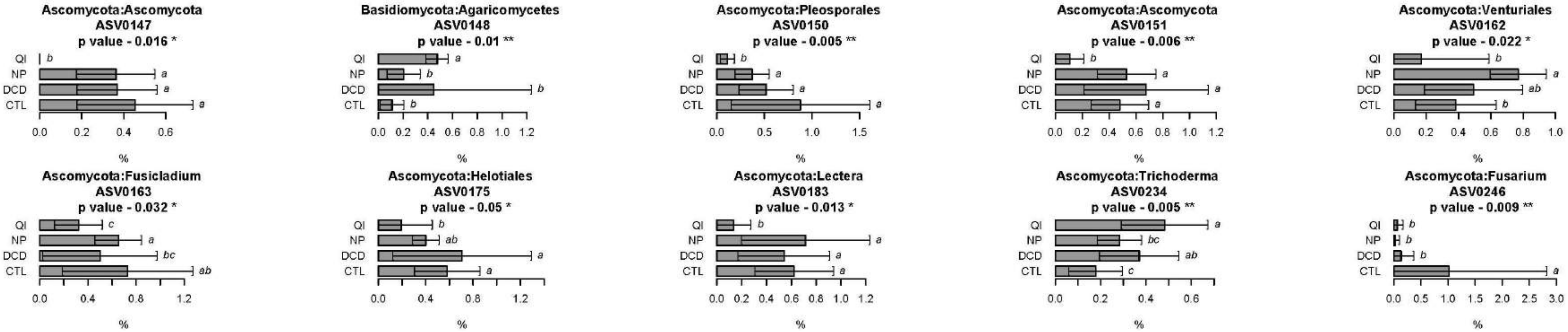

FIG S13b Barplots of the statistically significant differentially abundant fungal ASVs in samples of the acidic soil treated (DCD, NP, QI low dose) or not treated with the NIs (CTL). The corresponding p-values, ÀSV taxonomies, and treatment relative abundances are provided. Each value is mean of six replicates ± standard error. Per ASV treatment groups designated by the same letter are not significantly different at the selected p -value levels.

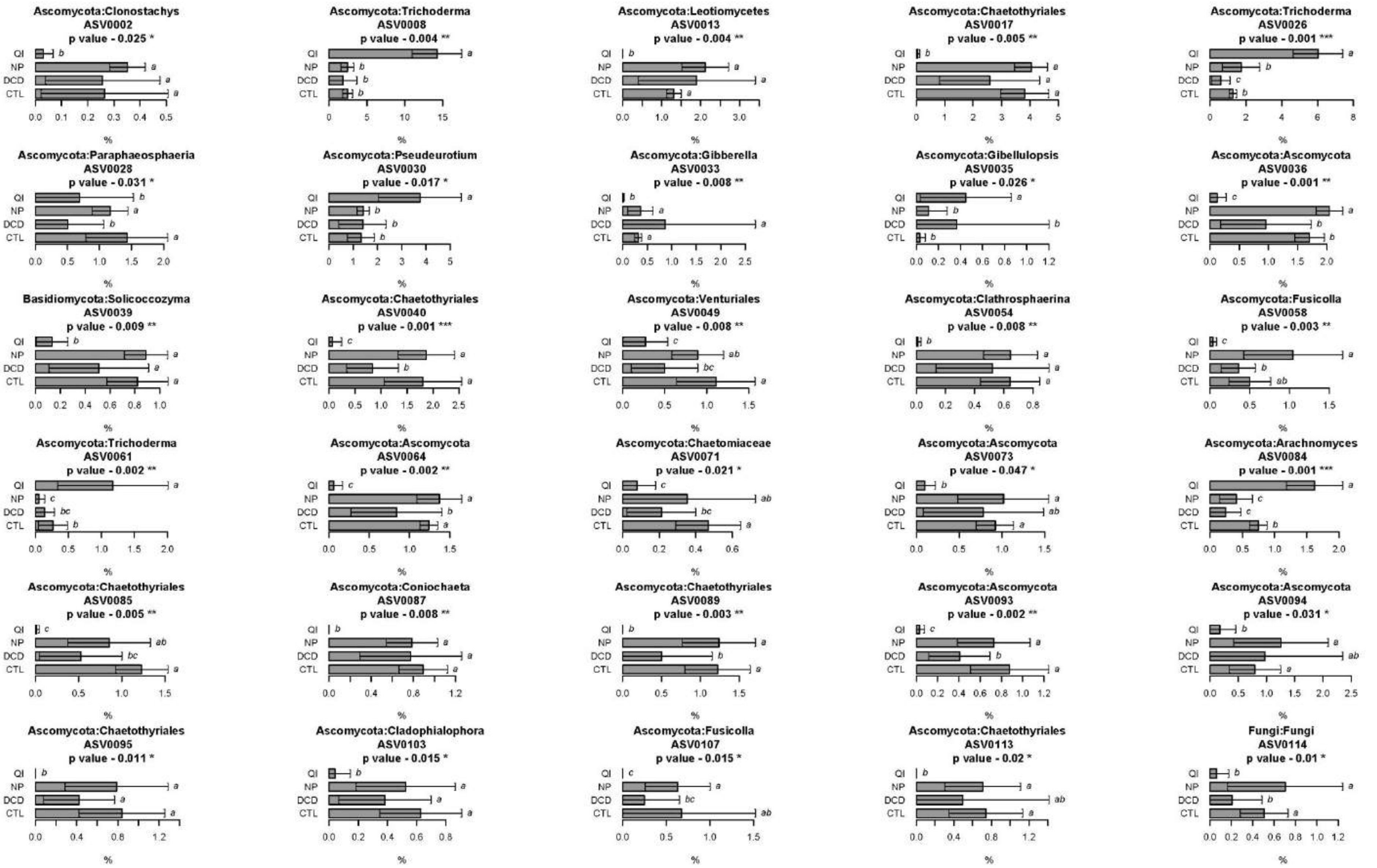

FIG S14a Barplots of the statistically significant differentially abundant fungal ASVs in samples of the acidic soil treated (DCD, NP, QI high dose) or not treated with the NIs (CTL). The corresponding p-values, ÀSV taxonomies, and treatment relative abundances are provided. Each value is mean of six replicates ± standard error. Per ASV treatment groups designated by the same letter are not significantly different at the selected p -value levels.

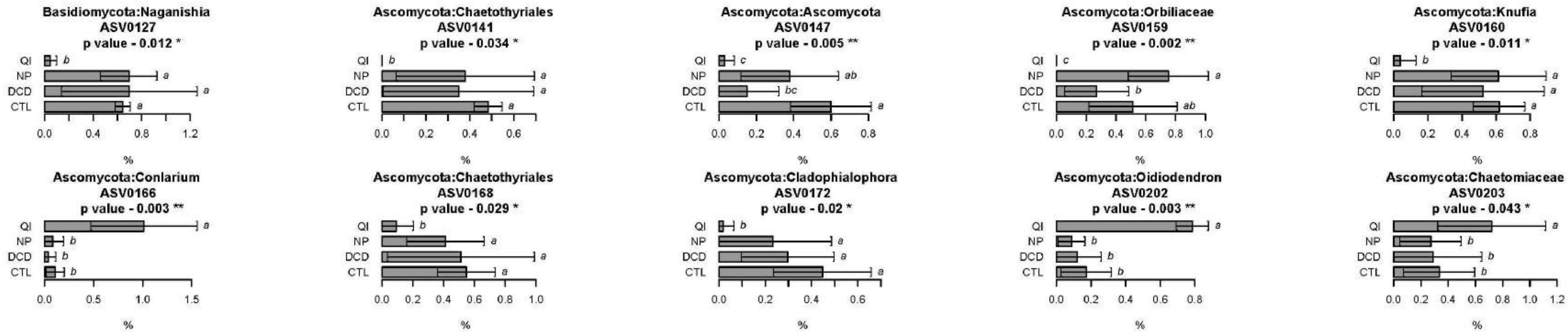

FIG S14b Barplots of the statistically significant differentially abundant fungal ASVs in samples of the acidic soil treated (DCD, NP, QI high dose) or not treated with the NIs (CTL). The corresponding p-values, ÀSV taxonomies, and treatment relative abundances are provided. Each value is mean of six replicates ± standard error. Per ASV treatment groups designated by the same letter are not significantly different at the selected p -value levels.

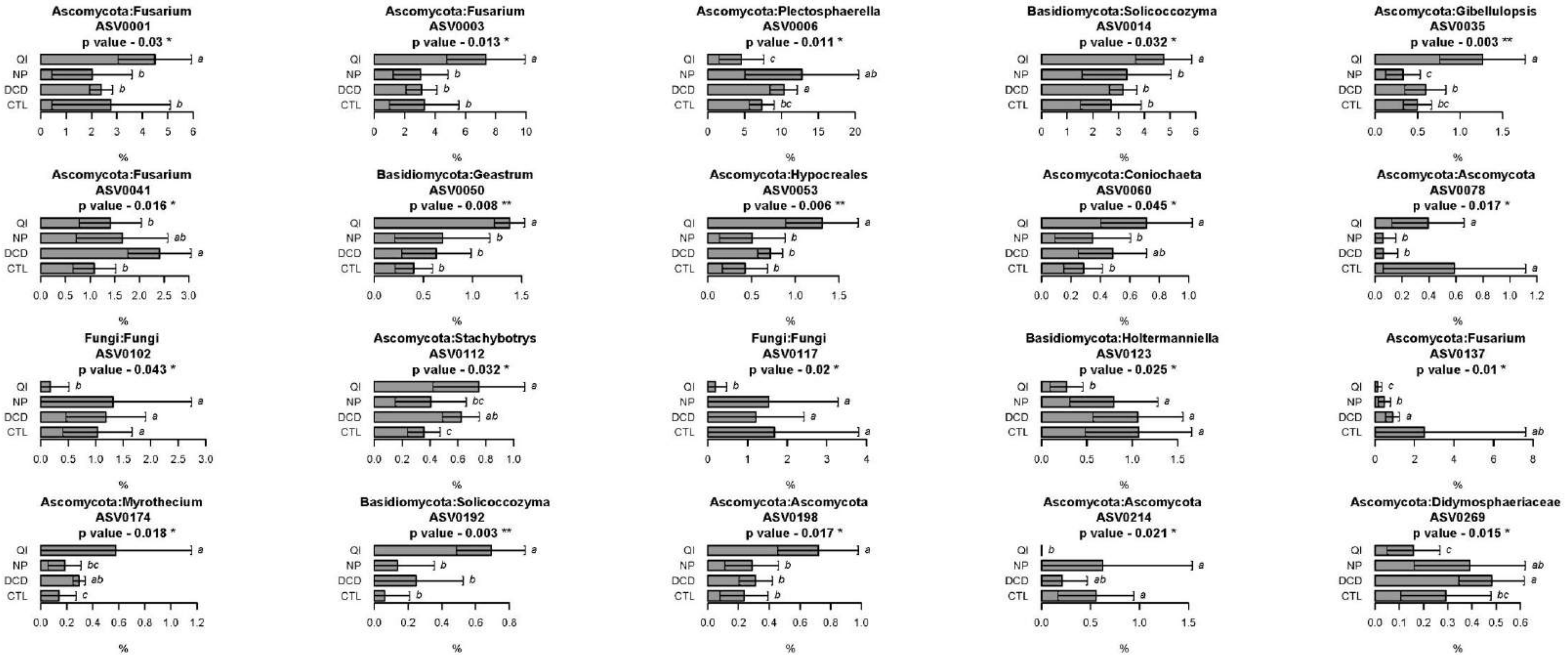

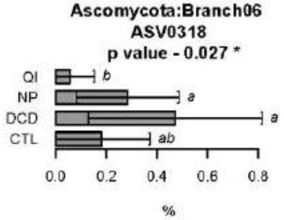

FIG S15 Barplots of the statistically significant differentially abundant fungal ASVs in samples of the alkaline soil treated (DCD, NP, QI low dose) or not treated with the NIs (CTL). The corresponding p-values, ÀSV taxonomies, and treatment relative abundances are provided. Each value is mean of six replicates ± standard error. Per ASV treatment groups designated by the same letter are not significantly different at the selected p -value levels.

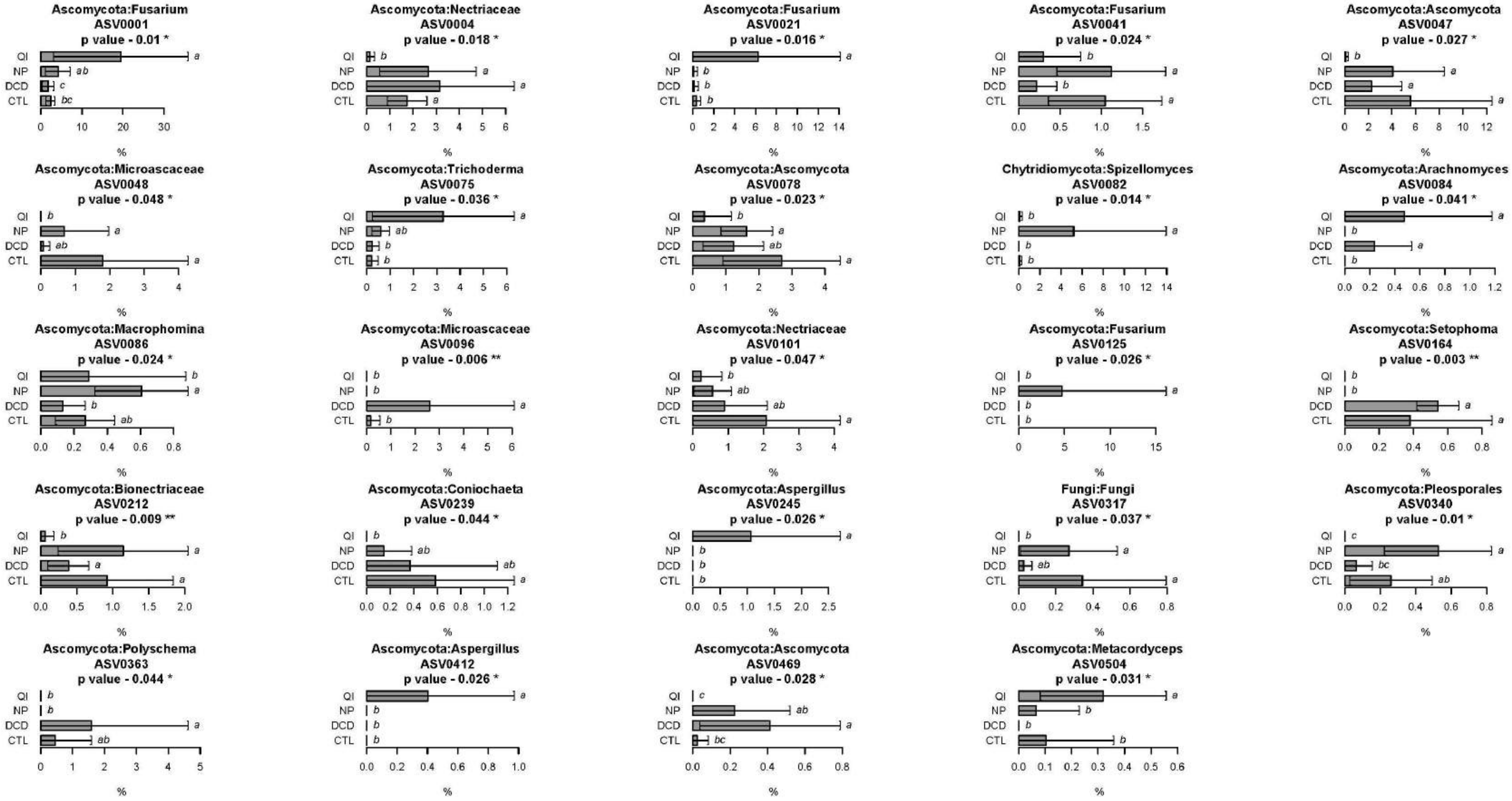

FIG S16 Barplots of the statistically significant differentially abundant fungal ASVs in samples of the alkaline soil treated (DCD, NP, QI high dose) or not treated with the NIs (CTL). The corresponding p-values, ÀSV taxonomies, and treatment relative abundances are provided. Each value is mean of six replicates ± standard error. Per ASV treatment groups designated by the same letter are not significantly different at the selected p -value levels.

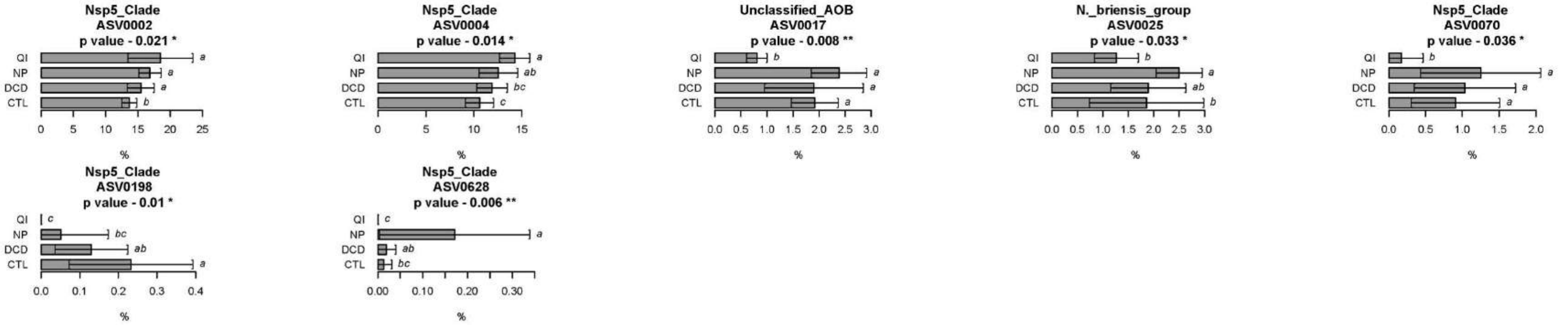

FIG S17 Barplots of the statistically significant differentially abundant AOB ASVs in samples of the acidic soil treated (DCD, NP, QI low dose) or not treated with the NIs (CTL). The corresponding p-values, ÀSV taxonomies, and treatment relative abundances are provided. Each value is mean of six replicates ± standard error. Per ASV treatment groups designated by the same letter are not significantly different at the selected p -value levels.

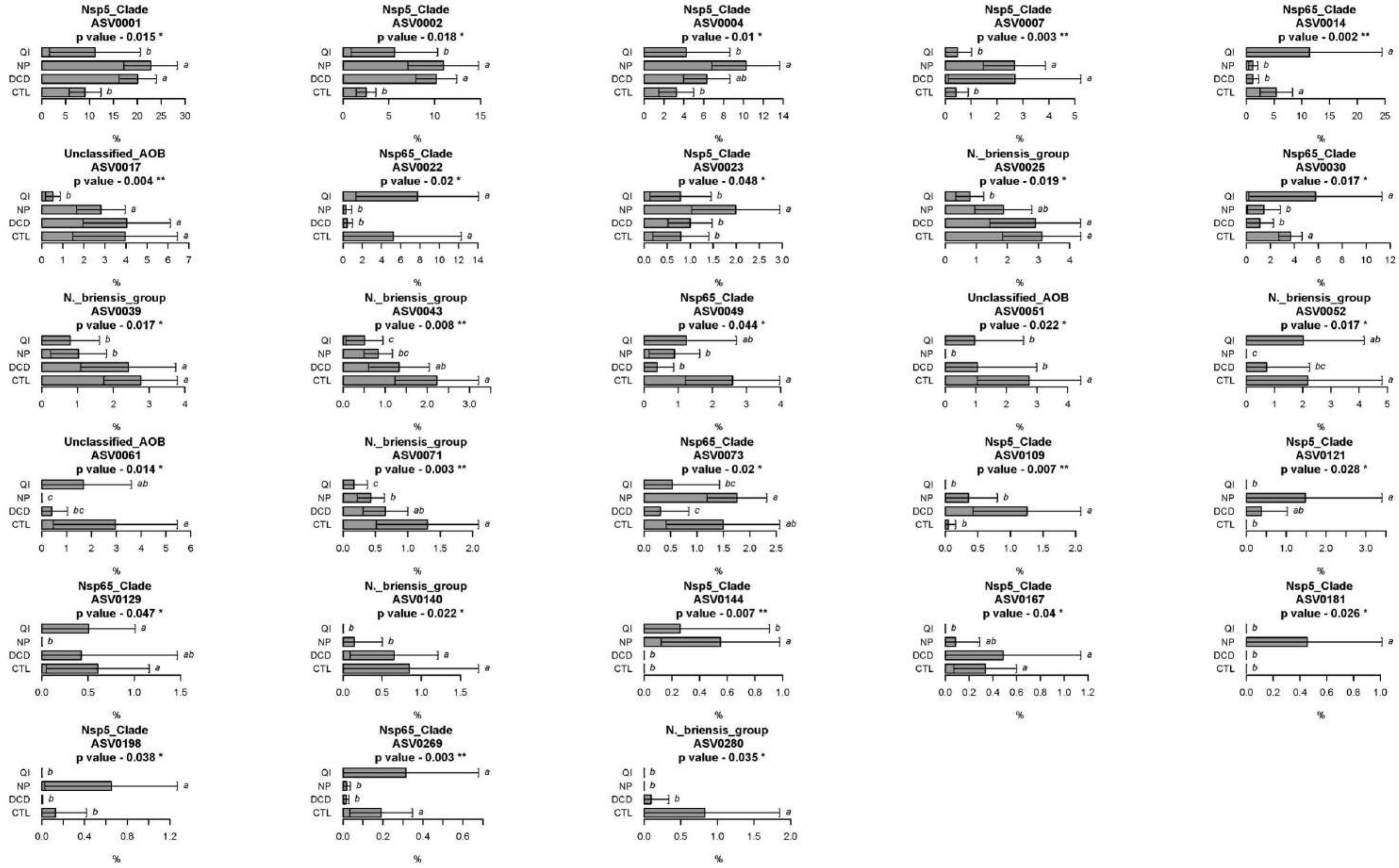

FIG S18 Barplots of the statistically significant differentially abundant AOB ASVs in samples of the acidic soil treated (DCD, NP, QI high dose) or not treated with the NIs (CTL). The corresponding p-values, ÀSV taxonomies, and treatment relative abundances are provided. Each value is mean of six replicates ± standard error. Per ASV treatment groups designated by the same letter are not significantly different at the selected p -value levels.

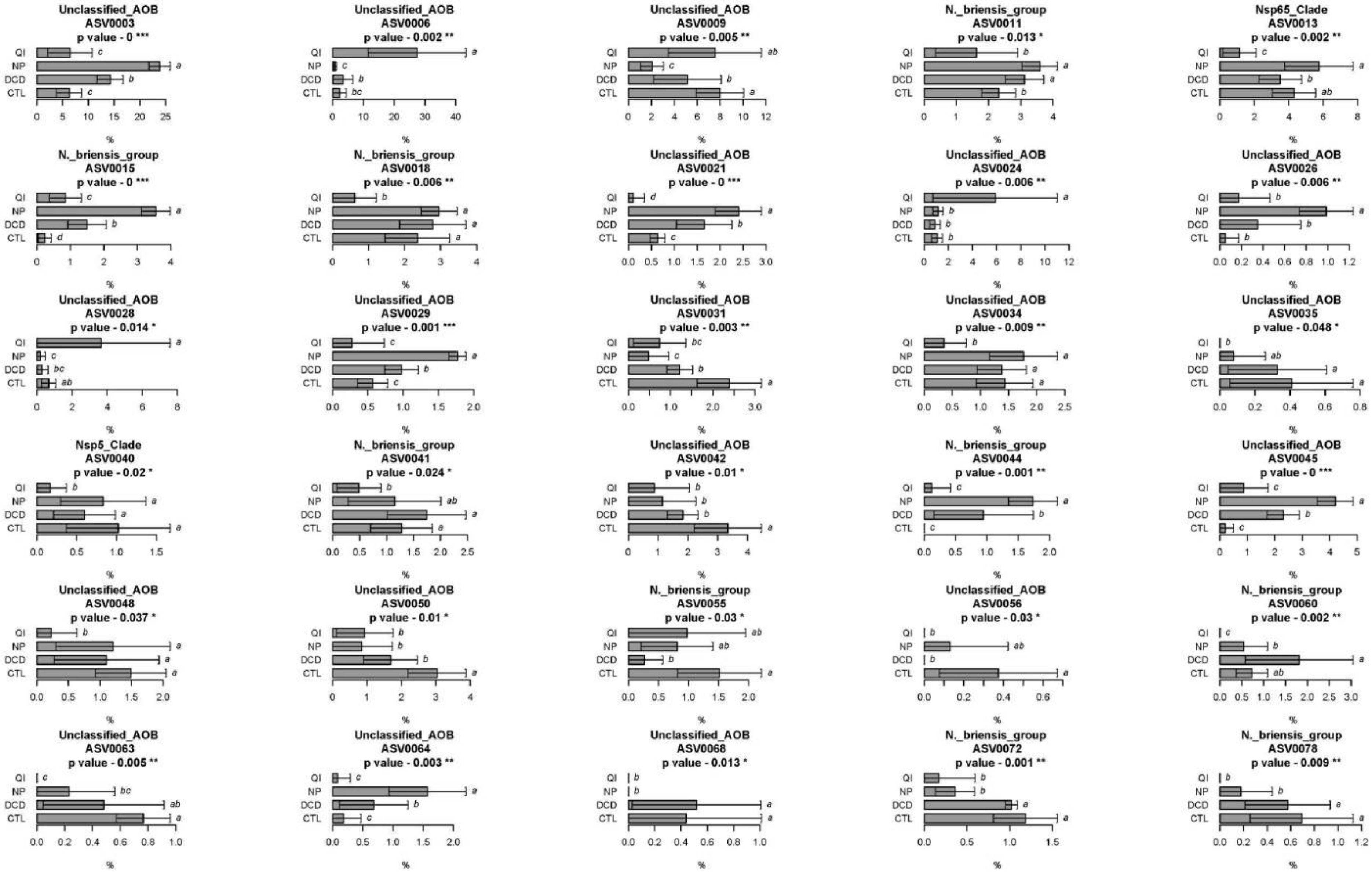

FIG S19a Barplots of the statistically significant differentially abundant AOB ASVs in samples of the alkaline soil treated (DCD, NP, QI low dose) or not treated with the NIs (CTL). The corresponding p-values, ÀSV taxonomies, and treatment relative abundances are provided. Each value is mean of six replicates ± standard error. Per ASV treatment groups designated by the same letter are not significantly different at the selected p -value levels.

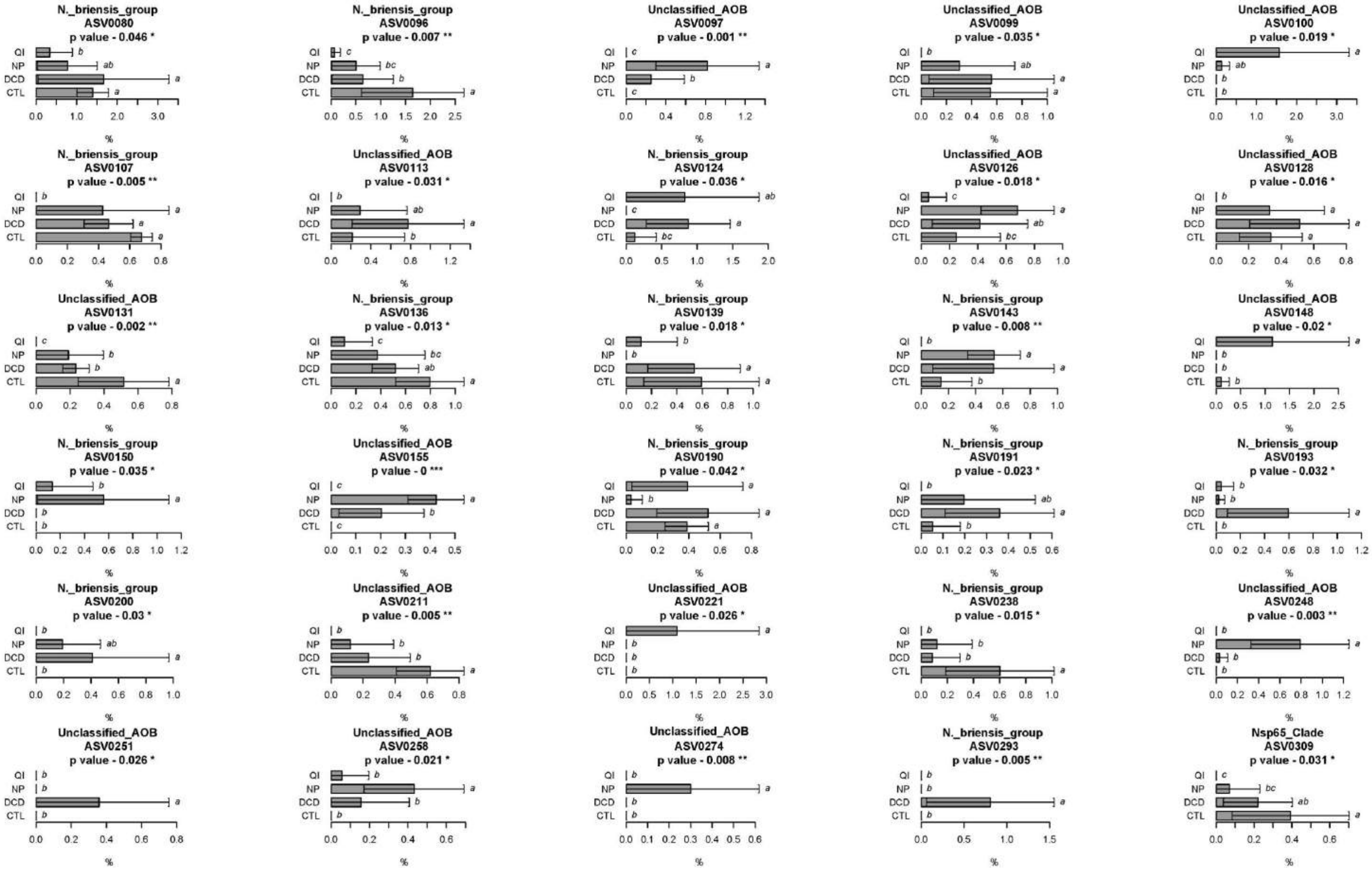

FIG S19b Barplots of the statistically significant differentially abundant AOB ASVs in samples of the alkaline soil treated (DCD, NP, QI low dose) or not treated with the NIs (CTL). The corresponding p-values, ÀSV taxonomies, and treatment relative abundances are provided. Each value is mean of six replicates ± standard error. Per ASV treatment groups designated by the same letter are not significantly different at the selected p -value levels.

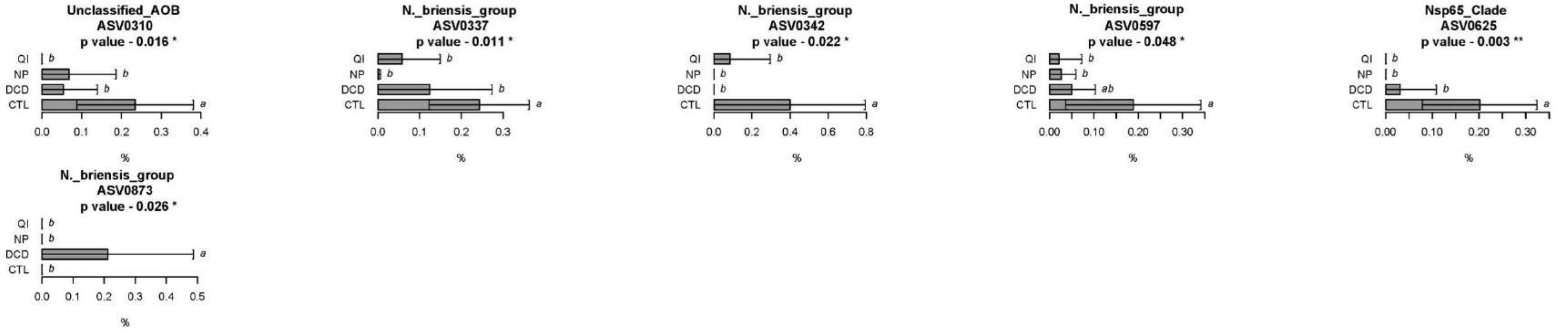

FIG S19c Barplots of the statistically significant differentially abundant AOB ASVs in samples of the alkaline soil treated (DCD, NP, QI low dose) or not treated with the NIs (CTL). The corresponding p-values, ÀSV taxonomies, and treatment relative abundances are provided. Each value is mean of six replicates ± standard error. Per ASV treatment groups designated by the same letter are not significantly different at the selected p -value levels.

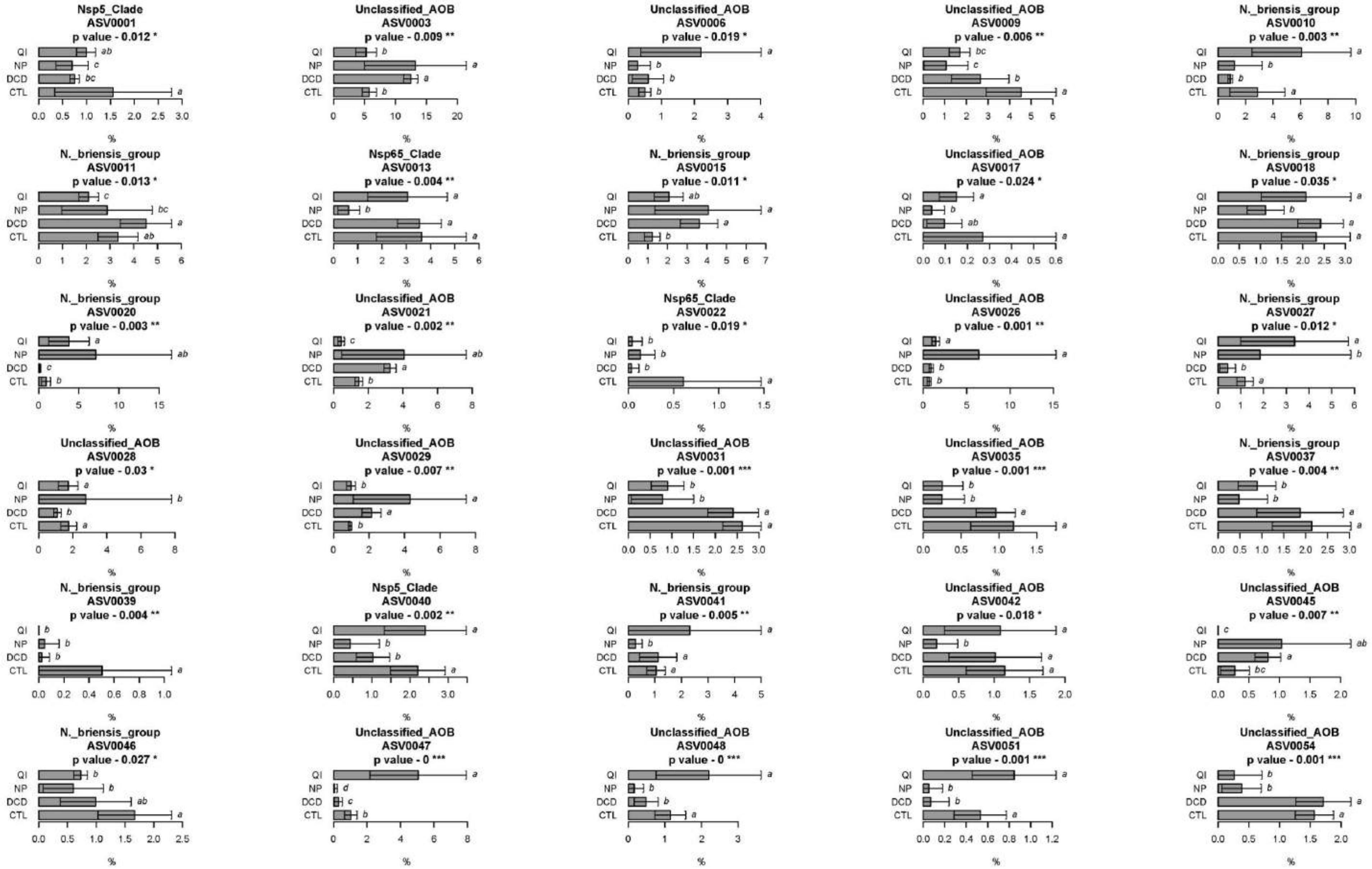

FIG S20a Barplots of the statistically significant differentially abundant AOB ASVs in samples of the alkaline soil treated (DCD, NP, QI high dose) or not treated with the NIs (CTL). The corresponding p-values, ÀSV taxonomies, and treatment relative abundances are provided. Each value is mean of six replicates ± standard error. Per ASV treatment groups designated by the same letter are not significantly different at the selected p -value levels.

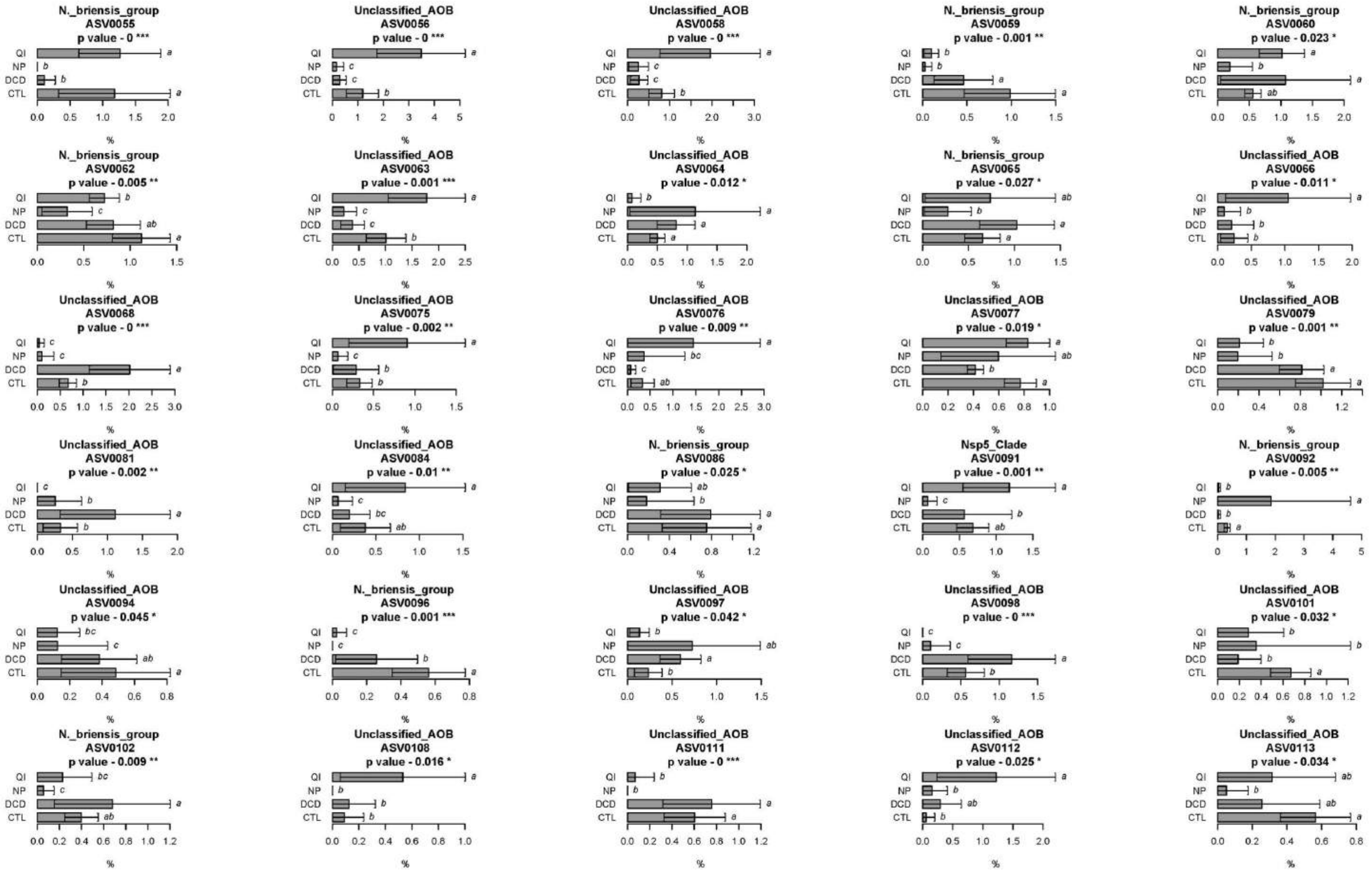

FIG S20b Barplots of the statistically significant differentially abundant AOB ASVs in samples of the alkaline soil treated (DCD, NP, QI high dose) or not treated with the NIs (CTL). The corresponding p-values, ÀSV taxonomies, and treatment relative abundances are provided. Each value is mean of six replicates ± standard error. Per ASV treatment groups designated by the same letter are not significantly different at the selected p -value levels.

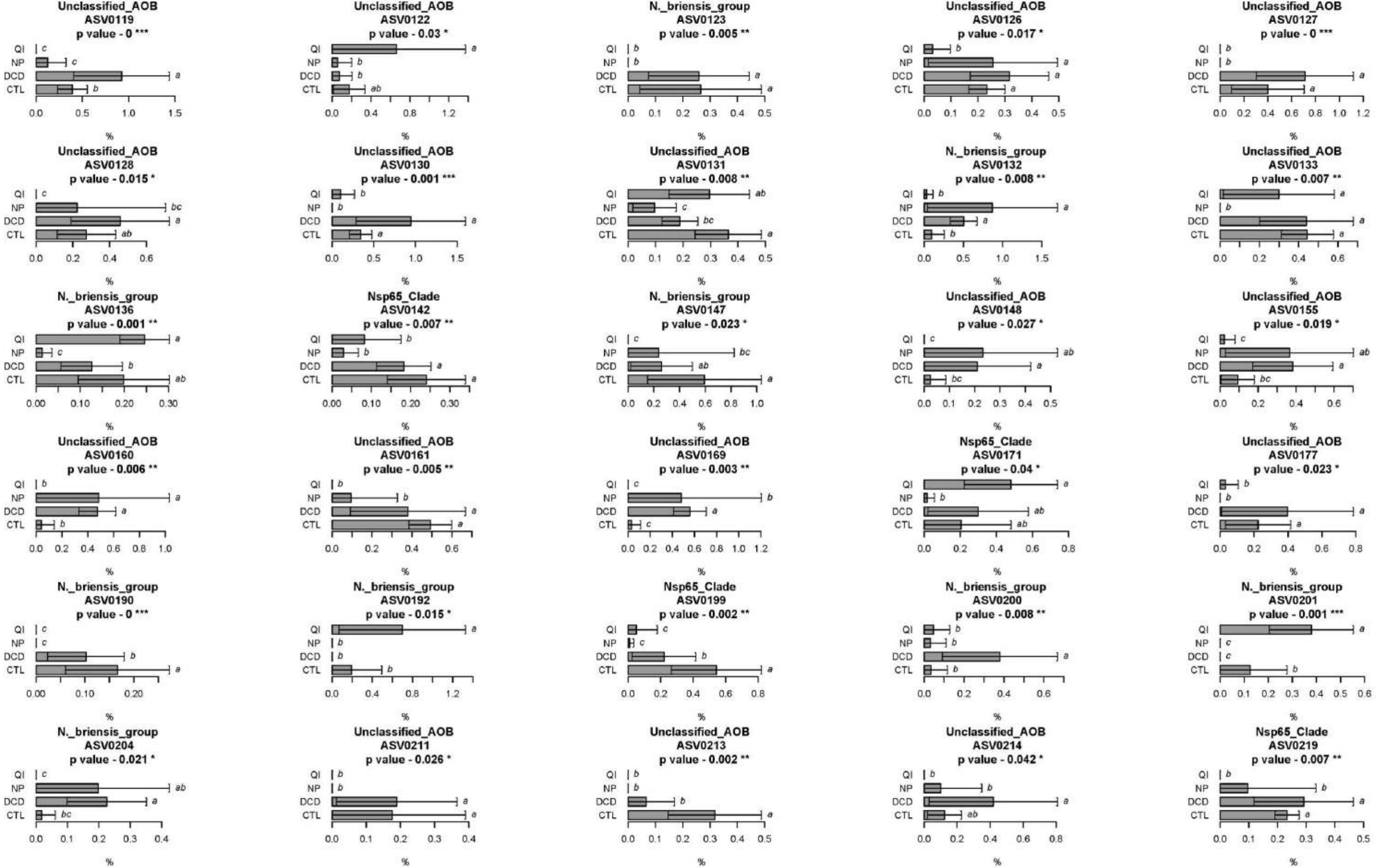

FIG S20c Barplots of the statistically significant differentially abundant AOB ASVs in samples of the alkaline soil treated (DCD, NP, QI high dose) or not treated with the NIs (CTL). The corresponding p-values, ÀSV taxonomies, and treatment relative abundances are provided. Each value is mean of six replicates ± standard error. Per ASV treatment groups designated by the same letter are not significantly different at the selected p -value levels.

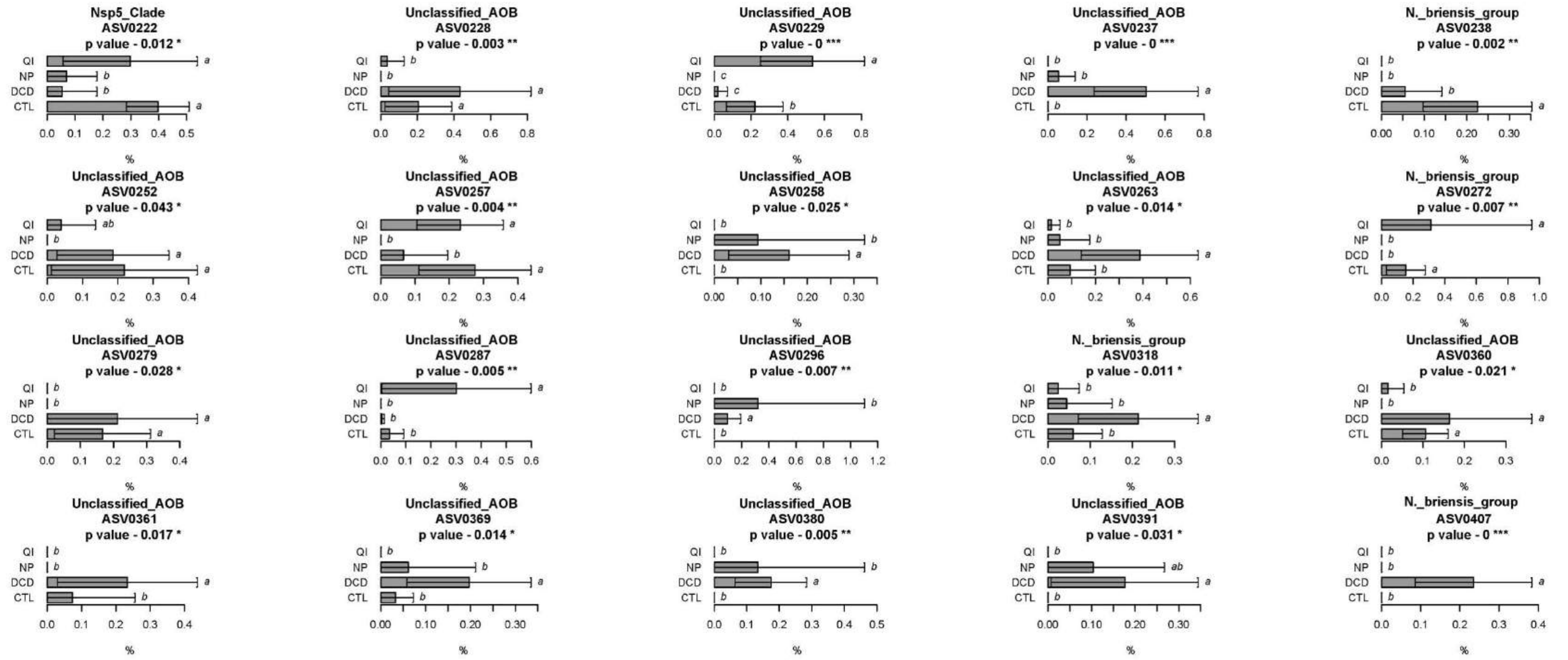

FIG S20d Barplots of the statistically significant differentially abundant AOB ASVs in samples of the alkaline soil treated (DCD, NP, QI high dose) or not treated with the NIs (CTL). The corresponding p-values, ÀSV taxonomies, and treatment relative abundances are provided. Each value is mean of six replicates ± standard error. Per ASV treatment groups designated by the same letter are not significantly different at the selected p -value levels.

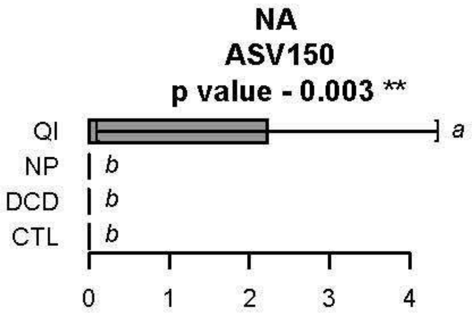

FIG S21 Barplots of the statistically significant differentially abundant AOA ASVs in samples of the acidic soil treated (DCD, NP, QI high dose) or not treated with the NIs (CTL). The corresponding p-values, ÀSV taxonomies, and treatment relative abundances are provided. Each value is mean of six replicates ± standard error. Per ASV treatment groups designated by the same letter are not significantly different at the selected p - value levels.

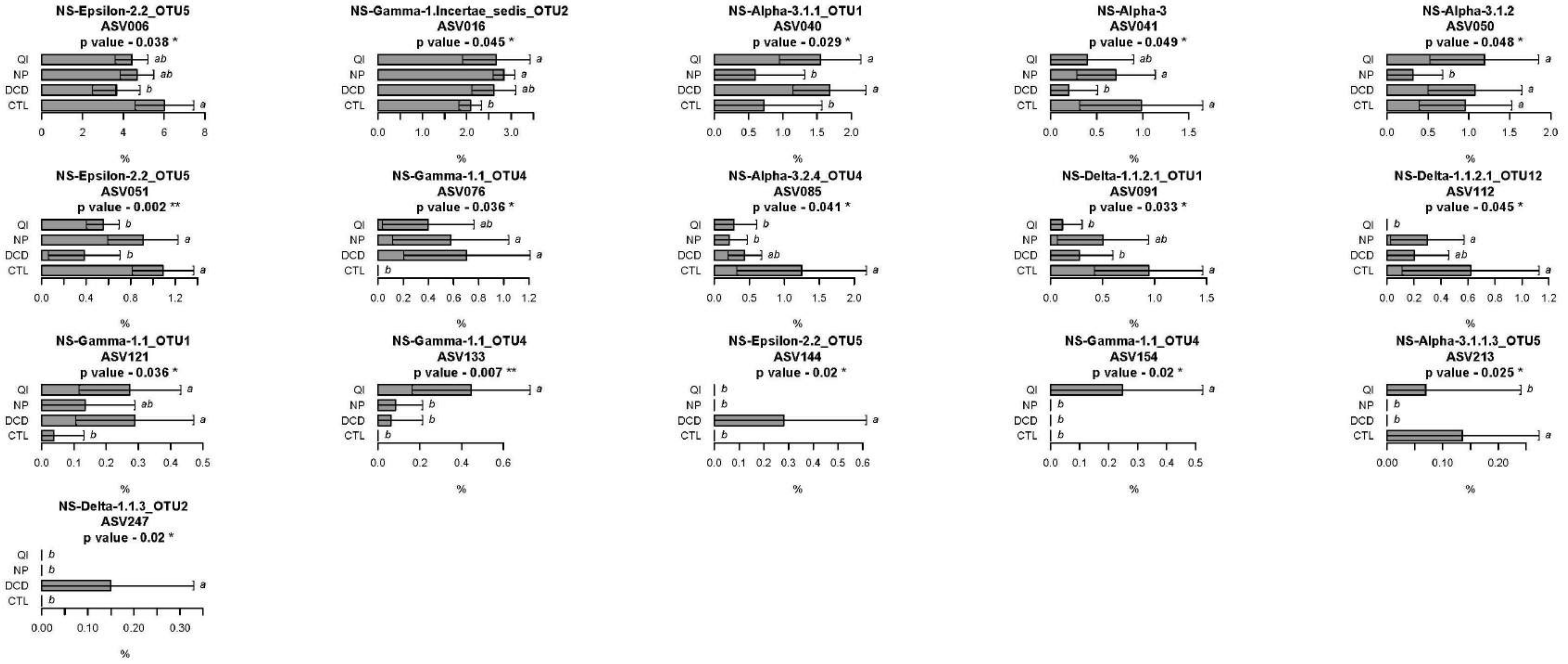

FIG S22 Barplots of the statistically significant differentially abundant AOA ASVs in samples of the alkaline soil treated (DCD, NP, QI low dose) or not treated with the NIs (CTL). The corresponding p-values, ÀSV taxonomies, and treatment relative abundances are provided. Each value is mean of six replicates ± standard error. Per ASV treatment groups designated by the same letter are not significantly different at the selected p -value levels.

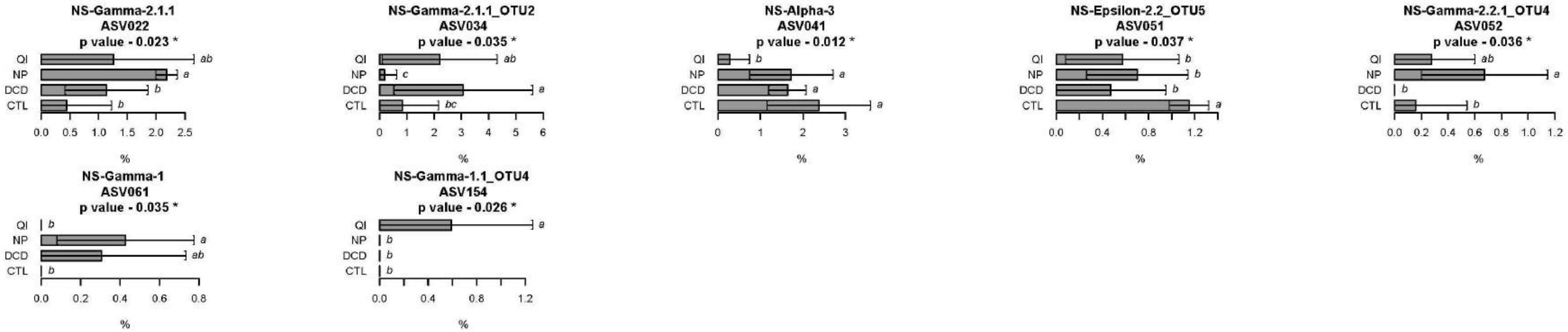

FIG S23 Barplots of the statistically significant differentially abundant AOA ASVs in samples of the alkaline soil treated (DCD, NP, QI high dose) or not treated with the NIs (CTL). The corresponding p-values, ÀSV taxonomies, and treatment relative abundances are provided. Each value is mean of six replicates ± standard error. Per ASV treatment groups designated by the same letter are not significantly different at the selected p -value levels.

REFERENCES

1. Rotthauwe JH, Witzel KP, Liesack W. 1997. The ammonia monooxygenase structural gene amoA as a functional marker: molecular fine-scale analysis of natural ammonia-oxidizing populations. Appl Environ Microbiol 63: 4704–4712. https://doi.org/10.1128/aem.63.12.4704-4712.1997.
2. Francis CA, Roberts KJ, Beman JM, Santoro AE, Oakley BB. 2005. Ubiquity and diversity of ammonia-oxidizing archaea in water column sand sediments of the ocean. PNAS USA 102: 14683–14688. https://doi.org/10.1073/pnas.0506625102.
3. Pjevac P, Schauberger C, Poghosyan L, Herbold CW, van Kessel MAHJ, Daebeler A, Steinberger M, Jetten MSM, Lücker S, Wagner M, Daims H. 2017. AmoA-targeted polymerase chain reaction primers for the specific detection and quantification of comammox Nitrospira in the environment. Front Microbiol 8:1508. https://doi.org/10.3389/fmicb.2017.01508.
4. Pester M, Maixer F, Berry D, Rattel T, Koch H, Lucker S, Nowka B, Richter A, Spieck E, Lebedeva E, Loy A, Wagner M, Daims H. 2014. NxrB encoding the beta subunit of nitrite oxidoreductase as functional and phylogenetic marker for nitrite-oxidizing Nitrospira. Environ Microbiol 16:3055–3071. https://doi.org/10.1111/1462-2920.12300.
5. Vanparys B, Spieck E, Heylen K, Wittebolle L, Geets J, Boon N, De Vos P. 2007. The phylogeny of the genus Nitrobacter based on comparative rep-PCR, 16S rRNA and nitrite oxidoreductase gene sequence analysis. Syst Appl Microbiol 30: 297–308. https://doi.org/10.1016/j.syapm.2006.11.006.
6. Fierer N, Jackson JA, Vilgalys R, Jackson R.B. 2005. Assessment of soil microbial community structure by use of taxon-specific quantitative PCR assays. Appl Environ Microbiol 71: 4117–4120. https://doi.org/10.1128/AEM.71.7.4117-4120.2005.
7. Ochsenreiter T, Selezi D, Quaiser A, Bonch-Osmolovskaya L, Schleper C., 2003. Diversity and abundance of Crenarchaeota in terrestrial habitats studied by 16S RNA surveys and real time PCR. Environ Microbiol 5: 787-797. https://doi.org/10.1046/j.1462-2920.2003.00476.x

8. Chemidlin Prévost-Bouré N, Christen R, Dequiedt S, Mougel C, Lelièvre M, Jolivet C, Shahbazkia HR., Guillou L, Arrouays D, Ranjard L. 2011. Validation and application of a PCR primer set to quantify fungal communities in the soil environment by Real-Time Quantitative PCR. PLoS One 6 : e24166. https://doi.org/10.1371/journal.pone.0024166.
9. Walters W, Hyde ER, Berg-Lyons D, Ackermann G, Humphrey G, Parada A, Gilbert JA, Jansson JK, Caporaso JG, Fuhrman JA, Apprill A, Knight R. 2016. Improved bacterial 16S rRNA gene (V4 and V4-5) and fungal internal transcribed spacer marker gene primers for microbial community surveys. mSystems 1: e00009-15. https://doi.org/10.1128/mSystems.00009-15.
10. Ihrmark K, Bödeker ITM, Cruz-Martinez K, Friberg H, Kubartova A, Schenck J, Strid Y, Stenlid J, Brandström-Durling M, Clemmensen KE, Lindahl BD. 2012. New primers to amplify the fungal ITS2 region – evaluation by 454-sequencing of artificial and natural communities. FEMS Microbiol Ecol 82: 666-677. https://doi.org/10.1111/j.1574-6941.2012.01437.x.
11. White T, Bruns T, Lee S, Taylor J. 1990. Amplification and direct sequencing of fungal ribosomal RNA genes for phylogenetics, p 315–322. In Innis, M., Gelfand, D., Shinsky, J., White, T., (ed), PCR protocols: a guide to methods and applications. Academic Press, London, United Kingdom.
12. Marusenko Y, Bates ST, Anderson I, Johnson SL, Soule T, Garcia-Pichel F. 2013. Ammonia-oxidizing archaea and bacteria are structured by geography in biological soil crusts across North American arid lands. Ecol Process 2: 9. https://doi.org/10.1186/2192-1709-2-9.
